# Qualitative and Quantitative Analysis of Chemical Components in Yinhua Pinggan Granule with High-Performance Liquid Chromatography Coupled with Q-Exactive Mass Spectrometry

**DOI:** 10.3390/molecules29102300

**Published:** 2024-05-14

**Authors:** Imranjan Yalkun, Haofang Wan, Lulu Ye, Li Yu, Yu He, Chang Li, Haitong Wan

**Affiliations:** Zhejiang Chinese Medical University, Hangzhou 310053, China

**Keywords:** Yinhua Pinggan Granule, HPLC–Q-Exactive MS, chemical components, quantitative analysis, quality control of TCM

## Abstract

Yinhua Pinggan Granule (YPG) is an approved compounded traditional Chinese medicine (TCM) prescription for the treatment of cold, cough, viral pneumonia, and related diseases. Due to its complicated chemical composition, the material basis of YPG has not been systematically investigated. In this study, an analytical method based on high-performance liquid chromatography (HPLC) coupled with Q-Exactive mass spectrometry was established. Together with the help of a self-built compound database and Compound Discoverer software 3.1, the chemical components in YPG were tentatively identified. Subsequently, six main components in YPG were quantitatively characterized with a high-performance liquid chromatography–diode array detector (HPLC-DAD) method. As a result, 380 components were annotated, including 19 alkaloids, 8 organic acids, 36 phenolic acids, 27 other phenols, 114 flavonoids, 75 flavonoid glycoside, 72 terpenes, 11 anthraquinones, and 18 other compounds. Six main components, namely, chlorogenic acid, puerarin, 3′-methoxypuerarin, polydatin, glycyrrhizic acid, and emodin, were quantified simultaneously. The calibration curves of all six analytes showed good linearity (R^2^ > 0.9990) within the test ranges. The precision, repeatability, stability, and recovery values were all in acceptable ranges. In addition, the total phenol content and DPPH scavenging activity of YPG were also determined. The systematic elucidation of the chemical components in YPG in this study may provide clear chemical information for the quality control and pharmacological research of YPG and related TCM compounded prescriptions.

## 1. Introduction

Yinhua Pinggan Granule (YPG) is a patent compounded traditional Chinese medicine (TCM) prescription approved by the State Food and Drug Administration of China (No. Z20133007) for the treatment of cold, cough, viral pneumonia, and other related diseases. YPG was originally developed from an ancient TCM formula, Ma-huang-Tang (Ephedra Decoction), which was recorded in the TCM classic Shang Han Lun (Treatise on Febrile Diseases). YPG is composed of six herbs, namely, *Lonicerae Japonicae Flos*, *Puerariae Lobatae Radix*, *Polygoni Cuspidati Rhizoma Et Radix*, *Ephedrae Herba*, *Glycyrrhizae Radix Et Rhizoma*, and *Armeniacae Semen Amarum*, in a ratio of 4:4:4:2:2:1. In recent years, pharmacological and clinical studies of YPG have made effective progress. Previous studies have shown that YPG can inhibit the replication of influenza virus and regulate the occurrence of apoptosis caused by influenza virus infection [1]. In addition, YPG has a significant antiviral effect on H1N1 influenza virus-infected RAW264.7 cells and can protect influenza virus-infected pneumonia mice by reducing their lung injury [2]. At the same time, recent research showed that YPG and its components have significant inhibitory effects on the proliferation of the H1N1 virus [3]. Meanwhile, a randomized, double-blind, parallel, controlled clinical trial program with a total of 240 participants is in progress to test the clinical efficacy of YPG as a complementary therapy against community-acquired drug-resistant bacterial pneumonia [4].

TCM, including YPG, has shown unique advantages in the treatment of influenza virus and related diseases [5,6,7], especially during the COVID-19 pandemic [8,9,10]. However, the chemical complexity of TCM not only leads to great challenges in elucidating the pharmacological mechanisms of TCM but also hinders the deciphering of the material basis and quality control [11]. Therefore, the chemical profiling of compounded TCM prescriptions is of great importance. With its high sensitivity and high resolution, the liquid chromatography–mass spectrometry (LC-MS) technique has been widely applied in the qualitative analysis of TCM products in recent years [12,13].

Considering that most research on YPG has focused on pharmacology and clinical efficacy, the present study aimed to systematically identify and quantitatively characterize the phytochemical constituents of YPG. Based on the HPLC–Q-Exactive MS system, the global qualitative analysis of YPG was carried out, and then the main representative components were simultaneously quantified with HPLC-DAD detection technology in order to provide a theoretical basis for its future clinical application and quality control of YPG.

## 2. Results and Discussion

### 2.1. Optimization of Chromatographic Conditions

**Figure 1:**
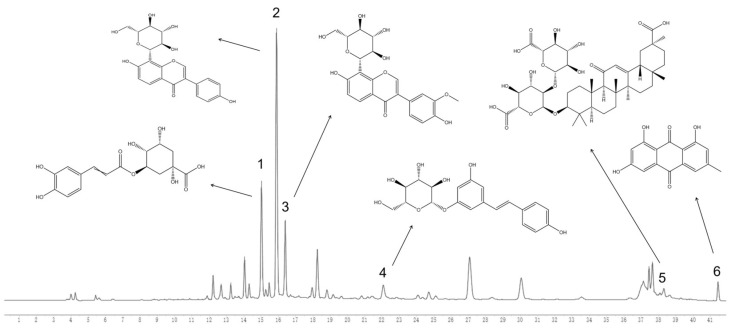
The HPLC chromatogram of YPG (wavelength = 254 nm). **1**. Chlorogenic acid; **2**. puerarin; **3**. 3′-methoxypuerarin; **4**. polydatin; **5**. glycyrrhizic acid; **6**. emodin.

In order to systematically elucidate the chemical components of YPG, both the extraction method (Table S3 in Supporting Information) and the chromatography conditions were optimized. In particular, three different columns based on C18 packing material were tested, namely, the Welch Ultimate XB-C18 column (Welch, Shanghai, China) (150 mm × 4.0 mm, 3.0 μm), Agilent Poroshell 120 EC-C18 column (Agilent, Santa Clara, CA, USA) (150 mm × 3.0 mm, 2.7 μm), and Capcell Pak C18 MG II (Osaka Soda Co., Ltd., Osaka, Japan) (150 mm × 4.6 mm, 3 μm). Compared with the other chromatographic columns, the MG II column exhibited better separation capacity under optimal conditions. The acetonitrile–water system showed a better resolution and response in the selection of the mobile phase, and the addition of 0.1% or 0.5% formic acid could effectively improve the ionization efficiency and peak shape of some compounds. Finally, the HPLC-UV chromatogram obtained under optimized conditions is shown in Figure 1.

### 2.2. Qualitative Analysis of YPG by HPLC–Q-Exactive MS

**Figure 2:**
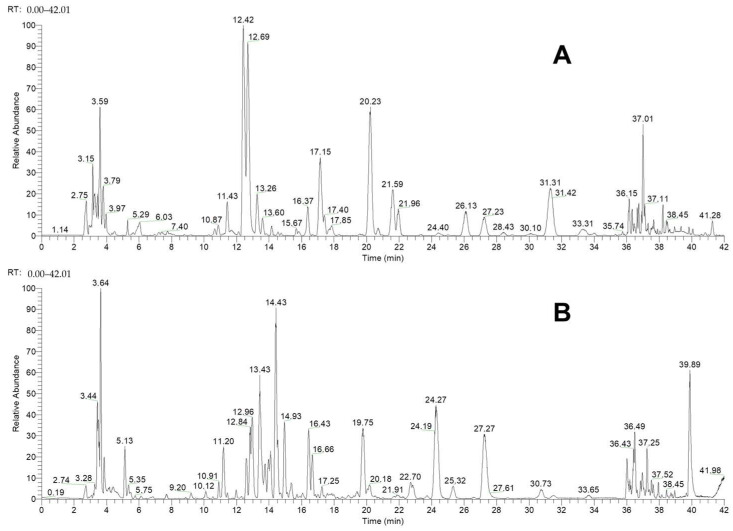
The BPCs of YPG in positive (**A**) and negative (**B**) modes.

Under the optimal chromatographic conditions, the mass spectrometry information of YPG was acquired in both positive and negative modes to cover more components. The base peak chromatograms (BPCs) are presented in Figure 2. Then, as described in Section 3, the data were tentatively annotated with corresponding compounds with the help of Compound Discoverer software according to the values of the accurate molecular weight and patterns of secondary fragmentation [14]. In particular, [M + H]^+^ and [M − H]^−^ were the common quasi-molecular ions in the positive and negative modes, respectively, from which the chemical formulas were preliminarily inferred, with a mass deviation ≤ 5 ppm. After compound library searching, reference comparison, and fragmentation study, a total of 380 components were tentatively identified in YPG. Among them, 45 compounds were also verified by comparison with reference compounds. The mass spectrometry information of the identified compounds is summarized in Table 1 (for additional information, see Table S1 in Supporting Information).

**Figure 3:**
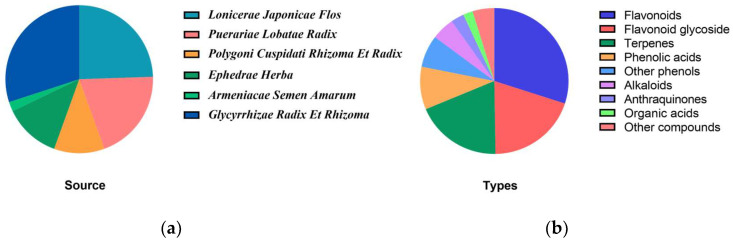
(**a**) The distribution of plant sources tentatively identified in YPG; (**b**) the types of compounds tentatively identified in YPG.

In general, the 380 proposed chemical components of YPG can be classified into 9 categories based on chemical structure, including 19 alkaloids, 8 organic acids, 36 phenolic acids, 27 other phenols, 114 flavonoids, 75 flavonoid glycoside,72 terpenes, 11 anthraquinones and 18 other compounds (Figure 3a). While in the term of plant source, 114 compounds are from *Glycyrrhizae Radix Et Rhizoma*, 93 from *Lonicerae Japonicae Flos*, 76 from *Puerariae Lobatae Radix*, 47 from *Ephedrae Herba*, 42 from *Polygoni Cuspidati Rhizoma Et Radix*, and 8 from *Armeniacae Semen Amarum* (Figure 3b).

### 2.3. Identification of Several Specific Compounds in YPG

Based on the global identification of the components in YPG, the chemical structures of some compounds were inferred from the MS^2^ fragment ions, the proposed fragmentation pathways, and published references.

#### 2.3.1. Phenolic Acids

**Figure 4:**
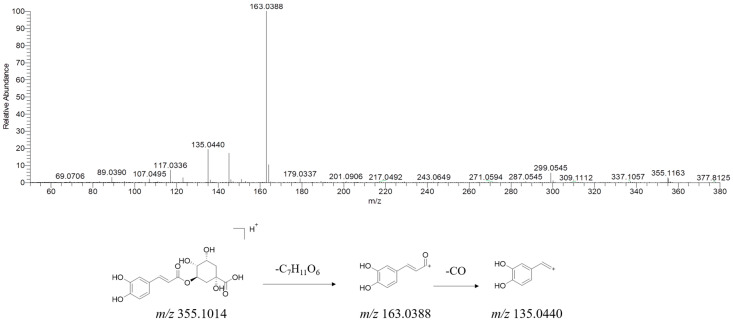
MS/MS spectrum of [M + H]^+^ ions and plausible fragmentation pathway of chlorogenic acid (positive ion mode).

Chlorogenic acid and its isomers, including neochlorogenic acid, cryptochlorogenic acid, etc., are typical phenolic acids found in YPG with high contents [15,16]. As shown in Figure 4, in the positive ion mode, the chlorogenic acid molecule is ionized to produce an [M + H]^+^ peak at *m/z* 355.1014. It is observed that fragmentation occurred via the neutral loss of quinic acid (C_7_H_11_O_6_), resulting in the formation of a product ion at *m/z* 163.0388. Alternatively, the [M + H]^+^ ion may lose a neutral molecule of C_8_H_12_O_7_ to generate a product ion at *m/z* 135.0440.

#### 2.3.2. Flavonoids 

**Figure 5:**
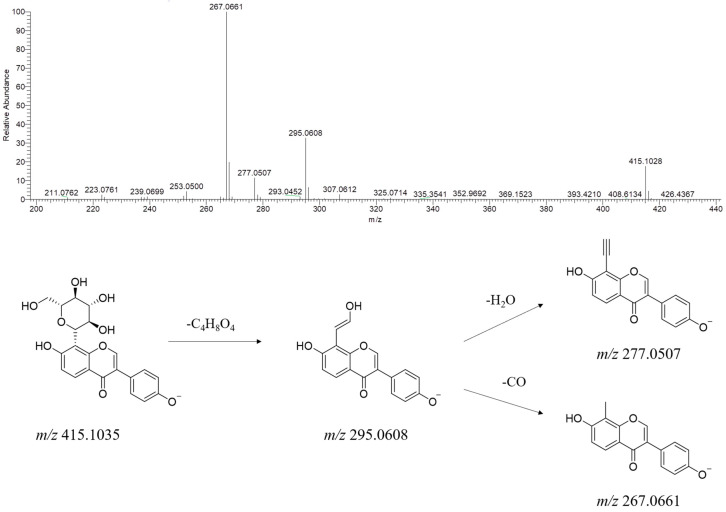
MS/MS spectrum of [M − H]^−^ ions and plausible fragmentation pathway of puerarin (negative ion mode).

In YPG, 114 flavonoids were identified and were the most abundant compound type. As the most basic natural product in plant medicine, flavonoids have a wide variety of biological activities and efficacies [17,18]. In this study, puerarin was taken as an example to illustrate its fragmentation pathway. As a representative compound with high content in YPG, puerarin was observed in multiple characteristic fragmentation patterns in secondary mass spectrometry (Figure 5). The [M − H]^−^ ion (*m/z* 415.1035) of puerarin loses a C_4_H_8_O_4_ moiety to generate a product ion at *m/z* 295.0608. Further neutral loss of H_2_O (18 Da) and CO (28 Da) then produced the fragment ions at *m/z* 277.0507 and 267.0661, respectively. 

#### 2.3.3. Alkaloids

**Figure 6:**
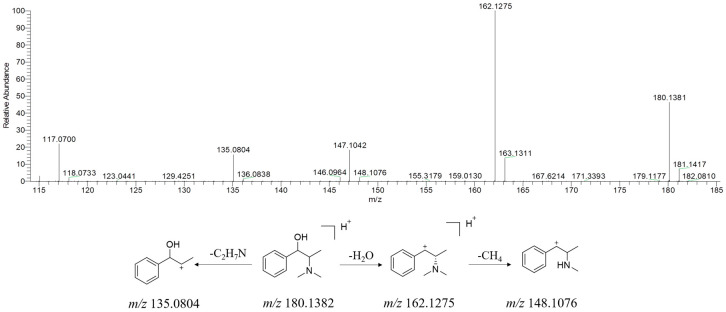
MS/MS spectrum of [M + H]^+^ ions and plausible fragmentation pathway of methylephedrine (positive ion mode).

The alkaloids in YPG are mainly ephedra alkaloids and isomers from *Herba Ephedrae* [19]. As a representative compound, the fragmentation pathway of methylephedrine is discussed herein. As shown in Figure 6, based on the protonated methylephedrine ion at *m/z* 180.1382, three characteristic fragment ions were observed at *m/z* 162.1275, 148.1076, and 138.0804, which can be attributed to [M + H − H_2_O]^+^, [M + H − H_2_O − CH_2_]^+^, and [M + H − C_2_H_7_N]^+^, respectively.

#### 2.3.4. Terpenoids

**Figure 7:**
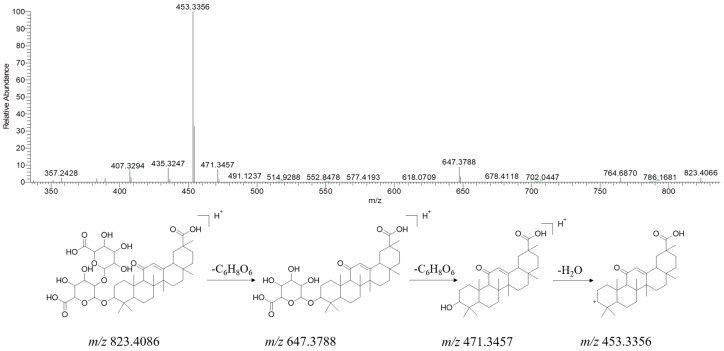
MS/MS spectrum of [M + H]^+^ ions and plausible fragmentation pathway of glycyrrhizic acid (positive ion mode).

A total of 72 terpenoids were tentatively identified in YPG, of which triterpenoid saponins were mainly concentrated in *Lonicera Japonica Thunb*, *Puerariae Lobatae Radix*, and *Glycyrrhizae Radix*. Glycyrrhizic acid is the main active ingredient of *Glycyrrhizae Radix*, and it possesses various pharmacological effects, such as detoxification and anti-inflammatory activities [20,21,22]. As shown in Figure 7, the [M + H]^+^ ion of glycyrrhizic acid at *m/z* 823.4086 subsequently loses two gluconic acid moieties to form fragment ions at *m/z* 647.3788 and 471.3457. The latter ion can further lose one molecule of H_2_O to produce a product ion at *m/z* 453.3356. 

### 2.4. Quantitative Analysis, Total Phenolic Content, and DPPH Radical Scavenging Activity of YPG 

In addition to the qualitative analysis of YPG, the contents of six main components in YPG, namely, chlorogenic acid, puerarin, 3′-methoxypuerarin, polydatin, glycyrrhizic acid, and emodin, were also determined with the help of the HPLC-DAD method. The method validation results in Table 2 reveal that all analytes showed good linear regression in the range of 0.01–2.00 mg/mL (R^2^ ≥ 0.9990). The limits of detection (LODs) were 0.38–2.13 μg/mL, and the limits of quantitation (LOQs) ranged between 1.14 and 6.47 μg/mL. The repeatability RSD was 1.18–2.94%, and the intermediate precision was 2.00–3.89%, indicating that the method has good precision. The RSD of stability was less than 3.60%, indicating that the six components were stable under storage conditions. The method also showed satisfactory accuracy, with recovery values ranging from 96.79% to 103.13%, and the RSD was less than 3.15% (data shown in Table S2 in Supporting Information).

**Figure 8:**
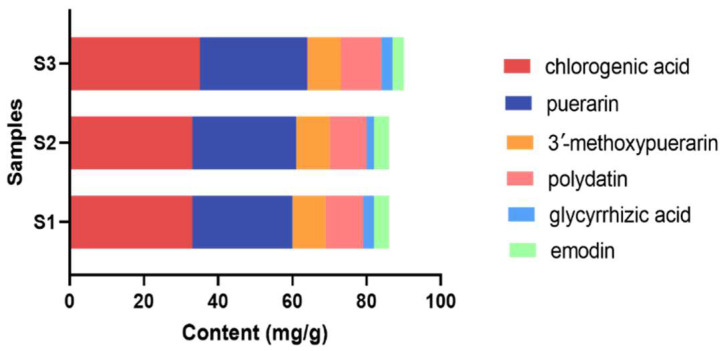
Quantitative analysis results of six main components in YPG.

As presented in Table 3 and Figure 8, chlorogenic acid (34.15 ± 1.25 mg/g) and puerarin (28.30 ± 1.09 mg/g) were found as the most abundant compounds in YPG, followed by 3′-methoxypuerarin (9.63 ± 0.12 mg/g), polydatin (10.83 ± 0.57 mg/g), glycyrrhizic acid (3.33 ± 0.56 mg/g), and emodin (4.14 ± 0.34 mg/g). The contents of the six compounds were stable in three batches of YPG samples, which suggested that the six compounds may be used as quality markers of YPG.

Considering that the six compounds are phenols with antioxidative and radical scavenging activities, total phenolic contents and DPPH scavenging capacities were also determined. The total phenolic contents of three batches of YPG were calculated as 144.66 ± 2.21 mg/g with the Folin–Ciocalteu method (Table 3). In addition, the results of an online HPLC-based DPPH radical quenching assay revealed that all six main components were good radical scavengers with scavenging percentages ranging from 30.3% to 59.2% (Table 3 and Figure S1 in Supporting Information). Emodin exhibited a lower capacity against the DPPH radical compared with the other five compounds. 

## 3. Materials and Methods

### 3.1. Reagents and Materials

YPG (Batch No. 200404) was provided by Shaanxi Dongke Pharmaceutical Co., Ltd. (Xianyang, China). Chlorogenic acid (≥95%), gallic acid (≥95%), 2,2-diphenyl-1-picrylhydrazyl (≥97%), and sodium carbonate anhydrous (≥99.8%) were purchased from Shanghai Aladdin Biochemical Technology Co., Ltd. (Shanghai, China); 3′-methoxypuerarin (≥98%) and polydatin (≥98%) were purchased from Chengdu Efa Biotechnology Co., Ltd. (Chengdu, China); puerarin (≥98%) and Folin–Ciocalteu reagent (>99.5%) were purchased from Shanghai Yien Chemical Technology Co., Ltd. (Shanghai, China); glycyrrhizic acid (≥98%) was purchased from Dalian Meilun Biotechnology Co., Ltd. (Dalian, China); emodin (≥99%) was obtained from China Institute for Food and Drug Control (Beijing, China). LC-MS-grade methanol, acetonitrile, and formic acid for HPLC analysis and LC-MS analysis were purchased from Tedia Co. (Fairfield, OH, USA). The water used was obtained from a Milli-Q water purification system (Bedford, MA, USA).

### 3.2. Sample Preparation

First, 100.00 mg of YPG was accurately weighed and added to 50% methanol (*v*/*v*, 1 mL). After extraction in an ultrasonic bath (300 W, 40 kHz, 50 °C) for 30 min, the extracted solution was centrifuged (13,000 rpm, 10min) and filtered through a 0.22 μm membrane. The obtained solution was kept at 4 °C until analysis.

### 3.3. Preparation of Standard Solutions

Six reference standards (chlorogenic acid 20.00 mg, puerarin 16.00 mg, 3′-methoxypuerarin 15.00 mg, polydatin 10.00 mg, glycyrrhizic acid 10.00 mg, emodin 5.00 mg) were accurately weighed and dissolved in 10 mL of 50% (*v*/*v*) methanol to prepare the respective stock solutions. The stock solutions were mixed and diluted with 50% methanol to prepare a series of mixed reference solutions in certain concentrations. All standard solutions were prepared in a 10 mL dark-brown volumetric flask and stored in a refrigerator at 4 °C before use.

### 3.4. Qualitative Analysis with HPLC–Q-Exactive MS

HPLC analysis was performed on a Thermo U3000 HPLC system (Thermo, San Jose, CA, USA). A CAPCELL PAK C18 MG II (150 mm × 4.6 mm, 3 μm; Osaka Soda, Osaka, Japan) column was used. The mobile phase consisted of 0.5% formic acid (A) and acetonitrile (B), with the elution gradient as follows: 0–10 min, 5–20% B; 10–30 min, 20–35% B; 30–32 min, 35–55% B; 32–35 min, 55–95% B; 35–42 min, 95% B, flow rate 0.4 mL/min. The column temperature was maintained at 35 °C, and the injection volume was 5 μL. 

The Q-Exactive mass spectrometer (Thermo, San Jose, USA) equipped with a heated electrospray ion source (HESI) was operated in both positive and negative modes. The MS parameters were set as follows: scanning mode, Full MS/ddMS^2^; nebulizer voltage, 2.5 kV; sheath gas, 50 arb; aux gas, 14 arb; capillary temperature 320 °C; probe heater temperature, 300 °C; scanning range, *m/z* 100–1500. For different compounds, the collision energy was 30 and 40 eV. Instrument control and data acquisition were achieved using Xcalibur 2.3.1 (Thermo, San Jose, USA).

The raw data were processed with Compound Discoverer 3.1 (CD, Thermo, San Jose, USA) using a self-built compound library and workflow. The compound information was collected from public databases, including PubChem (https://pubchem.ncbi.nlm.nih.gov/), CAS SciFinder (https://scifindern.cas.org/), and CNKI (https://www.cnki.net/). Then, the original data of MS detection were matched with the self-built database in Compound Discoverer 3.1. The workflow included peak extraction, normalization, and compound annotation, and the specific parameters in the process were as follows: retention time range, 0–42 min; mass range, 100–1500 Da; positive adducts, [M + H]^+^, [2M + H]^+^; negative adducts, [M − H]^−^, [2M − H]^−^, [M − 2H]^2−^; mass tolerance, 5.0 ppm; S/N threshold, 3; minimum peak intensity, 100000.

### 3.5. Quantitative Analysis by HPLC-DAD

For quantitative analysis, an Agilent 1260 system equipped with a G7112C Quat Pump, a G7129A Vial sampler, and a G7117C diode array detector (DAD) was used (Agilent, Santa Clara, CA, USA). The chromatographic parameters were the same as described in Section 3.4. The chromatograms were recorded at 254 nm.

### 3.6. Quantitative Analysis Method Validation

In order to verify the applicability of the established method, the linearity, limits of quantitation (LOQs), limits of detection (LODs), repeatability, precision, stability, and recovery were validated. In the linear relationship experiment, the standard solution with a certain concentration gradient was used to draw the curve of the peak area (y) of the standard and the corresponding concentration (x, mg/mL). In the precision test, the standard solution was analyzed 6 times continuously, the intra-day precision was analyzed, and the intermediate precision was analyzed by different analysts at different times. The sample solution was analyzed at 0, 2, 4, 8, and 12 h in the stability test. In the repeatability test, the contents of the six target compounds in the sample solution were quantified. At the same time, the standard substance of each analyte was added to the sample according to 80%, 100%, and 120% of its content in the sample solution to prepare the sample solution for the recovery test. Three replicates were required for each spiked amount, and the recovery rate was calculated as described previously [16].

### 3.7. Determination of Total Phenolic Contents

#### 3.7.1. Total Phenol Standard Curve Drawing

The total phenolic contents were measured according to a previously reported Folin–Ciocalteu reagent-based method, with gallic acid as a reference substance [23,24]. The absorbance was measured at 760 nm with an ultraviolet spectrophotometer. With absorbance as y, and total phenol mass concentration as x, the standard curve was drawn. The linear regression equation of gallic acid was y = 0.0965x + 0.0277 (R^2^ = 0.9992), and the linear range was 0.01–0.50 mg/mL.

#### 3.7.2. Total Phenol Content in the Sample

Different batches of YPG samples were taken, and the solution was prepared according to the above method. The determination was repeated three times, and the content of each sample was calculated according to the linear regression equation.

### 3.8. In Vitro Antioxidant Activity Evaluation—DPPH Radical Scavenging Activity

The DPPH free radical scavenging activities of the six components in YPG were evaluated with an HPLC-based method, as previously reported [14]. The scavenging percentages were calculated using the following equation:

Scavenging percentage (%) = (A_original_ − A_DPPH_)/A_original_ × 100%

where A_original_ stands for the absolute HPLC peak area of each ingredient, and A_DPPH_ stands for the HPLC peak area of each ingredient after YPG reacted with DPPH.

## 4. Conclusions

In this study, a set of integrated methods, which include the qualitative HPLC–Q-Exactive MS method and the quantitative HPLC-DAD method, were established to profile the chemical properties of YPG from a macroscopic and systematic view. In the qualitative analysis part, 380 components were tentatively identified in YPG with the help of a self-built compound database and Compound Discoverer software. In the quantitative analysis part, the contents of six main components in YPG were determined with a validated method. These results may provide clear chemical information for the quality control and pharmacological research of YPG. This study may also contribute as a valuable reference for research on other complex TCM compounded prescriptions.

## Figures and Tables

**Table 1:** Mass spectrometry information of chemical components in YPG.

No.	Name	RT (min)	Formula	Ion Type	Molecular Ion (*m/z*)	Main Product Ion (*m/z*)
1	ephedrannin A	3.40	C_30_H_20_O_11_	[M + H]^+^	557.1104	395.1281, 215.0650, 177.0543, 145.0284
2	glucose	3.63	C_6_H_12_O_6_	[M + H]^+^	181.0705	163.0792, 144.0655, 109.0286, 81.0340
				[M − H]^−^	179.0551	161.0445, 141.0181, 117.0181, 87.0073
3	secologanic acid	3.67	C_16_H_22_O_10_	[M − H]^−^	373.1134	347.9473, 189.0156, 161.0234, 135.0440
4	*D*-mannitol	3.82	C_6_H_14_O_6_	[M + H]^+^	183.0862	147.0650, 129.0543, 104.1073, 69.0342
5	sucrose	3.86	C_12_H_22_O_11_	[M + H]^+^	343.1227	306.1183, 145.0495, 127.0390, 85.0290
				[M − H]^−^	341.1083	261.7968, 179.0559, 113.0230, 59.0125
6	allantoin	3.99	C_4_H_6_N_4_O_3_	[M + H]^+^	159.0512	142.0862, 114.0915, 99.0193, 70.0658
* 7	quinic acid	4.13	C_7_H_12_O_6_	[M + H]^+^	193.0706	157.0492, 147.0652, 129.0546, 111.0443
8	salicylic acid	4.14	C_7_H_6_O_3_	[M + H]^+^	139.0389	122.0714, 111.0443, 97.0287, 85.0289
9	4-aminophenol	5.72	C_6_H_7_NO	[M + H]^+^	110.0604	87.0046, 81.0340, 78.9949
* 10	citric acid	5.59	C_6_H_8_O_7_	[M + H]^+^	193.0343	161.0595, 151.0388, 133.0647, 105.0702
				[M − H]^−^	191.0188	173.0085, 129.0180, 111.0075, 87.0071
11	guanosine	6.34	C_10_H_13_N_5_O_5_	[M + H]^+^	284.0986	258.4957, 152.0566, 135.0302, 110.0351
				[M − H]^−^	282.0843	169.3754, 150.0411, 88.1632, 61.9871
12	adenosine	6.46	C_10_H_13_N_5_O_4_	[M + H]^+^	268.1037	213.3238, 169.7115, 136.0617, 85.0288
* 13	gallic acid	7.23	C_7_H_6_O_5_	[M + H]^+^	171.0291	154.0974, 130.0863, 115.0392, 70.0658
14	coumalic acid	7.40	C_6_H_4_O_4_	[M + H]^+^	141.0184	113.9639, 90.9481, 72.9378, 56.9430
15	tachioside	8.05	C_13_H_18_O_8_	[M − H]^−^	301.0926	283.1918, 257.0457, 221.1909, 151.0029
16	isotachioside	8.27	C_13_H_18_O_8_	[M − H]^−^	301.0926	284.0321, 243.0658, 178.9978, 151.0026
17	ephedroxane	8.27	C_11_H_13_NO_2_	[M + H]^+^	192.1019	164.9844, 146.9612, 106.0654, 87.0445
18	quinaldic acid	8.27	C_10_H_7_NO_2_	[M + H]^+^	174.0551	146.9612, 128.9507, 105.9351, 55.9352
* 19	hordenine	8.56	C_10_H_15_NO	[M + H]^+^	166.1225	151.0101, 121.0649, 103.0546, 93.0703
20	leonuriside A	8.99	C_14_H_20_O_9_	[M − H]^−^	331.1035	285.0384, 253.0501, 169.0130, 125.0231
21	4-vinylguaiacol	9.19	C_9_H_10_O_2_	[M + H]^+^	151.0755	133.0761, 123.9456, 119.0493, 91.0547
22	7α-morroniside	9.50	C_17_H_26_O_11_	[M − H]^−^	405.1401	371.0939, 243.0672, 191.0199, 111.0070
23	tetramethylpyrazine	9.83	C_8_H_12_N_2_	[M + H]^+^	137.1075	111.0080, 93.0704, 68.9978
24	shuangkangsu	10.49	C_20_H_30_O_14_	[M − H]^−^	493.1560	447.2225, 431.0965, 269.0450, 169.0130
25	*epi*-gallocatechin	10.69	C_15_H_14_O_7_	[M + H]^+^	307.0807	289.1790, 243.1704, 208.9966, 139.0389
				[M − H]^−^	305.0665	247.5994, 219.0664, 165.0182, 125.0232
26	robinin	10.73	C_33_H_40_O_19_	[M + H]^+^	741.2226	678.4389, 579.1688, 381.0964, 297.0752
27	5-(hydroxymethyl)furfural	10.85	C_6_H_6_O_3_	[M + H]^+^	127.0393	111.9689, 110.0238, 84.9603, 55.9352
28	*N*-ethylbenzylamine	10.90	C_8_H_11_N	[M + H]^+^	122.0967	107.0732, 105.0336, 95.0495, 88.0237
29	vicenin-2	11.00	C_27_H_30_O_15_	[M + H]^+^	595.1641	433.1127, 415.1019, 313.0700, 283.0596
				[M − H]^−^	593.1510	539.2719, 521.2607, 463.2737, 226.9863
30	4′-hydroxyacetophenone	11.09	C_8_H_8_O_2_	[M + H]^+^	137.0599	122.0364, 116.9720, 95.0497, 55.9352
31	6-hydroxykynurenic acid	11.35	C_10_H_7_NO_4_	[M + H]^+^	206.0445	178.0497, 148.1121, 117.0699, 90.0797
				[M − H]^−^	204.0293	168.1795, 160.0393, 132.0446, 110.1680
32	chlorogenic acid butyl ester	11.36	C_20_H_26_O_9_	[M − H]^−^	409.1495	365.0681, 337.0357, 241.0023, 169.0135
33	mirificin-4′-*O*-glucoside	11.73	C_32_H_38_O_18_	[M + H]^+^	711.2118	579.1680, 417.1183, 399.1065, 297.0753
				[M − H]^−^	709.1986	487.1239, 457.1142, 294.0533, 266.0583
34	mandelonitrile	11.82	C_8_H_7_NO	[M + H]^+^	134.0600	106.0654, 91.0544, 79.0548
35	kakkalide	11.83	C_28_H_32_O_15_	[M + H]^+^	609.1803	447.1279, 411.1072, 327.0857, 297.0755
				[M − H]^−^	607.1663	588.1254, 487.1243, 309.0403, 281.0458
36	mahuannin A	11.87	C_30_H_24_O_10_	[M − H]^−^	543.1324	528.5940, 497.2623, 381.1213, 265.0987
37	2,6-dihydroxybenzoic acid	11.92	C_17_H_24_O_9_	[M + H]^+^	373.1480	308.2842, 237.3755, 151.0375, 107.0485
38	8-*epi*-loganic acid	11.96	C_16_H_24_O_10_	[M + H]^+^	377.1438	357.1662, 339.1559, 265.0587, 237.0277
				[M − H]^−^	375.1292	315.8566, 265.2063, 201.0163, 113.0231
39	swertiamarin	12.02	C_16_H_22_O_10_	[M − H]^−^	373.1134	357.0130, 295.0618, 201.0158, 135.0433
40	8-*epi*-loganin	12.30	C_17_H_26_O_10_	[M − H]^−^	389.1448	371.8339, 345.1187, 227.0693, 185.0593
41	5-methoxysalicylic acid	12.31	C_8_H_8_O_4_	[M + H]^+^	169.0498	151.0391, 128.9508, 111.0444, 93.0339
42	norephedrine	12.65	C_9_H_13_NO	[M + H]^+^	152.1068	134.0964, 117.0700, 115.0545, 91.0547
43	7-*epi*-vogeloside	12.81	C_17_H_24_O_10_	[M − H]^−^	387.1295	341.1095, 272.9591, 227.0690, 179.0566
44	loganic acid	12.88	C_16_H_24_O_10_	[M − H]^−^	375.1292	287.1191, 201.0159, 189.0158, 135.0440
* 45	protocatechuic acid	12.90	C_7_H_6_O_4_	[M + H]^+^	155.0339	137.0233, 117.0701, 107.0495, 72.9379
46	polygalin B	13.12	C_28_H_32_O_15_	[M + H]^+^	609.1803	555.7846, 447.1268, 285.0752, 270.0516
				[M − H]^−^	607.1663	460.8990, 325.0714, 310.0492, 282.0534
47	lonijaposide B	13.15	C_25_H_32_NO_12_	[M − H]^−^	537.1833	511.3804, 375.0705, 335.0791, 201.0155
48	3′-hydroxypuerarin	13.17	C_21_H_20_O_10_	[M − H]^−^	431.0975	415.0348, 311.0557, 283.0609, 255.0659
49	norpseudoephedrine	13.19	C_9_H_13_NO	[M + H]^+^	152.1068	134.0964, 117.0700, 106.0655
50	loganin	13.30	C_17_H_26_O_10_	[M − H]^−^	389.1448	371.8339, 326.0798, 227.0693, 185.0593
51	secologanoside	13.43	C_16_H_22_O_11_	[M + H]^+^	391.1222	239.0796, 241.0385, 163.0388, 151.0389
				[M − H]^−^	389.1083	280.5215, 194.8876, 121.0647, 95.0489
52	leucodelphidin	13.74	C_15_H_14_O_8_	[M − H]^−^	321.0607	305.2140, 265.0519, 253.0507, 186.9385
53	secologanin	13.83	C_17_H_24_O_10_	[M − H]^−^	387.1295	/
54	glucoisoliquiritin	14.04	C_27_H_32_O_14_	[M − H]^−^	579.1715	529.4413, 491.9212, 463.1199, 255.0662
55	glucoliquiritin apioside	14.07	C_32_H_40_O_18_	[M − H]^−^	711.2134	678.4865, 549.119, 457.1141, 255.0664
* 56	chlorogenic acid	14.15	C_16_H_18_O_9_	[M + H]^+^	355.1014	163.0388, 135.0440
				[M − H]^−^	353.0871	191.0553, 161.0234, 135.0440, 127.0385
* 57	neochlorogenic acid	14.22	C_16_H_18_O_9_	[M + H]^+^	355.1014	338.1603, 289.0706, 235.0594, 163.0390
* 58	catechin	14.25	C_15_H_14_O_6_	[M + H]^+^	291.0857	255.7885, 107.0648, 139.0389, 123.0442
				[M − H]^−^	289.0714	245.0817, 203.0705, 151.0387.123.0439
59	ephedrine	14.36	C_10_H_15_NO	[M + H]^+^	166.1225	148.1119, 133.0886, 117.0700, 91.0548
* 60	cryptochlorogenic acid	14.37	C_16_H_18_O_9_	[M + H]^+^	355.1014	337.0915, 235.0589, 205.0494, 163.0388
61	pseudoephedrine	14.43	C_10_H_15_NO	[M + H]^+^	166.1225	148.1119, 133.0887, 117.0701, 91.0546
62	ephedrannin D4	14.44	C_30_H_24_O_14_	[M − H]^−^	607.1089	563.1151, 487.1202, 413.0903, 267.0680
63	puerarin 6″-*O*-xyloside	14.61	C_26_H_28_O_13_	[M + H]^+^	549.1590	417.1175, 381.0964, 297.0753, 267.0648
				[M − H]^−^	547.1447	437.0846, 295.0609, 277.0504, 267.0661
64	chrysoeriol 7-*O*-neohesperidoside	14.65	C_28_H_32_O_15_	[M + H]^+^	609.1803	447.1284, 429.1182, 327.0859, 285.0755
				[M − H]^−^	607.1663	547.1422, 487.1246, 295.0607, 267.0660
* 65	*p*-hydroxybenzoic acid	14.70	C_7_H_6_O_3_	[M + H]^+^	139.0389	121.0286, 111.0443, 93.0339
* 66	amygdalin	14.72	C_20_H_27_NO_11_	[M + H]^+^	458.1647	355.1036, 213.0755, 163.0389, 107.0495
				[M − H]^−^	456.1505	382.6093, 323.0968, 256.1356, 161.0449
67	methyl caffeate	14.83	C_10_H_10_O_4_	[M + H]^+^	195.0652	177.0544, 163.0388, 145.0283, 117.0336
68	3,4-dimethyl-5-phenyloxazolidine	14.94	C_11_H_15_NO	[M + H]^+^	178.1227	162.1274, 147.1040, 117.0700, 105.0702
69	apigenin 5-rhamnoside	15.01	C_21_H_20_O_9_	[M + H]^+^	417.1167	381.0964, 321.0743, 297.0753, 267.0647
				[M − H]^−^	415.1027	295.0608, 267.0661, 253.0509, 223.0762
* 70	puerarin	15.02	C_21_H_20_O_9_	[M + H]^+^	417.1167	399.1072, 381.0956, 363.0844, 255.0646
				[M − H]^−^	415.1035	295.0608, 277.0507, 267.0661
71	mirificin	15.04	C_26_H_28_O_13_	[M + H]^+^	549.1590	417.1168, 399.1069, 297.0754, 267.0648
				[M − H]^−^	547.1447	418.4808, 295.0609, 267.0660, 114.2369
72	tectorigenin 7-*O*-xylosylglucoside	15.07	C_27_H_30_O_15_	[M + H]^+^	595.1641	379.0811, 325.0695, 216.0653, 121.0283
				[M − H]^−^	593.1510	495.0386, 473.1082, 310.0499, 282.0529
73	methylephedrine	15.14	C_11_H_17_NO	[M + H]^+^	180.1382	162.1275, 148.1076, 135.0804
74	methylpseudoephedrine	15.18	C_11_H_17_NO	[M + H]^+^	180.1382	162.1275, 147.1042, 135.0803, 117.0700
75	7-*O*-ethylsweroside	15.26	C_18_H_26_O_10_	[M − H]^−^	401.1451	325.7480, 269.1024, 253.0505, 178.0263
76	isoviolanthin	15.47	C_27_H_30_O_14_	[M + H]^+^	579.1692	417.1190, 399.1072, 297.0753, 267.0649
				[M − H]^−^	577.1554	531.2839, 518.0385, 283.0610, 268.0376
* 77	3′-methoxypuerarin	15.53	C_22_H_22_O_10_	[M + H]^+^	447.1277	285.0751, 270.0516, 225.0542, 137.0232
				[M − H]^−^	445.1134	430.0887, 367.1027, 327.1080, 215.0089
* 78	glycitin	15.54	C_22_H_22_O_10_	[M + H]^+^	447.1277	429.1190, 411.1062, 327.0855, 297.0754
				[M − H]^−^	445.1135	379.8243, 325.0714, 282.0530, 254.0597
79	benzyl alcohol	15.67	C_7_H_8_O	[M + H]^+^	109.0653	94.0148, 91.0546, 87.0045, 81.0704
80	methyl,4-hydroxycinnamate	15.68	C_10_H_10_O_3_	[M + H]^+^	179.0705	162.1276, 147.1041, 117.0700, 109.0651
81	*γ*-octalactone	15.94	C_8_H_14_O_2_	[M + H]^+^	143.1068	128.9508, 116.9721, 113.9639, 84.9603
82	*(6S-9R)*-roseoside	15.98	C_19_H_30_O_8_	[M + H]^+^	387.2004	369.1341, 297.0762, 267.0641, 151.0388
83	syringin	15.98	C_17_H_24_O_9_	[M + H]^+^	373.1480	308.0842, 292.1605, 237.3755, 151.0375
84	5,7-dihydroxyisobenzofuran	15.99	C_8_H_6_O_4_	[M + H]^+^	167.0339	148.1120, 133.0885, 117.0700, 111.0442
85	kingiside	16.01	C_17_H_24_O_11_	[M − H]^−^	403.1240	371.1025, 310.7592, 243.0664, 174.8555
* 86	puerarin-7-*O*-glucoside	16.04	C_27_H_30_O_14_	[M + H]^+^	579.1692	561.1633, 399.1074, 297.0753, 267.0647
87	sweroside	16.09	C_16_H_22_O_9_	[M + H]^+^	359.1333	297.8041, 265.6036, 197.0808, 127.0391
* 88	caffeic acid	16.29	C_9_H_8_O_4_	[M + H]^+^	181.0494	163.0388, 145.0284, 135.0441, 117.0337
				[M − H]^−^	179.0341	164.0098, 135.0440, 112.1822, 107.0492
89	ephedrannin D1	16.53	C_30_H_24_O_13_	[M + H]^+^	593.1279	576.3641, 447.1271, 327.0849, 297.0745
90	5-*p*-coumaroylquinic acid	16.70	C_16_H_18_O_8_	[M + H]^+^	339.1071	266.4322, 245.8672, 147.0439, 119.0494
				[M − H]^−^	337.0930	191.0553, 163.0390, 119.0470, 93.0332
91	loniceracetalide A	16.71	C_21_H_32_O_11_	[M − H]^−^	459.1869	/
92	piceatannol 3′-*O*-glucoside	16.77	C_20_H_22_O_9_	[M − H]^−^	405.1190	359.0753, 243.0659, 201.0549, 159.0440
93	lonijaposide D	16.82	C_26_H_32_NO_13_	[M − H]^−^	565.1774	550.4224, 519.2438, 445.1137, 325.0718
94	ephedralone	16.90	C_11_H_9_NO_4_	[M + H]^+^	220.0602	192.0652, 164.0699, 151.4024, 119.0490
				[M − H]^−^	218.0452	174.0550, 159.0315, 144.0077, 131.0365
95	neochlorogenic acid methyl ester	17.85	C_17_H_20_O_9_	[M + H]^+^	369.1173	191.9904, 177.0544, 145.0283, 117.0336
				[M − H]^−^	367.1029	255.0194, 191.0552, 173.0447, 134.0361
96	isoschaftoside	17.86	C_26_H_28_O_14_	[M + H]^+^	565.1542	479.5669, 415.1016, 313.0699, 283.0596
				[M − H]^−^	563.1340	341.0680, 311.0559, 283.0609, 149.0235
97	2,6-dihydroxyphenylacetic acid	18.07	C_8_H_8_O_4_	[M + H]^+^	169.0498	151.0389, 146.9612, 128.9508, 123.0441
98	schaftoside	18.35	C_26_H_28_O_14_	[M + H]^+^	565.1542	520.6823, 433.1122, 313.0700, 283.0597
				[M − H]^−^	563.1340	529.1846, 341.0670, 311.0558, 283.0610
* 99	vanillic acid	18.50	C_8_H_8_O_4_	[M + H]^+^	169.0498	151.0388, 146.9612, 128.9508, 123.0442
100	flavoyadorinin B	18.51	C_23_H_24_O_11_	[M + H]^+^	477.1379	327.1659, 279.0375, 204.5438, 145.0499
101	ephedrannin D2	18.54	C_30_H_24_O_13_	[M − H]^−^	591.1150	547.1453, 462.7491, 285.0408, 253.0507
* 102	cinnamic acid	18.59	C_9_H_8_O_2_	[M + H]^+^	149.0597	133.0885, 121.0648, 103.0548, 95.0497
103	hydroxyphenylacetic acid	18.65	C_8_H_8_O_3_	[M + H]^+^	153.0546	135.1167, 112.0395, 90.9481, 72.9378
104	3-*O*-caffeoylshikimic acid	18.81	C_16_H_16_O_8_	[M + H]^+^	337.0910	181.0494, 163.0388, 145.0283, 95.0495
				[M − H]^−^	335.0771	269.1091, 179.0345, 161.0233, 133.0282
105	leucopelargonidin	18.82	C_15_H_14_O_6_	[M + H]^+^	291.0857	273.0753, 207.0647, 147.0440, 139.0389
				[M − H]^−^	289.0714	245.0818, 203.0705, 151.0392, 123.0441
106	licuraside	19.43	C_26_H_30_O_13_	[M − H]^−^	549.1609	502.1001, 429.1062, 255.0660, 119.0490
107	genistein 7-*O*-glucoside	20.25	C_21_H_20_O_10_	[M + H]^+^	433.1120	271.0595, 215.0700, 137.0231
* 108	liquiritin apioside	20.29	C_26_H_30_O_13_	[M − H]^−^	549.1609	482.5059, 297.0778, 255.0660, 135.0076
109	isoliquiritin apioside	20.34	C_26_H_30_O_13_	[M − H]^−^	549.1609	488.6683, 429.1148, 255.0659, 135.0076
* 110	liquiritin	20.38	C_21_H_22_O_9_	[M − H]^−^	417.1186	402.1664, 373.0210, 255.0662, 119.0490
111	secologanin dimethyl acetal	20.39	C_19_H_30_O_11_	[M − H]^−^	433.1706	/
112	3-methoxyphenol	20.50	C_7_H_8_O_2_	[M + H]^+^	125.0599	102.9706, 97.0287, 84.9602
113	piceid gallate A	20.63	C_27_H_26_O_13_	[M − H]^−^	557.1293	/
* 114	polydatin	20.92	C_20_H_22_O_8_	[M − H]^−^	389.1236	227.0707, 185.0598, 159.0808, 143.0491
115	lonicerin	21.02	C_27_H_30_O_15_	[M + H]^+^	595.1641	433.1108, 313.0717, 271.0596, 215.0697
				[M − H]^−^	593.1510	430.4479, 329.5679, 285.0396, 227.0705
116	isoliquiritin	21.24	C_21_H_22_O_9_	[M − H]^−^	417.1186	255.0660, 153.0182, 135.0075, 119.0489
* 117	quercetin 3-glucoside	21.69	C_21_H_20_O_12_	[M − H]^−^	463.0880	300.0272, 271.0247, 255.0296, 151.0027
118	kaempferol 7-*O*-glucopyranoside	21.69	C_21_H_20_O_11_	[M + H]^+^	449.1072	330.0535, 287.0545, 203.4280, 153.0181
				[M − H]^−^	447.0931	410.9457, 325.0732, 285.0397, 256.0383
119	neoisoliquiritin	22.95	C_21_H_22_O_9_	[M − H]^−^	417.1186	374.0878, 255.0660, 153.0183, 135.0076
* 120	coumarin	23.54	C_9_H_6_O_2_	[M + H]^+^	147.0442	131.9743, 119.0493, 113.9640
* 121	daidzein	23.56	C_15_H_10_O_4_	[M − H]^−^	253.0502	224.0468, 209.0598, 197.0602, 135.0076
122	rhoifolin	23.76	C_27_H_30_O_14_	[M + H]^+^	579.1692	515.2410, 429.1205, 327.0858, 297.0754
* 123	isochlorogenic acid A	23.89	C_25_H_24_O_12_	[M − H]^−^	515.1185	353.0883, 335.0772, 173.0445, 135.0440
124	reynoutrin	24.65	C_20_H_18_O_11_	[M − H]^−^	433.0771	/
125	avicularin	25.43	C_20_H_18_O_11_	[M − H]^−^	433.0771	/
* 126	resveratroloside	25.46	C_20_H_22_O_8_	[M + H]^+^	391.1375	229.0856, 211.0759, 135.0440, 107.0495
127	liquiritigenin 7,4′-diglucoside	25.61	C_27_H_32_O_14_	[M + H]^+^	581.1848	538.0963, 431.0979, 311.0434, 287.0430
128	centauroside	25.64	C_34_H_46_O_19_	[M − H]^−^	757.2545	679.1150, 525.1623, 458.1185, 254.0573
129	3,4-dicaffeoylquinic acid	25.75	C_25_H_24_O_12_	[M − H]^−^	515.1185	437.3583, 353.0874, 191.0552, 135.0440
130	herniarin	26.18	C_10_H_8_O_3_	[M + H]^+^	177.0546	149.0597, 145.0283, 117.0336, 89.0390
131	catechin-5-*O-β-D*-glucopyranoside	26.54	C_21_H_24_O_11_	[M − H]^−^	451.1243	313.0739, 289.0719, 191.0340, 167.0340
132	vanillin	26.58	C_8_H_8_O_3_	[M + H]^+^	153.0546	131.9743, 125.0597, 111.0443, 93.0338
133	4,7-dihydroxyflavone 7-*D*-glucoside	26.84	C_21_H_20_O_9_	[M + H]^+^	417.1167	338.5892, 255.0647, 227.0695, 199.0747
134	methyl chlorogenate	27.00	C_17_H_20_O_9_	[M + H]^+^	369.1173	313.0666, 285.0745, 207.0644, 161.0596
135	ketologanin	27.07	C_17_H_24_O_10_	[M + H]^+^	389.1433	371.1681, 324.1584, 225.0426, 151.0388
136	naringin	27.96	C_27_H_32_O_14_	[M + H]^+^	581.1848	449.1047, 431.0979, 329.0610, 311.0434
137	*(E)*-aldosecologanin	28.10	C_34_H_46_O_19_	[M − H]^−^	757.2545	679.1150, 595.2075, 525.1623, 458.1185
138	dihydrocaffeic acid	28.25	C_9_H_10_O_4_	[M + H]^+^	183.0649	165.0545, 151.0389, 123.0441, 113.9639
139	*p*-coumaric acid	28.25	C_9_H_8_O_3_	[M + H]^+^	165.0544	137.0597, 133.0283, 109.0650, 79.0547
140	secoxyloganin	28.25	C_17_H_24_O_11_	[M + H]^+^	405.1379	373.2119, 309.2449, 165.0545, 151.0389
141	benzoic acid	28.26	C_7_H_6_O_2_	[M + H]^+^	123.0441	105.0450, 95.0495, 67.0549
142	1,5-dicaffeoylquinic acid	28.63	C_25_H_24_O_12_	[M − H]^−^	515.1185	454.9042, 353.0873, 191.0552, 173.0446
143	vogeloside	28.65	C_17_H_24_O_10_	[M + H]^+^	389.1433	233.2362, 195.0655, 151.0389, 107.0495
144	3-*O*-caffeoylquinic acid methyl ester	28.94	C_17_H_20_O_9_	[M + H]^+^	369.1173	207.0649, 177.0546, 148.0514, 107.0857
145	quercitrin	30.05	C_21_H_20_O_11_	[M + H]^+^	449.1072	330.0535, 287.0545, 269.0448, 153.0181
				[M − H]^−^	447.0931	403.1030, 241.0501, 197.0599, 174.9555
146	4-feruloylquinic acid	30.26	C_17_H_20_O_9_	[M + H]^+^	369.1173	239.4636, 207.0649, 177.0539, 148.0516
* 147	naringenin	31.32	C_15_H_12_O_5_	[M − H]^−^	271.0609	230.0589, 177.0189, 151.0026, 119.0490
148	kuzubutenolide A	31.41	C_23_H_24_O_10_	[M + H]^+^	461.1433	299.0909, 253.0853, 193.0497, 107.0494
149	pueroside A	31.42	C_29_H_34_O_14_	[M + H]^+^	607.2010	461.1439, 376.1363, 299.0908, 107.0494
150	epicatechingallate	31.75	C_22_H_18_O_10_	[M + H]^+^	443.0962	390.0869, 291.0855, 273.0755, 123.0441
151	garbanzol	31.76	C_15_H_12_O_5_	[M + H]^+^	273.0752	242.4491, 189.0543, 153.0180, 123.0441
152	chrysoeriol 7-*O*-glucopyranoside	31.80	C_22_H_22_O_11_	[M + H]^+^	463.1220	445.1107, 427.1008, 343.0803, 313.0704
153	sophoraside A	31.92	C_24_H_26_O_10_	[M + H]^+^	475.1587	313.1061, 267.1010, 253.0853, 107.0494
				[M − H]^−^	473.1447	377.9086, 311.0924, 267.1024, 252.0786
154	vitexin	32.25	C_21_H_20_O_10_	[M + H]^+^	433.1120	415.1018, 397.0909, 313.0698, 283.0597
				[M − H]^−^	431.0975	269.0453, 240.0423, 225.0551, 193.4129
155	5-*O*-coumaroylcaffeoylquinic acid	32.38	C_25_H_24_O_11_	[M + H]^+^	501.1379	483.1254, 320.0835, 255.0652, 163.0388
				[M − H]^−^	499.1244	431.0978, 291.0275, 269.0454, 240.0423
* 156	resveratrol	32.64	C_14_H_12_O_3_	[M + H]^+^	229.0856	211.0747, 183.0808, 135.0441, 107.0494
* 157	ferulic acid	32.90	C_10_H_10_O_4_	[M + H]^+^	195.0652	177.0544, 163.0389, 138.0661, 107.0494
				[M − H]^−^	193.0498	165.0005, 134.0361, 126.9024, 102.9472
* 158	isoferulic acid	32.91	C_10_H_10_O_4_	[M + H]^+^	195.0652	177.0544, 163.0388, 149.0596, 109.0287
				[M − H]^−^	193.0498	161.0233, 149.0236, 134.0363, 121.0281
159	kudzusaponin A1	34.62	C_52_H_84_O_23_	[M − H]^−^	1075.5314	1029.5265, 763.3842, 603.3890, 485.3619
160	hyperoside	35.15	C_21_H_20_O_12_	[M + H]^+^	465.1023	447.1085, 303.0492, 286.0449, 257.0425
161	polygalin A	35.16	C_23_H_24_O_11_	[M + H]^+^	477.1379	355.1165, 315.0853, 271.0960, 229.0856
				[M − H]^−^	475.1241	267.0660, 252.0424, 201.9968, 132.0607
162	7-hydroxy-4-methoxy-5-methylcoumarin	35.24	C_11_H_10_O_4_	[M + H]^+^	207.0650	189.0544, 161.0599, 150.0261, 123.0807
163	glycitin-6″-*O*-xylosyl	36.12	C_27_H_30_O_14_	[M + H]^+^	579.1692	433.1124, 337.0699, 313.0699, 283.0596
164	cuspidatumin A	36.14	C_14_H_12_O_4_	[M + H]^+^	245.0805	229.0854, 161.0122, 121.0286, 98.9757
				[M − H]^−^	243.0661	225.1119, 207.1026, 174.9554, 146.9600
165	3,5-dicaffeoylquinic acid methyl ester	36.23	C_26_H_26_O_12_	[M + H]^+^	531.1486	513.1385, 369.1514, 283.0595, 163.0388
* 166	rutin	36.27	C_27_H_30_O_16_	[M + H]^+^	611.1602	465.1010, 303.0493, 257.0441, 229.0495
* 167	taxifolin	36.27	C_15_H_12_O_7_	[M + H]^+^	305.0653	287.1236, 269.1125, 227.1023, 191.0814
168	pueroside B	36.31	C_30_H_36_O_15_	[M + H]^+^	637.2119	475.1591, 313.1064, 267.1011, 107.0494
169	pueroside C	36.31	C_24_H_26_O_10_	[M + H]^+^	475.1587	457.3117, 313.1034, 249.1549, 107.0486
170	macranthoidin B	36.35	C_65_H_106_O_32_	[M + H]^+^	1399.6711	1075.5695, 943.5251, 795.2725, 633.2202
				[M − H]^−^	1397.6552	1073.5525, 911.5010, 749.4481, 603.3898
171	kudzusaponin SA2	36.39	C_47_H_76_O_19_	[M + H]^+^	945.5027	848.4162, 763.4678, 679.2439, 421.3453
172	macranthoidin A	36.40	C_59_H_96_O_27_	[M + H]^+^	1237.6183	1076.5618, 943.5206, 751.4630, 603.2128
				[M − H]^−^	1235.6036	1189.5997, 1073.5534, 911.5006, 749.4482
173	kudzusaponin SA4	36.40	C_47_H_74_O_20_	[M + H]^+^	959.4806	892.2470, 764.6243, 615.3878, 421.3457
174	saponin 1	36.43	C_58_H_94_O_26_	[M + H]^+^	1207.6077	1075.5693, 913.5162, 751.4610, 603.2120
				[M − H]^−^	1205.5934	881.4901, 749.4479, 603.3898, 471.3479
175	24-hydroxy-licorice-saponin A3	36.45	C_48_H_72_O_22_	[M + H]^+^	1001.4559	825.4282, 763.0059, 631.3789, 469.3288
176	3,4-*O*-dicaffeoylquinic acid methyl ester	36.46	C_26_H_26_O_12_	[M + H]^+^	531.1486	319.0808, 271.0598, 177.0545, 163.0388
				[M − H]^−^	529.1344	443.6241, 367.1035, 191.0554, 135.1440
177	isoquercetin	36.47	C_21_H_20_O_12_	[M + H]^+^	465.1023	303.0494, 257.0439, 229.0495, 153.0182
178	soyasaponin A3	36.48	C_48_H_78_O_19_	[M + H]^+^	959.5183	813.4605, 439.3565, 141.0181, 85.0289
				[M − H]^−^	957.5065	911.5010, 749.4482, 587.3950, 471.3475
179	dipsacoside B	36.49	C_53_H_86_O_22_	[M − H]^−^	1073.5519	912.0020, 749.4480, 585.3804, 471.3478
180	kudzusaponin B1	36.52	C_48_H_76_O_21_	[M + H]^+^	989.4921	843.4330, 681.3870, 469.3314, 141.0181
				[M − H]^−^	987.4794	926.4868, 763.7924, 661.3583, 503.3387
181	saponin 4	36.55	C_58_H_94_O_27_	[M − 2H]^2−^	610.2908	/
182	licoricesaponin A3	36.57	C_48_H_72_O_21_	[M + H]^+^	985.4612	809.4323, 615.3887, 453.3356, 189.1634
				[M − H]^−^	983.4483	943.1790, 821.3969, 645.3637, 351.0566
183	neoliquiritin	36.58	C_21_H_22_O_9_	[M + H]^+^	419.1326	315.0854, 257.0803, 217.0483, 124.0392
184	6″-*O*-malonyldaidzin	36.58	C_24_H_22_O_12_	[M + H]^+^	503.1170	480.9303, 392.3837, 255.0647, 199.0751
185	*(2E)*-1-(2,3-dihydroxy-4-methoxyphenyl)-3-(4-hydroxyphenyl)-2-propen^-^-one	36.58	C_16_H_14_O_5_	[M + H]^+^	287.0908	245.0804, 207.0649, 193.0492, 121.0285
				[M − H]^−^	285.0765	270.0532, 177.0185, 150.0311, 108.0206
186	loniceroside D	36.59	C_53_H_86_O_23_	[M + H]^+^	1091.5607	1033.7538, 945.5055, 783.4556, 421.3455
				[M − H]^−^	1089.5470	1071.5394, 943.4768, 882.4898, 763.4315
187	akebiasaponin D	36.60	C_47_H_76_O_18_	[M + H]^+^	929.5079	767.4589, 635.4064, 437.3408, 189.1637
188	kudzusaponin A2	36.61	C_42_H_68_O_16_	[M + H]^+^	829.4560	764.7684, 649.3955, 455.3521, 269.0806
				[M − H]^−^	827.4425	763.3452, 677.4987, 516.0891, 333.8636
189	isorhamnetin 3-*O*-glucopyranoside	36.62	C_22_H_22_O_12_	[M + H]^+^	479.1170	397.5868, 317.0649, 274.0458, 120.0809
* 190	astragalin	36.62	C_21_H_20_O_11_	[M + H]^+^	449.1072	409.0180, 346.9581, 287.0544, 252.9790
				[M − H]^−^	447.0931	316.5085, 284.0322, 255.0294, 227.0343
191	7,4′-dihydroxyflavone	36.63	C_15_H_10_O_4_	[M + H]^+^	255.0645	227.0696, 199.0752, 137.0234, 91.0546
				[M − H]^−^	253.0502	224.0470, 208.0522, 135.0076, 91.0174
192	isorhamentin 3-*O*-rutinoside	36.66	C_28_H_32_O_16_	[M + H]^+^	625.1743	479.1161, 317.0652, 302.0414, 85.0289
				[M − H]^−^	623.1613	527.7530, 415.1031, 252.0425, 223.0404
* 193	4′-methoxypuerarin	36.67	C_22_H_22_O_9_	[M + H]^+^	431.1326	395.1120, 365.1009, 311.0910, 271.0595
194	4,5-*O*-dicaffeoylquinic acid methyl ester	36.67	C_26_H_26_O_12_	[M − H]^−^	529.1344	483.1268, 463.2749, 367.1032, 253.0501
195	quercetin 3-*O*-arabinoside	36.67	C_20_H_18_O_11_	[M + H]^+^	435.0917	303.0501, 271.0596, 153.0180, 121.0280
196	loniceroside A	36.69	C_52_H_84_O_21_	[M − H]^−^	1043.5422	1025.5223, 763.3167, 709.8038, 532.3125
* 197	rhein	36.71	C_15_H_8_O_6_	[M + H]^+^	285.0392	269.0440, 257.0428, 151.0385, 121.0283
				[M − H]^−^	283.0246	268.0373, 217.0500, 175.0391, 133.0284
198	3,4,5-tricaffeoylquinic acid	36.72	C_34_H_30_O_15_	[M + H]^+^	679.1633	499.1226, 322.2479, 163.0387, 135.0440
				[M − H]^−^	677.1511	515.1179, 353.0875, 173.0446, 135.0440
199	choerospondin	36.74	C_21_H_22_O_10_	[M + H]^+^	435.1296	303.0501, 271.0596, 231.0647, 153.0180
200	4,5-dicaffeoylquinic acid	36.75	C_25_H_24_O_12_	[M + H]^+^	517.1326	499.1223, 453.8935, 269.0803, 163.0387
201	pollenitin B	36.76	C_22_H_22_O_12_	[M + H]^+^	479.1170	412.8673, 317.0651, 302.0415, 274.0472
202	medicarpin3-*O*-glucoside	36.77	C_22_H_24_O_9_	[M + H]^+^	433.1482	312.0939, 271.0596, 214.2812, 153.0182
203	lonfuranacid A	36.77	C_12_H_20_O_5_	[M + H]^+^	245.1377	229.0853, 189.1119, 125.0962, 97.1015
204	questin	36.78	C_16_H_12_O_5_	[M + H]^+^	285.0751	270.0518, 253.0490, 242.0574, 153.0179
205	Tricin 7-*O*-glucoside	36.78	C_23_H_24_O_12_	[M + H]^+^	493.1326	331.0807, 315.0493, 287.0537, 270.0518
206	tectoridin	36.79	C_22_H_22_O_11_	[M + H]^+^	463.1220	301.0700, 286.0467, 258.0517, 153.0181
* 207	liquiritigenin	36.80	C_15_H_12_O_4_	[M + H]^+^	257.0805	239.0705, 211.0756, 147.0439, 137.0232
				[M − H]^−^	255.0660	209.0605, 153.0183, 135.0077, 119.0490
208	subproside V	36.80	C_54_H_88_O_24_	[M − H]^−^	1119.5575	1073.5519, 911.5007, 749.4478, 603.3897
209	loniceroside E	36.81	C_53_H_86_O_21_	[M − H]^−^	1057.5570	1039.5623, 849.4960, 763.3219, 413.0908
210	kudzusaponin A5	36.82	C_48_H_78_O_20_	[M + H]^+^	975.5131	829.4545, 764.4423, 667.4020, 455.3510
211	torachrysone	36.82	C_14_H_14_O_4_	[M + H]^+^	247.0960	229.0856, 214.0621, 201.0907, 198.0673
				[M − H]^−^	245.0812	230.0579, 215.0343, 202.0625, 159.0440
212	macranthoside B	36.83	C_53_H_86_O_22_	[M + H]^+^	1075.5658	943.5235, 781.4703, 619.4197, 437.3409
213	glycyroside	36.83	C_27_H_30_O_13_	[M + H]^+^	563.1743	431.1331, 413.1223, 311.0907, 281.0803
				[M − H]^−^	561.1608	523.2799, 339.0867, 309.0767, 266.0582
214	ohyscion	36.84	C_16_H_12_O_5_	[M + H]^+^	285.0751	270.0518, 242.0567, 189.4096, 113.0597
				[M − H]^−^	283.0609	268.0375, 240.0419, 211.0391, 184.0518
215	afzelin	36.88	C_21_H_20_O_10_	[M + H]^+^	433.1120	418.8996, 271.0596, 243.0644, 215.0699
				[M − H]^−^	431.0975	269.0454, 240.0424, 225.0552, 152.9942
216	kudzusaponin SA3	36.89	C_53_H_86_O_23_	[M + H]^+^	1091.5607	929.5117, 767.4597, 635.4111, 437.3414
				[M − H]^−^	1089.5470	1043.5427, 881.4904, 749.4480, 603.3900
217	22β-acetoxyglycyrrhizin	36.91	C_44_H_64_O_18_	[M + H]^+^	881.4143	705.3826, 511.3415, 451.3196, 107.0859
218	kudzusaponin C1	36.93	C_54_H_88_O_23_	[M + H]^+^	1105.5767	959.5132, 797.498, 603.4246, 423.3591
219	questinol	36.94	C_16_H_12_O_6_	[M + H]^+^	301.0701	286.0460, 269.0439, 167.0338, 134.0362
220	3′-methoxydaidzin	36.96	C_22_H_22_O_10_	[M + H]^+^	447.1277	384.1155, 327.0859, 285.0752, 229.0857
221	loniceroside B	36.96	C_58_H_94_O_25_	[M + H]^+^	1191.6127	817.3367, 763.4253, 619.4137, 437.3397
222	citreorosein	36.97	C_15_H_10_O_6_	[M + H]^+^	287.0542	271.0596, 269.0443, 259.0960, 217.0491
				[M − H]^−^	285.0402	268.0367, 257.0461, 196.0532, 133.0284
223	herbacetin	37.01	C_15_H_10_O_7_	[M + H]^+^	303.0492	286.0430, 257.0442, 229.0497, 153.0181
				[M − H]^−^	301.0351	284.0315, 273.0407, 178.9976, 151.0026
224	betulonic acid	37.02	C_30_H_46_O_3_	[M + H]^+^	455.3507	409.3467, 388.4104, 203.1793, 189.1635
225	kudzusaponin A3	37.05	C_48_H_78_O_20_	[M + H]^+^	975.5131	829.4545, 667.4020, 455.3510, 141.0181
* 226	ononin	37.08	C_22_H_22_O_9_	[M + H]^+^	431.1326	269.0805, 254.0569, 213.0910, 107.0494
227	kaempferol 3-*O*-rutinoside	37.11	C_27_H_30_O_15_	[M + H]^+^	595.1641	525.0455, 433.1108, 287.0544, 271.0596
228	isobavachalcone	37.11	C_20_H_20_O_4_	[M + H]^+^	325.1429	309.0781, 285.0754, 189.0906, 95.0163
229	liqcoumarin	37.18	C_12_H_10_O_4_	[M + H]^+^	219.0648	201.0910, 174.0674, 133.1012, 105.0702
230	isokaempferide	37.19	C_16_H_12_O_6_	[M + H]^+^	301.0701	283.0596, 255.0636, 227.0698, 123.1169
231	uralsaponin F	37.21	C_44_H_64_O_19_	[M + H]^+^	897.4094	763.6343, 679.2714, 527.3329, 334.7203
232	daidzein 4′,7-diglucoside	37.25	C_27_H_30_O_14_	[M + H]^+^	579.1692	503.0054, 447.1283, 285.0753, 229.0858
233	liquoric acid	37.26	C_30_H_44_O_5_	[M + H]^+^	485.3245	323.1276, 255.0648, 199.0751, 163.0385
234	isoorientin	37.27	C_21_H_20_O_11_	[M + H]^+^	449.1072	330.0535, 287.0545, 153.0181, 135.0439
235	isorhodoptilometrin	37.31	C_17_H_14_O_6_	[M + H]^+^	315.0856	300.0623, 272.0670, 153.0185, 95.0858
				[M − H]^−^	313.0715	298.0479, 270.0529, 227.0343, 183.0454
236	chrysophanol	37.34	C_15_H_10_O_4_	[M + H]^+^	255.0645	237.0549, 227.0692, 199.0751, 187.0725
237	licoricesaponin G2	37.34	C_42_H_62_O_17_	[M + H]^+^	839.4040	663.3734, 487.3410, 469.3306, 141.0181
				[M − H]^−^	837.3906	763.7259, 724.4032, 351.0571, 193.0345
238	kudzusaponin SA1	37.35	C_42_H_68_O_15_	[M + H]^+^	813.4611	764.7317, 439.3548, 141.0181, 95.0860
				[M − H]^−^	811.4482	765.4406, 603.3923, 432.7037, 283.0584
239	1,4-dicaffeoylquinic acid	37.38	C_25_H_24_O_12_	[M + H]^+^	517.1326	460.9336, 414.0990, 269.0804, 213.0906
240	ethyl caffeate	37.38	C_11_H_12_O_4_	[M + H]^+^	209.0806	163.0388, 145.1011, 135.0441, 117.0337
				[M − H]^−^	207.0654	179.0341, 161.0233, 135.0441, 121.0284
241	apigenin 7-glucoside	37.41	C_21_H_20_O_10_	[M + H]^+^	433.1120	379.0797, 337.0701, 313.0699, 271.0596
				[M − H]^−^	431.0975	311.0562, 269.0453, 225.0554, 152.9949
* 242	daidzin	37.42	C_21_H_20_O_9_	[M + H]^+^	417.1167	387.2100, 297.0747, 255.0648, 199.0753
243	2,5-dimethyl-7-hydroxychromenone	37.46	C_11_H_10_O_3_	[M + H]^+^	191.0699	151.0387, 131.0856, 107.0861, 95.0860
244	prunetin	37.46	C_16_H_12_O_5_	[M + H]^+^	285.0751	253.0489, 242.0572, 211.0750, 151.0390
				[M − H]^−^	283.0609	268.0380, 240.0423, 197.0600, 168.0650
245	glabrolide	37.47	C_30_H_44_O_4_	[M + H]^+^	469.3298	233.1540, 175.1479, 135.1167, 107.0858
246	pinocembrin	37.53	C_15_H_12_O_4_	[M + H]^+^	257.0805	239.0701, 229.0850, 211.0752, 147.0440
247	polygonin B	37.54	C_26_H_26_O_13_	[M + H]^+^	547.1430	299.0909, 284.0674, 239.0702, 163.0385
* 248	glycyrrhizic acid	37.56	C_42_H_62_O_16_	[M + H]^+^	823.4086	647.3788, 471.3457, 453.3356
				[M − H]^−^	821.3959	763.8063, 469.3315, 351.0569, 193.0347
249	echinatin	37.56	C_16_H_14_O_4_	[M + H]^+^	271.0960	254.2115, 147.0438, 137.0596, 123.0441
				[M − H]^−^	269.0816	251.0708, 225.0552, 151.0030, 119.0490
250	hesperetin	37.62	C_16_H_14_O_6_	[M + H]^+^	303.0853	258.0517, 153.0183, 106.0866, 88.0762
				[M − H]^−^	301.0714	273.0774, 255.0296, 230.0580, 183.0447
251	*(S)*-naringenin	37.62	C_15_H_12_O_5_	[M + H]^+^	273.0752	189.0543, 153.0180, 123.0441
252	puerol B	37.64	C_18_H_16_O_5_	[M + H]^+^	313.1063	267.1011, 253.0854, 107.0495
				[M − H]^−^	311.0922	296.0689, 267.1026, 252.0789, 161.0233
253	coumestrol	37.72	C_15_H_8_O_5_	[M + H]^+^	269.0437	254.0572, 241.0492, 213.0543, 185.0595
				[M − H]^−^	267.0297	251.0709, 225.0550, 181.0649, 151.0026
254	polygonin A	37.73	C_25_H_24_O_13_	[M + H]^+^	533.1273	488.1885, 360.1438, 285.0753, 270.0517
				[M − H]^−^	531.1141	341.9318, 253.0502, 229.0135, 191.0555
255	tricin	37.73	C_17_H_14_O_7_	[M + H]^+^	331.0804	315.0492, 302.0406, 270.0519, 73.0291
				[M − H]^−^	329.0664	271.0247, 211.1332, 171.1017
256	neobavaisoflavone	37.76	C_20_H_18_O_4_	[M + H]^+^	323.1272	308.0663, 267.0647, 255.0648, 239.0698
257	biochanin	37.76	C_16_H_12_O_5_	[M + H]^+^	285.0751	270.0516, 253.0491, 225.0540, 137.0233
				[M − H]^−^	283.0609	268.0377, 240.0423, 224.0474, 135.0075
258	gancaonin V	37.76	C_19_H_20_O_4_	[M + H]^+^	313.1425	281.1162, 244.0359, 153.0181
				[M − H]^−^	311.1285	296.0687, 267.1025, 252.0789, 161.0232
259	6,7-dimethoxycoumarin	37.76	C_11_H_10_O_4_	[M + H]^+^	207.0650	189.1636, 175.0388, 148.0517, 91.0547
260	puerol A	37.78	C_17_H_14_O_5_	[M + H]^+^	299.0908	284.0674, 256.0726, 239.0698, 95.0163
				[M − H]^−^	297.0764	281.0457, 256.0376, 239.0346, 151.0025
261	biapigenin	37.78	C_30_H_18_O_10_	[M + H]^+^	539.0958	522.9716, 387.0856, 286.0465, 184.0731
				[M − H]^−^	537.0822	521.0623, 417.0622, 375.0506, 331.0608
262	2-methoxy-6-acetyl-7-methyljuglone	37.79	C_14_H_12_O_5_	[M + H]^+^	261.0754	243.0648, 215.0699, 200.0466, 187.0754
				[M − H]^−^	259.0608	243.1414, 231.0657, 216.0422, 188.0471
263	kudzusaponin SB1	37.79	C_53_H_86_O_22_	[M + H]^+^	1075.5658	943.5235, 751.4603, 437.3405, 189.1636
				[M − H]^−^	1073.5519	911.5006, 749.4489, 603.3903, 471.3480
264	diosmetin	37.81	C_16_H_12_O_6_	[M + H]^+^	301.0701	286.0468, 258.0523, 241.0491, 88.0762
				[M − H]^−^	299.0557	284.0325, 256.0372, 227.0344, 151.0030
265	*(-)-epi*afzelechin	37.82	C_15_H_14_O_5_	[M + H]^+^	275.0906	257.0795, 217.0492, 189.0544, 107.0495
				[M − H]^−^	273.0766	258.0532, 230.0579, 215.0343, 135.0076
266	licoricesaponin E2	37.85	C_42_H_60_O_16_	[M + H]^+^	821.3938	764.7926, 451.3198, 173.1327, 121.1012
267	methyl glycyrrhizate	37.86	C_43_H_64_O_16_	[M + H]^+^	837.4255	764.7705, 663.3716, 469.3308, 141.0181
268	licoisoflavanone	37.88	C_20_H_18_O_6_	[M + H]^+^	355.1169	337.1062, 299.0546, 179.0337, 123.0441
				[M − H]^−^	353.1027	335.0921, 312.0276, 217.0863, 189.0913
269	3-methoxyherbacetin	37.89	C_16_H_12_O_7_	[M + H]^+^	317.0647	302.0411, 237.0383, 153.0181, 127.0391
				[M − H]^−^	315.0506	300.0272, 272.0323, 188.0482, 112.9845
270	erybacin B	37.89	C_19_H_18_O_5_	[M + H]^+^	327.1222	271.0597, 117.0367, 95.0163, 77.0059
				[M − H]^−^	325.1076	309.2072, 297.0051, 197.1174, 171.1016
271	soyasaponin I	37.91	C_48_H_78_O_18_	[M + H]^+^	943.5241	797.4680, 764.6400, 423.3611, 85.0289
				[M − H]^−^	941.5101	912.5775, 763.8070, 615.3933, 438.3518
272	licoricesaponin B2	37.93	C_42_H_64_O_15_	[M + H]^+^	809.4296	633.3988, 439.3564, 285.2223, 107.0859
				[M − H]^−^	807.4168	763.8304, 520.9705, 351.0565, 193.0345
273	ephedrannin B	37.94	C_30_H_20_O_10_	[M + H]^+^	541.1106	415.0806, 389.1013, 171.0287, 153.0181
				[M − H]^−^	539.0981	521.2609, 507.2097, 396.8802, 266.9637
274	medicarpin	37.97	C_16_H_14_O_4_	[M + H]^+^	271.0960	253.0497, 229.0855, 197.0594, 121.0285
* 275	kaempferol	37.99	C_15_H_10_O_6_	[M + H]^+^	287.0542	271.0556, 254.0524, 226.0577, 153.0181
				[M − H]^−^	285.0402	268.0364, 257.0451, 241.0497, 211.0396
276	glyasperin D	38.04	C_22_H_26_O_5_	[M + H]^+^	371.1844	315.1218, 303.1219, 167.0701, 123.0441
277	isoliquiritigenin	38.09	C_15_H_12_O_4_	[M + H]^+^	257.0805	239.0698, 211.0755, 147.0440, 137.0232
				[M − H]^−^	255.0660	153.0182, 135.0077, 119.0489, 91.0175
278	3′-hydroxydaidzein	38.10	C_15_H_10_O_5_	[M + H]^+^	271.0594	253.0492, 243.0647, 215.0702, 153.0180
279	kaikasaponin III	38.15	C_48_H_78_O_17_	[M + H]^+^	927.5280	767.4596, 635.4124, 437.3406, 203.1794
280	3,4,3′,4′-tetrahydroxychalcone	38.16	C_15_H_12_O_5_	[M + H]^+^	273.0752	245.0811, 171.0285, 153.0181, 123.0442
281	araboglycyrrhizin	38.21	C_41_H_62_O_14_	[M + H]^+^	779.4183	/
282	macranthoside A	38.22	C_47_H_76_O_17_	[M + H]^+^	913.5130	781.4694, 617.4044, 423.3610, 141.0180
				[M − H]^−^	911.5004	749.4489, 603.3895, 471.3479, 423.3271
283	hydnocarpin	38.23	C_25_H_20_O_9_	[M + H]^+^	465.1173	447.1065, 286.0468, 257.0440, 147.0438
				[M − H]^−^	463.1033	447.2420, 285.0402, 255.0293, 208.9755
284	puerariafuran	38.24	C_16_H_12_O_5_	[M + H]^+^	285.0751	270.0512, 253.0493, 242.0569, 211.0754
285	vestitol	38.29	C_16_H_16_O_4_	[M + H]^+^	273.1118	255.1017, 227.1794, 137.0233, 121.0285
286	homobutein	38.31	C_16_H_14_O_5_	[M + H]^+^	287.0908	269.0440, 241.0491, 185.0592, 151.0389
287	glycycoumarin	38.34	C_21_H_20_O_6_	[M + H]^+^	369.1324	351.1228, 297.0746, 193.0494, 165.0545
288	licoricesaponin J2	38.34	C_42_H_64_O_16_	[M + H]^+^	825.4243	764.8267, 455.3507, 189.1634, 141.0181
				[M − H]^−^	823.4119	763.2627, 473.1696, 351.0565, 193.0342
289	licoricesaponin C2	38.35	C_42_H_62_O_15_	[M + H]^+^	807.4139	764.8302, 678.4443, 631.3784, 437.3406
				[M − H]^−^	805.4016	763.3167, 453.3408, 351.0559, 193.0349
290	3′-hydroxy-4′-*O*-methylglabridin	38.39	C_21_H_22_O_5_	[M − H]^−^	353.1393	/
291	blumenol A	38.39	C_13_H_20_O_3_	[M + H]^+^	225.1483	210.1245, 167.9932, 114.0913, 95.0860
292	dihydrodaidzein	38.40	C_15_H_12_O_4_	[M + H]^+^	257.0805	239.0690, 229.0851, 211.0744, 147.0439
* 293	formononetin	38.44	C_16_H_12_O_4_	[M + H]^+^	269.0803	254.0567, 213.0907, 118.0414, 95.0859
				[M − H]^−^	267.0660	252.0423, 225.0553, 195.0443, 132.0204
294	lupiwighteone	38.44	C_20_H_18_O_5_	[M + H]^+^	339.1219	322.2484, 283.0594, 271.0597, 209.1646
* 295	quercetin	38.48	C_15_H_10_O_7_	[M + H]^+^	303.0492	285.0393, 257.0439, 229.0493, 153.0181
				[M − H]^−^	301.0351	283.0246, 255.0298, 227.0342, 138.0312
296	glycyuralin E	38.52	C_21_H_22_O_6_	[M + H]^+^	371.1480	353.1372, 339.1213, 285.0749, 167.0695
				[M − H]^−^	369.1341	311.0558, 229.0865, 206.0213, 139.0390
297	estradiol	38.53	C_18_H_24_O_2_	[M + H]^+^	273.1845	255.1007, 248.4772, 153.0180, 119.0856
298	licoflavone A	38.57	C_20_H_18_O_4_	[M + H]^+^	323.1272	280.0719, 267.0648, 254.0570, 239.0700
299	irisolidone	38.67	C_17_H_14_O_6_	[M + H]^+^	315.0856	297.0751, 226.0619, 199.0751, 153.0182
				[M − H]^−^	313.0715	295.0610, 270.0479, 224.0468, 167.2795
300	1-methoxyphaseollidin	38.68	C_21_H_22_O_5_	[M + H]^+^	355.1532	299.0548, 221.1169, 165.0546, 123.0441
				[M − H]^−^	353.1393	338.1162, 292.0359, 253.0505, 150.0311
301	cupressuflavone	38.69	C_30_H_18_O_10_	[M + H]^+^	539.0958	497.0887, 403.0439, 377.0645, 335.0543
				[M − H]^−^	537.0822	521.2611, 505.2242, 375.0520, 266.9636
302	licoarylcoumarin	38.69	C_21_H_20_O_6_	[M + H]^+^	369.1324	313.0699, 271.0596, 243.0647, 147.0439
303	isoformononetin	38.71	C_16_H_12_O_4_	[M + H]^+^	269.0803	251.0697, 241.0828, 237.0537, 107.0855
				[M − H]^−^	267.0660	252.0424, 241.0503, 197.0604, 96.9588
304	kakkasaponin I	38.72	C_47_H_76_O_16_	[M − H]^−^	895.5067	877.5569, 763.6689, 678.9240, 509.4025
305	*β*-amyrone	38.76	C_30_H_48_O	[M + H]^+^	425.3767	/
306	tuberosin	38.82	C_20_H_18_O_5_	[M − H]^−^	337.1080	309.0397, 281.0454, 254.0585, 203.1068
307	glicophenone	38.83	C_20_H_22_O_6_	[M + H]^+^	359.1482	301.0710, 283.0596, 175.0389, 153.0545
				[M − H]^−^	357.1341	247.0974, 232.0737, 189.0186, 109.0282
308	7,4′-dihydroxy-3′-methoxyisoflavan	38.83	C_16_H_16_O_4_	[M + H]^+^	273.1118	245.1898, 163.0750, 137.0596, 123.0442
				[M − H]^−^	271.0973	241.0499, 225.0550, 197.0596, 181.0652
309	2′,3′-dihydro-7,7′-dihydroxy-5′-methoxy-2′,2′-dimethyl[3,6′-bi-4H-1-benzopyran]-4-one	38.89	C_21_H_20_O_6_	[M + H]^+^	369.1324	313.0699, 285.0752, 270.0518, 243.0648
				[M − H]^−^	367.1182	337.0717, 309.0403, 256.0376, 203.0708
310	3,4-didehydroglabridin	38.98	C_20_H_18_O_4_	[M + H]^+^	323.1272	267.0648, 255.0647, 239.0698, 95.0163
				[M − H]^−^	321.1129	277.0503, 265.0505, 252.0424, 149.0598
311	glyasperin C	38.98	C_21_H_24_O_5_	[M + H]^+^	357.1689	301.1063, 221.1165, 165.0546, 123.0441
				[M − H]^−^	355.1547	298.0483, 229.0865, 174.0313, 125.0232
312	neouralenol	39.01	C_20_H_18_O_7_	[M + H]^+^	371.1119	315.0856, 268.2631, 183.0287, 165.0181
				[M − H]^−^	369.0976	351.0870, 310.0444, 283.0975, 193.0135
313	phaseol	39.05	C_20_H_16_O_5_	[M + H]^+^	337.1065	319.0956, 283.0596, 255.0646, 163.0388
314	eurycarpin A	39.08	C_20_H_18_O_5_	[M + H]^+^	339.1219	322.2490, 293.0592, 163.0388, 114.0915
				[M − H]^−^	337.1080	293.1182, 268.0376, 224.0470, 135.0077
315	glyurallin A	39.10	C_21_H_20_O_5_	[M − H]^−^	351.1236	335.0564, 323.0929, 308.0317, 191.0711
316	sophoraisoflavone A	39.10	C_20_H_16_O_6_	[M + H]^+^	353.1014	335.0906, 325.1064, 283.02599, 191.0343
317	licocoumarone	39.11	C_20_H_20_O_5_	[M + H]^+^	341.1379	323.1265, 267.0648, 209.1646, 114.0915
				[M − H]^−^	339.1233	296.0677, 268.0377, 219.0656, 119.0490
318	dehydrovomifoliol	39.18	C_13_H_18_O_3_	[M + H]^+^	223.1326	135.1167, 107.0858, 81.0704
				[M − H]^−^	221.1177	205.1224, 164.0829, 148.0516, 118.5610
319	fallacinol	39.20	C_16_H_12_O_6_	[M + H]^+^	301.0701	283.0598, 269.0440, 227.0701, 199.0752
				[M − H]^−^	299.0557	284.0317, 255.0649, 240.0422, 212.0468
320	genkwanin	39.23	C_16_H_12_O_5_	[M + H]^+^	285.0751	270.0519, 253.0494, 225.0542, 137.0233
				[M − H]^−^	283.0609	268.0378, 240.0423, 186.6367, 118.3947
321	kanzonol U	39.25	C_19_H_16_O_4_	[M + H]^+^	309.1115	/
322	2,3-dehydrokievitone	39.26	C_20_H_18_O_6_	[M + H]^+^	355.1169	337.1066, 229.0854, 179.0338, 123.0442
				[M − H]^−^	353.1027	284.0319, 243.1021, 216.0419, 201.0915
323	2,3,4-trimethyl-5-phenyloxazolidine	39.28	C_12_H_17_NO	[M + H]^+^	192.1382	133.1011, 119.0493, 91.0547
324	pratensein	39.29	C_16_H_12_O_6_	[M + H]^+^	301.0701	283.0598, 269.0440, 227.0701, 199.0752
325	lupenone	39.32	C_30_H_48_O	[M + H]^+^	425.3767	/
326	corylifol B	39.35	C_20_H_20_O_5_	[M + H]^+^	341.1379	267.0648, 209.1646, 114.0916
				[M − H]^−^	339.1233	269.0453, 233.0818, 187.1117, 167.0340
327	4-*O*-methylglabridin	39.36	C_21_H_22_O_4_	[M + H]^+^	339.1586	322.2483, 209.1644, 114.0916, 95.0163
328	luteone	39.43	C_20_H_18_O_6_	[M + H]^+^	355.1169	338.3415, 299.0540, 267.0284, 239.0334
				[M − H]^−^	353.1027	257.0063, 227.0702, 165.0179, 125.0232
329	glyinflanin H	39.43	C_19_H_16_O_4_	[M + H]^+^	309.1115	291.1940, 223.0596, 113.0600, 95.0163
330	butyl octyl phthalate	39.44	C_20_H_30_O_4_	[M − H]^−^	333.2062	293.0450, 281.0451, 252.0419, 201.0916
331	glycyrrhetic acid 3-*O*-glucuronide	39.45	C_36_H_54_O_10_	[M + H]^+^	647.3769	453.3359, 357.2422, 285.2203, 121.1012
				[M − H]^−^	645.3641	580.9614, 521.2628, 469.3308, 322.6431
332	glyasperin A	39.46	C_25_H_26_O_6_	[M − H]^−^	421.1654	403.9289, 353.1024, 312.0273, 280.0371
333	1-methoxyphaseollin	39.47	C_21_H_20_O_5_	[M − H]^−^	351.1236	294.4448, 243.1023, 227.0710, 125.0232
334	licochalcone D	39.53	C_21_H_22_O_5_	[M + H]^+^	355.1532	338.3410, 311.0542, 193.0494, 135.0440
335	wighteone	39.54	C_20_H_18_O_5_	[M + H]^+^	339.1219	321.2453, 311.0548, 209.1647, 114.0916
				[M − H]^−^	337.1080	321.0765, 309.1127, 253.0500, 209.0596
336	2′-*O*-demethylbidwillol B	39.54	C_19_H_18_O_4_	[M + H]^+^	311.1269	293.1166, 278.0932, 263.0694, 95.0163
337	glycyrol	39.63	C_21_H_18_O_6_	[M + H]^+^	367.1168	337.0697, 227.0702, 167.0337, 91.0547
				[M − H]^−^	365.1026	335.0560, 307.0247, 295.0245, 254.0220
338	3-hydroxyglabrol	39.65	C_25_H_28_O_5_	[M − H]^−^	407.1863	387.2756, 371.2437, 150.9878, 93.0001
339	kumatakenin	39.72	C_17_H_14_O_6_	[M + H]^+^	315.0856	255.0647, 227.0699, 153.0180, 60.0452
				[M − H]^−^	313.0715	295.0606, 283.0609, 267.0663, 239.0712
340	eriodictyol	39.73	C_15_H_12_O_6_	[M + H]^+^	289.0697	271.0959, 229.0855, 163.0388, 153.0181
				[M − H]^−^	287.0559	272.0326, 258.0119, 216.0419, 155.1432
341	licoflavone B	39.74	C_25_H_26_O_4_	[M + H]^+^	391.1897	358.2020, 323.1262, 267.0647, 195.0430
342	gancaonin U	39.76	C_24_H_28_O_4_	[M − H]^−^	379.1908	/
343	dehydroglyceollin I	39.78	C_20_H_16_O_4_	[M + H]^+^	321.1115	306.0873, 187.0752, 159.0803, 147.0439
				[M − H]^−^	319.0970	303.0658, 289.0504, 243.0657, 161.0233
344	tectorigenin	39.80	C_16_H_12_O_6_	[M + H]^+^	301.0701	283.0598, 255.0646, 227.0698, 199.0748
				[M − H]^−^	299.0557	284.0317, 267.0301, 240.0422, 212.0468
345	derrone	39.87	C_20_H_16_O_5_	[M + H]^+^	337.1065	309.1118, 267.0650, 225.0545, 91.0549
				[M − H]^−^	335.0921	319.0606, 305.0436, 278.3866, 158.8393
346	abyssinone II	39.91	C_20_H_20_O_4_	[M + H]^+^	325.1429	269.0804, 241.0850, 135.0440, 95.0163
				[M − H]^−^	323.1283	308.1031, 201.0914, 187.0761, 135.0441
347	corylin	39.96	C_20_H_16_O_4_	[M + H]^+^	321.1115	306.0870, 279.0649, 265.0488, 137.0232
348	ephedradine A	39.98	C_28_H_36_N_4_O_4_	[M + H]^+^	493.2800	465.2870, 394.2122, 219.1489, 120.0809
349	kudzusapogenol A	40.02	C_30_H_50_O_5_	[M − H]^−^	489.3575	/
350	kanzonol Y	40.04	C_25_H_30_O_5_	[M − H]^−^	409.2015	391.2520, 373.2436, 235.0971, 177.0912
351	kanzonol W	40.11	C_20_H_16_O_5_	[M + H]^+^	337.1065	321.1119, 281.0443, 253.0488, 163.0388
				[M − H]^−^	335.0921	320.0677, 291.1024, 199.0758, 135.0078
352	isoglycyrol	40.13	C_21_H_18_O_6_	[M + H]^+^	367.1168	349.1074, 325.0702, 291.0630, 167.0338
				[M − H]^−^	365.1026	349.0708, 309.0393, 216.0423, 192.0055
353	licoisoflavone B	40.17	C_20_H_16_O_6_	[M + H]^+^	353.1014	311.0558, 299.0544, 153.0180, 95.0163
				[M − H]^−^	351.0869	337.0660, 283.0974, 241.0864, 199.0756
354	6,8-diprenylgenistein	40.26	C_25_H_26_O_5_	[M + H]^+^	407.1844	339.1198, 283.0596, 237.0534, 91.0547
				[M − H]^−^	405.1704	387.2755, 371.2439, 281.0460, 150.9878
355	parvisoflavone A	40.29	C_20_H_16_O_6_	[M + H]^+^	353.1014	335.0906, 325.1068, 191.0328, 153.0180
356	licoricidin	40.30	C_26_H_32_O_5_	[M + H]^+^	425.2315	369.1328, 313.0703, 175.0388, 139.0389
357	kanzonol C	40.31	C_25_H_28_O_4_	[M + H]^+^	393.2051	376.1539, 329.0271, 268.0656, 215.0684
* 358	emodin	40.43	C_15_H_10_O_5_	[M + H]^+^	271.0594	243.0650, 229.0493, 197.0596, 173.0591
				[M − H]^−^	269.0450	241.0503, 225.0551, 197.0597, 181.0647
359	apigenin	40.43	C_15_H_10_O_5_	[M + H]^+^	271.0594	229.0495, 201.0543, 173.0597, 91.0548
				[M − H]^−^	269.0450	241.0501, 225.0551, 210.0314, 181.0644
360	genistein	40.44	C_15_H_10_O_5_	[M + H]^+^	271.0594	243.0642, 229.0495, 201.0543, 371.0596
				[M − H]^−^	269.0450	241.0504, 225.0550, 197.0597, 181.0647
361	lupalbigenin	40.45	C_25_H_26_O_5_	[M + H]^+^	407.1844	373.1034, 283.0597, 213.0541, 149.0232
362	angustone A	40.51	C_25_H_26_O_6_	[M − H]^−^	421.1654	404.9251, 352.0951, 269.0453, 201.0913
363	dehydroglyasperin D	40.59	C_22_H_24_O_5_	[M + H]^+^	369.1688	313.0700, 295.0597, 197.0441, 179.0337
				[M − H]^−^	367.1545	351.9669, 322.9651, 269.0455, 240.0420
364	corymbosin	40.63	C_19_H_18_O_7_	[M + H]^+^	359.1118	329.0648, 313.0695, 269.0804, 95.0163
365	euchrenone a5	40.64	C_25_H_26_O_4_	[M + H]^+^	391.1897	358.2020, 267.0647, 239.0701, 149.0236
				[M − H]^−^	389.1751	319.0978, 298.0473, 266.0580, 195.1691
366	paratocarpin L	40.75	C_25_H_28_O_5_	[M − H]^−^	407.1863	/
367	diisobutyl phthalate	40.79	C_16_H_22_O_4_	[M + H]^+^	279.1583	167.0340, 149.0232, 121.0284, 57.0706
				[M − H]^−^	277.1438	245.3889, 193.7951, 134.0361, 121.0283
368	glyurallin B	40.79	C_25_H_26_O_6_	[M + H]^+^	423.1793	/
369	angustone B	40.82	C_25_H_24_O_6_	[M + H]^+^	421.1637	365.1014, 309.0388, 281.0439, 140.0342
				[M − H]^−^	419.1497	402.9280, 375.0866, 363.0872, 308.0323
370	licoagrocarpin	40.93	C_21_H_22_O_4_	[M + H]^+^	339.1586	/
371	palmitic acid	41.04	C_16_H_32_O_2_	[M − H]^−^	255.2322	170.3186, 162.0524, 116.9273, 74.0233
372	2′-hydroxyisolupalbigenin	41.11	C_25_H_26_O_6_	[M − H]^−^	421.1654	404.9261, 363.0872, 227.0711, 193.0862
373	butesuperin A	41.15	C_26_H_22_O_8_	[M + H]^+^	463.1375	445.1275, 283.0597, 255.0647, 161.0594
* 374	luteolin	41.16	C_15_H_10_O_6_	[M − H]^−^	285.0402	268.9432, 257.0451, 242.0536, 196.0504
375	sophoracoumestan A	41.19	C_20_H_14_O_5_	[M + H]^+^	335.0909	320.0672, 307.0952, 292.0722, 137.0237
* 376	glycyrrhetic acid	41.41	C_30_H_46_O_4_	[M + H]^+^	471.3456	317.2107, 269.0803, 189.1636, 121.1013
377	8-prenylphaseollinisoflavan	41.74	C_25_H_28_O_4_	[M + H]^+^	393.2051	339.0701, 269.0807, 167.0337, 149.0232
				[M − H]^−^	391.1912	289.1443, 271.1335, 187.0393, 119.0490
378	stearic acid	41.75	C_18_H_36_O_2_	[M − H]^−^	283.2635	/
379	puerarol	41.75	C_25_H_24_O_5_	[M + H]^+^	405.1683	319.0950, 281.0439, 209.0591, 171.0138
				[M − H]^−^	403.1546	387.2754, 371.2439, 333.0764, 150.9877
380	hederagenin	41.79	C_30_H_48_O_4_	[M + H]^+^	473.3615	310.8371, 189.1640, 133.1015, 59.0162
				[M − H]^−^	471.3470	429.2146, 403.1549, 319.0597, 280.0376

*: Verified with its reference standard.

**Table 2:** Quantitative method validation results of YPG.

Main Components	Calibration Curve	Linear Range (mg/mL)	R^2^	LOD (μg/mL)	LOQ (μg/mL)	Repeatability RSD (%)	Stability RSD (%)	Intermediate Precision (%, *n* = 12)
chlorogenic acid	y = 12,757x – 376.29	0.05–2.00	0.9991	2.13	6.47	1.18%	1.37%	2.82%
puerarin	y = 37,995x + 303.18	0.05–1.60	0.9990	1.85	5.61	1.23%	2.60%	2.00%
3′-methoxypuerarin	y = 25,755x + 64.034	0.04–1.50	0.9998	1.26	3.83	2.94%	1.09%	2.81%
polydatin	y = 9872.5x – 89.081	0.03–1.00	0.9992	1.25	3.80	2.27%	3.10%	3.45%
glycyrrhizic acid	y = 7190.5x + 101.33	0.02–1.00	0.9994	0.77	2.33	2.03%	1.30%	3.89%
emodin	y = 33,371x + 138.83	0.01–0.50	0.9992	0.38	1.14	1.26%	3.59%	3.53%

**Table 3:** Quantitative analysis, total phenol content, and DPPH radical scavenging results of YPG.

Main Components	Sample S1 (mg/g)	Sample S2 (mg/g)	Sample S3 (mg/g)	Content (mg/g)	Scavenging Percentage of DPPH Radical (%)
chlorogenic acid	33.16	33.72	35.56	34.15 ± 1.25	59.2
puerarin	27.30	28.15	29.46	28.30 ± 1.09	49.6
3′-methoxypuerarin	9.73	9.66	9.50	9.63 ± 0.12	58.9
polydatin	10.29	10.76	11.43	10.83 ± 0.57	58.0
glycyrrhizic acid	3.18	2.85	3.94	3.33 ± 0.56	54.6
emodin	4.15	4.48	3.80	4.14 ± 0.34	30.3
total phenol	144.11	147.89	147.99	144.66 ± 2.21	/

## Data Availability

The data presented in this study are available in article and Supplementary Materials. Additional data that support the findings of this study are also available on request from the corresponding author.

## Supplementary Materials

The following supporting information can be downloaded at https://www.mdpi.com/article/10.3390/molecules29102300/s1: Table S1: Mass spectrometric information of chemical components of Yinhua Pinggan Granule; Table S2: Recovery of six representative components. Table S3: The contents of representative components of YPG under different extraction conditions. Figure S1. The HPLC chromatograms at 254 nm of YPG before and after the reaction with DPPH. Refs. [25,26,27,28,29,30,31,32,33,34,35,36,37,38,39,40,41,42,43,44,45,46,47,48,49,50,51,52,53,54,55,56,57,58,59,60] are cited in the Supplementary Materials.
