# Supplementary material for: Qualitative and Quantitative Analysis of Chemical Components in Yinhua Pinggan Granule with High-Performance Liquid Chromatography Coupled with Q-Exactive Mass Spectrometry
Source: Molecules. 2024 May 14;29(10):2300. doi: 10.3390/molecules29102300 (PMC11124461; doi:10.3390/molecules29102300)
Supplement: Supplementary file 1 [file molecules-29-02300-s001.zip › molecules-2995188-supplementary.pdf]

Qualitative and quantitative analysis of chemical components in Yinhua Pinggan Granule with high performance liquid chromatography coupled with Q-Exactive mass spectrometry

Imranjan Yalkun †, Haofang Wan †, Lulu Ye, Li Yu, Yu He, Chang Li * and Haitong Wan *

Zhejiang Chinese Medical University, Hangzhou 310053, China
* Correspondence: lichang@zju.edu.cn (C.L.); whtong@126.com (H.W.); Tel./Fax: +86-571-86613716 (C.L.&H.W.)
† The authors contributed equally to this work.

Supporting information

Table of contents

Table S1. Mass spectrometric information of chemical components of Yinhua Pinggan Granule 2

Table S2. Recovery of Six Representative Components 32

Table S3. The content of representative components of YPG under different extraction conditions 33

Figure S1. The HPLC chromatogram at 254 nm of YPG before and after reaction with DPPH 34

Table S1. Mass spectrometric information of chemical components of Yinhua Pinggan Granule

No.	Name	Structure	Type	Source	RT (min)	Ion Type	Product ion(*m/z*)	Reference
1	ephedrannin A	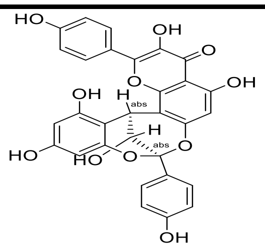	Fla	HE	3.40	[M+H]$^+$	395.1281,215.0650,<br>177.0543,145.0284	[25]
2	glucose	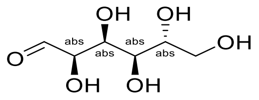	Oth	LJT	3.63	[M+H]$^+$<br>[M-H]$^-$	163.0792,144.0655,<br>109.0286,81.0340<br>161.0445,141.0181,<br>117.0181,87.0073	[26]
3	secologanic acid	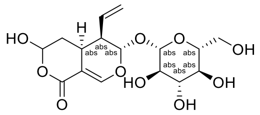	Ter	LJT	3.67	[M-H]$^-$	347.9473,189.0156,<br>161.0234,135.0440	[26]
4	*D*-mannitol	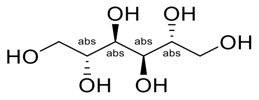	Oth	PCR	3.82	[M+H]$^+$	147.0650,129.0543,<br>104.1073,69.0342	[27]
5	sucrose	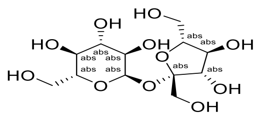	Oth	PLR	3.86	[M+H]$^+$<br>[M-H]$^-$	306.1183,145.0495,<br>127.0390,85.0290<br>261.7968,179.0559,<br>113.0230,59.0125	[28]
6	allantoin	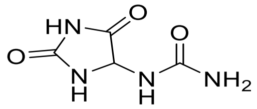	Alk	PLR	3.99	[M+H]$^+$	142.0862,114.0915,<br>99.0193,70.0658	[28]
7	quinic acid	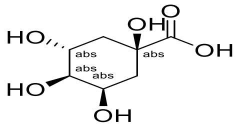	Oa	LJT	4.13	[M+H]$^+$	157.0492,147.0652,<br>129.0546,111.0443	[29]
8	salicylic acid	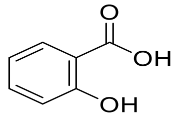	Pa	PLR	4.14	[M+H]$^+$	122.0714,111.0443,<br>97.0287,85.0289	[28]
9	4-aminophenol	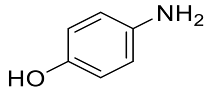	Op	HE	5.72	[M+H]$^+$	87.0046,81.0340,<br>78.9949	[25]
10	citric acid	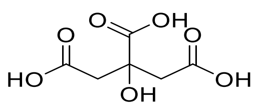	Oa	HE	5.59	[M+H]$^+$<br>[M-H]$^-$	161.0595,151.0388,<br>133.0647,105.0702<br>173.0085,129.0180,<br>111.0075,87.0071	[30]
11	guanosine	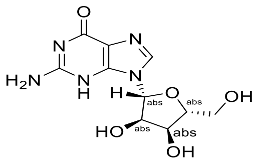	Oth	LJT	6.34	[M+H]$^+$<br>[M-H]$^-$	258.4957,152.0566,<br>135.0302,110.0351<br>169.3754,150.0411,<br>88.1632,61.9871	[26]
12	adenosine	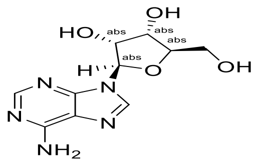	Oth	LJT	6.46	[M+H]$^+$	213.3238,169.7115,<br>136.0617,85.0288	[26]

13	gallic acid	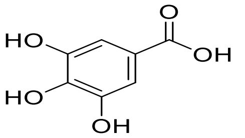	Pa	PLR	7.23	$[M+H]^+$	154.0974,130.0863, 115.0392,70.0658	[28]
14	coumalic acid	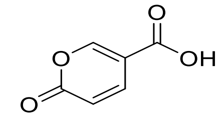	Oa	HE	7.40	$[M+H]^+$	113.9639,90.9481, 72.9378,56.9430	[31]
15	tachioside	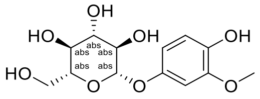	Op	PCR	8.05	$[M-H]^-$	283.1918,257.0457, 221.1909,151.0029	[27]
16	isotachioside	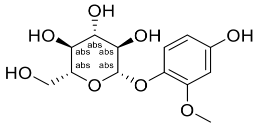	Op	PCR	8.27	$[M-H]^-$	284.0321,243.0658, 178.9978,151.0026	[27]
17	ephedroxane	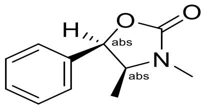	Alk	HE	8.27	$[M+H]^+$	164.9844,146.9612, 106.0654,87.0445	[31]
18	quinaldic acid	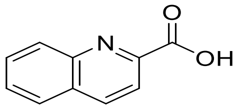	Alk	HE	8.27	$[M+H]^+$	146.9612,128.9507, 105.9351,55.9352	[25]
19	hordenine	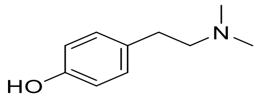	Alk	HE	8.56	$[M+H]^+$	151.0101,121.0649, 103.0546,93.0703	[32]
20	leonuriside A	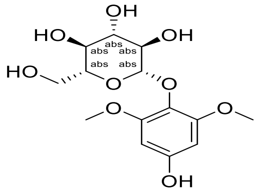	Op	PCR	8.99	$[M-H]^-$	285.0384,253.0501, 169.0130,125.0231	[27]
21	4-vinylguaiacol	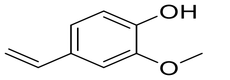	Op	ASA	9.19	$[M+H]^+$	133.0761,123.9456, 119.0493,91.0547	[33]
22	*7α*-morroniside	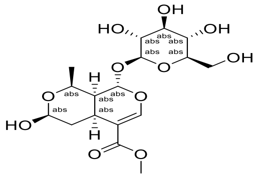	Ter	LJT	9.50	$[M-H]^-$	371.0939,243.0672, 191.0199,111.0070	[26]
23	tetramethylpyrazine	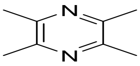	Alk	HE	9.83	$[M+H]^+$	111.0080,93.0704, 68.9978	[25]
24	shuangkangsu	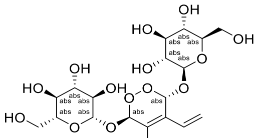	Oth	LJT	10.49	$[M-H]^-$	447.2225,431.0965, 269.0450,169.0130	[26]
25	*epi*-gallocatechin	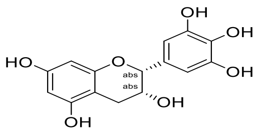	Fla	HE	10.69	$[M+H]^+$<br>$[M-H]^-$	289.1790,243.1704, 208.9966,139.0389<br>247.5994,219.0664, 165.0182,125.0232	[34]
26	robinin	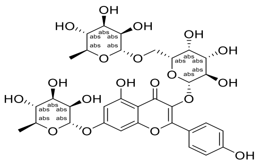	Fg	PLR	10.73	$[M+H]^+$	678.4389,579.1688, 381.0964,297.0752	[28]

27	5-(hydroxymethyl)furfural	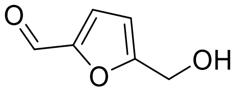	Oth	LJT	10.85	[M+H]+	111.9689,110.0238,<br>84.9603,55.9352	[26]
28	*N*-methylbenzylamine	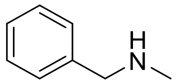	Alk	HE	10.90	[M+H]+	107.0732,105.0336,<br>95.0495,88.0237	[34]
29	vicenin-2	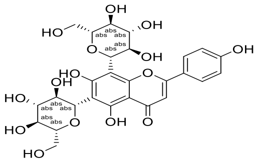	Fg	GR	11.00	[M+H]+<br>[M-H]-	433.1127,415.1019,<br>313.0700,283.0596<br>539.2719,521.2607,<br>463.2737,226.9863	[35]
30	4'-hydroxyacetophenone	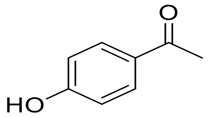	Op	PCR	11.09	[M+H]+	122.0364,116.9720,<br>95.0497,55.9352	[27]
31	6-hydroxykynurenic acid	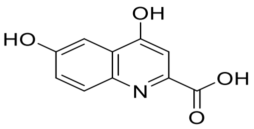	Alk	HE	11.35	[M+H]+<br>[M-H]-	178.0497,148.1121,<br>117.0699,90.0797<br>168.1795,160.0393,<br>132.0446,110.1680	[25]
32	chlorogenic acid butyl ester	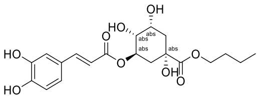	Pa	LJT	11.36	[M-H]-	365.0681,337.0357,<br>241.0023,169.0135	[36]
33	mirificin-4'-*O*-glucoside	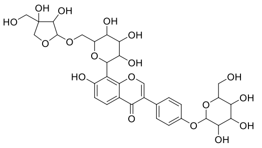	Fg	PLR	11.73	[M+H]+<br>[M-H]-	579.1680,417.1183,<br>399.1065,297.0753<br>487.1239,457.1142,<br>294.0533,266.0583	[28]
34	mandelonitrile	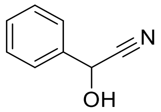	Oth	ASA	11.82	[M+H]+	106.0654,91.0544,<br>79.0548	[33]
35	kakkalide	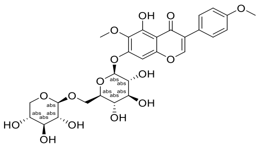	Fg	PLR	11.83	[M+H]+<br>[M-H]-	447.1279,411.1072,<br>327.0857,297.0755<br>588.1254,487.1243,<br>309.0403,281.0458	[28]
36	mahuannin A	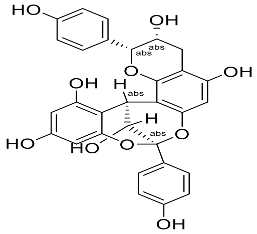	Fla	HE	11.87	[M-H]-	528.5940,497.2623,<br>381.1213,265.0987	[25]
37	2,6-dihydroxybenzoic acid	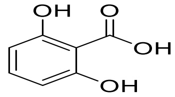	Pa	PCR	11.92	[M+H]+	308.2842,237.3755,<br>151.0375,107.0485	[37]
38	8-*epi*-loganic acid	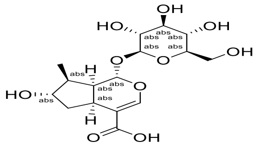	Ter	LJT	11.96	[M+H]+<br>[M-H]-	357.1662,339.1559,<br>265.0587,237.0277<br>315.8566,265.2063,<br>201.0163,113.0231	[38]
39	swertiamarin	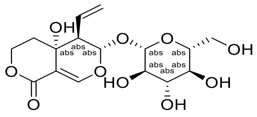	Ter	LJT	12.02	[M-H]-	357.0130,295.0618,<br>201.0158,135.0433	[26]
40	8-*epi*-Loganin	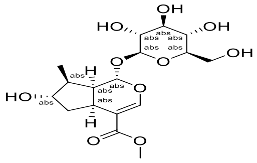	Ter	LJT	12.30	[M-H]-	371.8339,345.1187,<br>227.0693,185.0593	[38]

41	5-methoxysalicylic acid	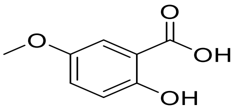	Pa	HE	12.31	$[M+H]^+$	151.0391,128.9508,<br>111.0444,93.0339	[25]
42	norephedrine	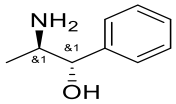	Alk	HE	12.65	$[M+H]^+$	134.0964,117.0700,<br>115.0545,91.0547	[34]
43	7-*epi*-vogeloside	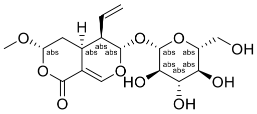	Ter	LJT	12.81	$[M-H]^-$	341.1095,272.9591,<br>227.0690,179.0566	[26]
44	loganic acid	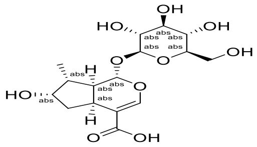	Ter	LJT	12.88	$[M-H]^-$	287.1191,201.0159,<br>189.0158,135.0440	[38]
45	protocatechuic acid	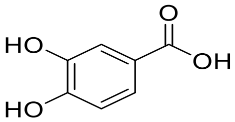	Pa	LJT	12.90	$[M+H]^+$	137.0233,117.0701,<br>107.0495,72.9379	[36]
46	polygalin B	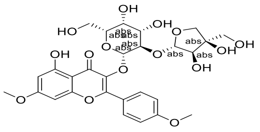	Fg	PCR	13.12	$[M+H]^+$<br>$[M-H]^-$	555.7846,447.1268,<br>285.0752,270.0516<br>460.8990,325.0714,<br>310.0492,282.0534	[27]
47	lonijaposide B	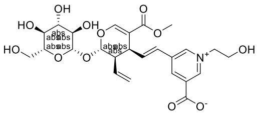	Alk	LJT	13.15	$[M-H]^-$	511.3804,375.0705,<br>335.0791,201.0155	[26]
48	3'-hydroxypuerarin	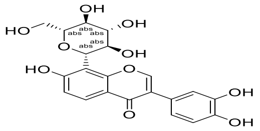	Fg	PLR	13.17	$[M-H]^-$	415.0348,311.0557,<br>283.0609,255.0659	[28]
49	norpseudoephedrine	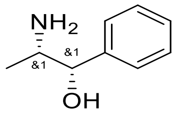	Alk	HE	13.19	$[M+H]^+$	134.0964,117.0700,<br>106.0655	[34]
50	loganin	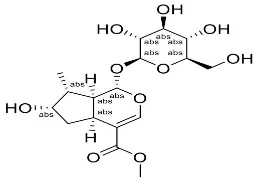	Ter	LJT	13.30	$[M-H]^-$	371.8339,326.0798,<br>227.0693,185.0593	[26]
51	secologanoside	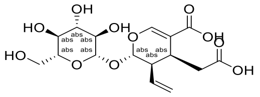	Ter	LJT	13.43	$[M+H]^+$<br>$[M-H]^-$	239.0796,241.0385,<br>163.0388,151.0389<br>280.5215,194.8876,<br>121.0647,95.0489	[26]
52	leucodelphidin	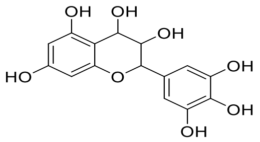	Fla	HE	13.74	$[M-H]^-$	305.2140,265.0519,<br>253.0507,186.9385	[31]
53	secologanin	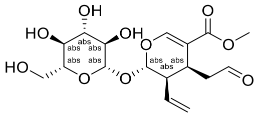	Ter	LJT	13.83	$[M-H]^-$	/	[26]
54	glucoisoliquiritin	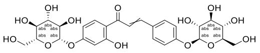	Fg	GR	14.04	$[M-H]^-$	529.4413,491.9212,<br>463.1199,255.0662	[35]
55	glucoliquiritin apioside	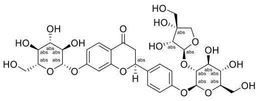	Fg	GR	14.07	$[M-H]^-$	678.4865,549.119,<br>457.1141,255.0664	[35]

56	chlorogenic acid	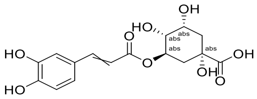	Pa	LJT	14.15	[M+H]+<br>[M-H]-	163.0388,135.0440<br>191.0553,161.0234,<br>135.0440,127.0385	[38]
57	neochlorogenic acid	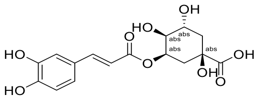	Pa	LJT	14.22	[M+H]+	338.1603,289.0706,<br>235.0594,163.0390	[36]
58	catechin	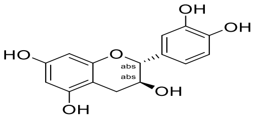	Fla	HE	14.25	[M+H]+<br>[M-H]-	255.7885,107.0648,<br>139.0389,123.0442<br>245.0817,203.0705,<br>151.0387.123.0439	[34]
59	ephedrine	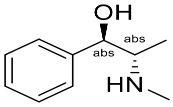	Alk	HE	14.36	[M+H]+	148.1119,133.0886,<br>117.0700,91.0548	[34]
60	cryptochlorogenic acid	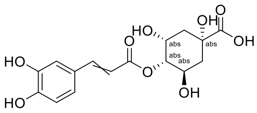	Pa	LJT	14.37	[M+H]+	337.0915,235.0589,<br>205.0494,163.0388	[38]
61	pseudoephedrine	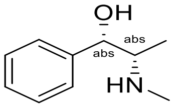	Alk	HE	14.43	[M+H]+	148.1119,133.0887,<br>117.0701,91.0546	[34]
62	ephedrannin D4	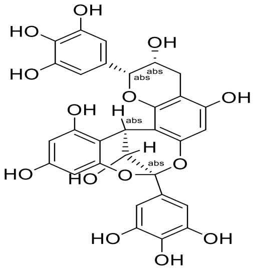	Fla	HE	14.44	[M-H]-	563.1151,487.1202,<br>413.0903,267.0680	[32]
63	puerarin 6''-*O*-xyloside	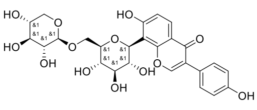	Fg	PLR	14.61	[M+H]+<br>[M-H]-	417.1175,381.0964,<br>297.0753,267.0648<br>437.0846,295.0609,<br>277.0504,267.0661	[28]
64	chrysoeriol 7-*O*-neohesperidoside	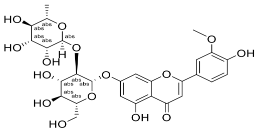	Fg	LJT	14.65	[M+H]+<br>[M-H]-	447.1284,429.1182,<br>327.0859,285.0755<br>547.1422,487.1246,<br>295.0607,267.0660	[26]
65	p-hydroxybenzoic acid	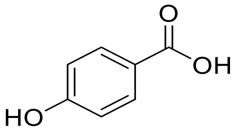	Pa	LJT	14.70	[M+H]+	121.0286,111.0443,<br>93.0339	[34]
66	amygdalin	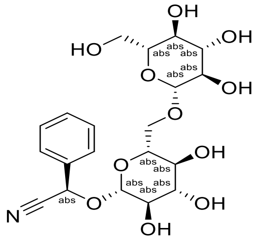	Oth	ASA	14.72	[M+H]+<br>[M-H]-	416.7005,325.1123,<br>296.1126,158.0599<br>382.6093,323.0968,<br>256.1356,161.0449	[33]
67	methyl caffeate	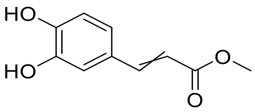	Pa	LJT	14.83	[M+H]+	177.0544,163.0388,<br>145.0283,117.0336	[36]
68	3,4-dimethyl-5-phenyloxazolidine	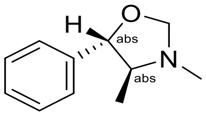	Alk	HE	14.94	[M+H]+	162.1274,147.1040,<br>117.0700,105.0702	[34]
69	apigenin 5-rhamnoside		Fg	HE	15.01	[M+H]+	381.0964,321.0743,	[25]

		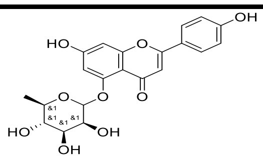					297.0753,267.0647	
						[M-H]$^-$	295.0608,267.0661,<br>253.0509,223.0762	
70	puerarin	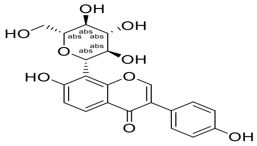	Fg	PLR	15.02	[M+H]$^+$<br>[M-H]$^-$	399.1072,381.0956,<br>363.0844,255.0646<br>295.0608,277.0507<br>267.0661	[28]
71	mirificin	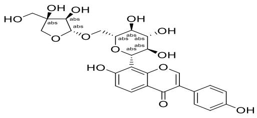	Fg	PLR	15.04	[M+H]$^+$<br>[M-H]$^-$	417.1168,399.1069,<br>297.0754,267.0648<br>418.4808,295.0609,<br>267.0660,114.2369	[28]
72	tectorigenin 7-*O*-xylosylglucoside	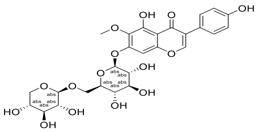	Fg	PLR	15.07	[M+H]$^+$<br>[M-H]$^-$	379.0811,325.0695,<br>216.0653,121.0283<br>495.0386,473.1082,<br>310.0499,282.0529	[28]
73	methylephedrine	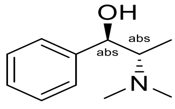	Alk	HE	15.14	[M+H]$^+$	162.1275,148.1076,<br>135.0804	[34]
74	methylpseudoephedrine	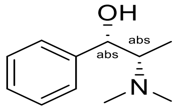	Alk	HE	15.18	[M+H]$^+$	162.1275,147.1042,<br>135.0803,117.0700	[34]
75	7-*O*-ethylsweroside	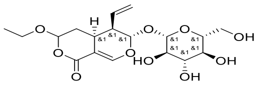	Ter	LJT	15.26	[M-H]$^-$	325.7480,269.1024,<br>253.0505,178.0263	[38]
76	isoviolanthin	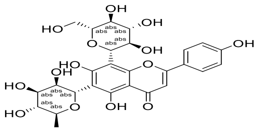	Fg	GR	15.47	[M+H]$^+$<br>[M-H]$^-$	417.1190,399.1072,<br>297.0753,267.0649<br>531.2839,518.0385,<br>283.0610,268.0376	[39]
77	3'-methoxypuerarin	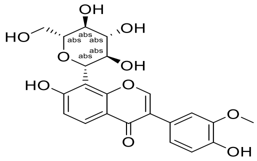	Fg	PLR	15.53	[M+H]$^+$<br>[M-H]$^-$	285.0751,270.0516,<br>225.0542,137.0232<br>430.0887,367.1027,<br>327.1080,215.0089	[28]
78	glycitin	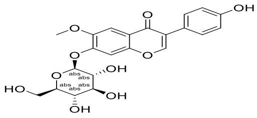	Fg	PLR	15.54	[M+H]$^+$<br>[M-H]$^-$	429.1190,411.1062,<br>327.0855,297.0754<br>379.8243,325.0714,<br>282.0530,254.0597	[28]
79	benzyl alcohol	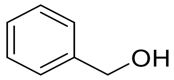	Oth	ASA	15.67	[M+H]$^+$	94.0148,91.0546,<br>87.0045,81.0704	[33]
80	methyl 4-hydroxycinnamate	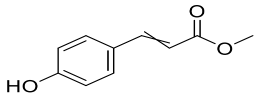	Pa	LJT	15.68	[M+H]$^+$	162.1276,147.1041,<br>117.0700,109.0651	[36]
81	*γ*-octalactone	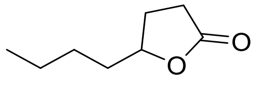	Oth	ASA	15.94	[M+H]$^+$	128.9508,116.9721,<br>113.9639,84.9603	[33]
82	*(6S_9R)*-roseoside	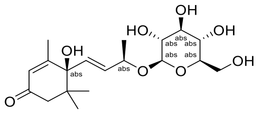	Ter	PLR	15.98	[M+H]$^+$	369.1341,297.0762,<br>267.0641,151.0388	[28]
83	syringin	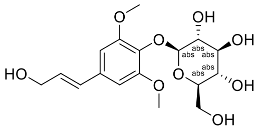	Op	LJT	15.98	[M+H]$^+$	308.0842,292.1605,<br>237.3755,151.0375	[26]

84	5,7-dihydroxyisobenzofuran	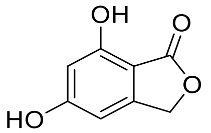	Op	PCR	15.99	[M+H]+	148.1120,133.0885,<br>117.0700,111.0442	[27]
85	kingiside	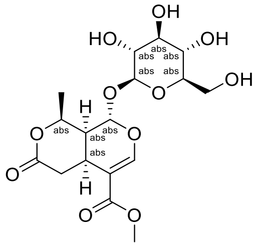	Ter	LJT	16.01	[M-H]-	371.1025,310.7592,<br>243.0664,174.8555	[26]
86	puerarin-7-*O*-glucoside	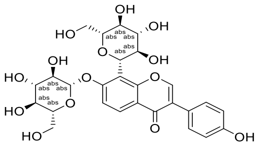	Fg	PLR	16.04	[M+H]+	561.1633,399.1074,<br>297.0753,267.0647	[28]
87	sweroside	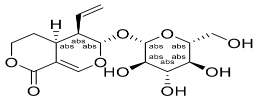	Ter	LJT	16.09	[M+H]+	297.8041,265.6036,<br>197.0808,127.0391	[26]
88	caffeic acid	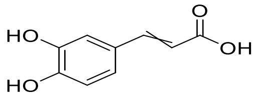	Pa	LJT	16.29	[M+H]+<br>[M-H]-	163.0388,145.0284,<br>135.0441,117.0337<br>164.0098,135.0440,<br>112.1822,107.0492	[36]
89	ephedrannin D1	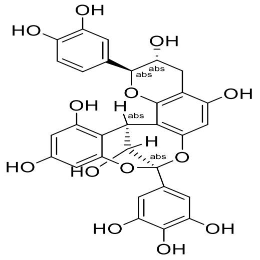	Fla	HE	16.53	[M+H]+	576.3641,447.1271,<br>327.0849,297.0745	[32]
90	5-*p*-coumaroylquinic acid	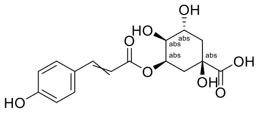	Pa	LJT	16.70	[M+H]+<br>[M-H]-	266.4322,245.8672,<br>147.0439,119.0494<br>191.0553,163.0390,<br>119.0470,93.0332	[38]
91	loniceracetalide A	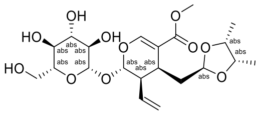	Ter	LJT	16.71	[M-H]-	/	[26]
92	piceatannol 3'-*O*-glucoside	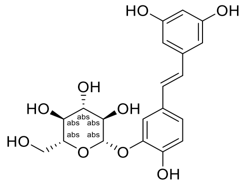	Op	PCR	16.77	[M-H]-	359.0753,243.0659,<br>201.0549,159.0440	[40]
93	lonijaposide D	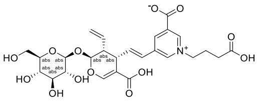	Alk	LJT	16.82	[M-H]-	550.4224,519.2438,<br>445.1137,325.0718	[26]
94	ephedralone	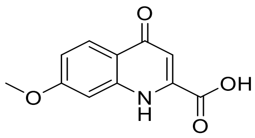	Alk	HE	16.90	[M+H]+<br>[M-H]-	192.0652,164.0699,<br>151.4024,119.0490<br>174.0550,159.0315,<br>144.0077,131.0365	[25]
95	neochlorogenic acid methyl ester	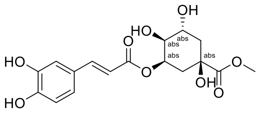	Pa	LJT	17.85	[M+H]+<br>[M-H]-	191.9904,177.0544,<br>145.0283,117.0336<br>255.0194,191.0552,<br>173.0447,134.0361	[41]
96	isoschaftoside		Fg	GR	17.86	[M+H]+	479.5669,415.1016,	[35]

		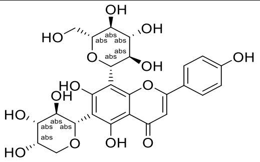				 [M-H]$^-$	313.0699,283.0596<br>341.0680,311.0559, 283.0609,149.0235	
97	2,6-dihydroxyphenylacetic acid	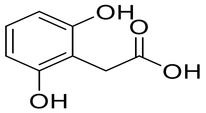	Pa	PCR	18.07	[M+H]$^+$	151.0389,146.9612, 128.9508,123.0441	[37]
98	schaftoside	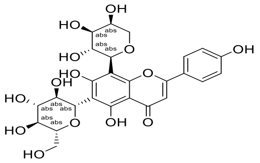	Fg	GR	18.35	[M+H]$^+$<br>[M-H]$^-$	520.6823,433.1122, 313.0700,283.0597<br>529.1846,341.0670, 311.0558,283.0610	[35]
99	vanillic acid	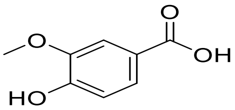	Pa	LJT	18.50	[M+H]$^+$	151.0388,146.9612, 128.9508,123.0442	[41]
100	flavoyadorinin B	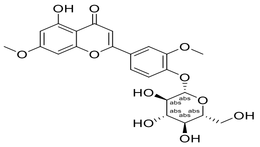	Fg	LJT	18.51	[M+H]$^+$	327.1659,279.0375, 204.5438,145.0499	[26]
101	ephedrannin D2	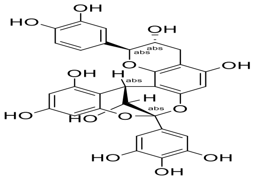	Fla	HE	18.54	[M-H]$^-$	547.1453,462.7491, 285.0408,253.0507	[32]
102	cinnamic acid	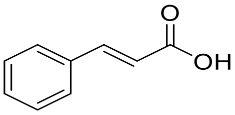	Oa	HE	18.59	[M+H]$^+$	133.0885,121.0648, 103.0548,95.0497	[34]
103	hydroxyphenylacetic acid	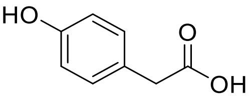	Pa	HE	18.65	[M+H]$^+$	135.1167,112.0395, 90.9481,72.9378	[34]
104	3-*O*-caffeoylshikimic acid	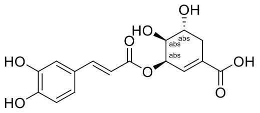	Pa	LJT	18.81	[M+H]$^+$<br>[M-H]$^-$	181.0494,163.0388, 145.0283,95.0495<br>269.1091,179.0345, 161.0233,133.0282	[29]
105	leucopelargonidin	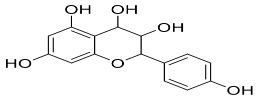	Fla	HE	18.82	[M+H]$^+$<br>[M-H]$^-$	273.0753,207.0647, 147.0440,139.0389<br>245.0818,203.0705, 151.0392,123.0441	[34]
106	licuraside	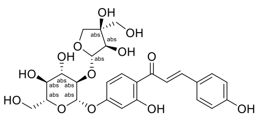	Fg	GR	19.43	[M-H]$^-$	502.1001,429.1062, 255.0660,119.0490	[39]
107	genistein 7-*O*-glucoside	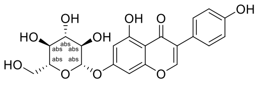	Fg	PLR	20.25	[M+H]$^+$	271.0595,215.0700, 137.0231	[28]
108	liquiritin apioside	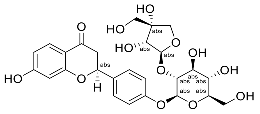	Fg	GR	20.29	[M-H]$^-$	482.5059,297.0778, 255.0660,135.0076	[43]
109	isoliquiritin apioside	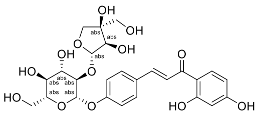	Fg	GR	20.34	[M-H]$^-$	488.6683,429.1148, 255.0659,135.0076	[43]

110	liquiritin	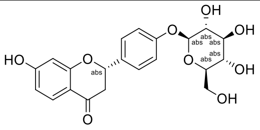	Fg	GR	20.38	$[M-H]^-$	402.1664,373.0210,<br>255.0662,119.0490	[35]
111	secologanin dimethyl acetal	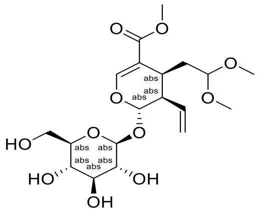	Ter	LJT	20.39	$[M-H]^-$	/	[26]
112	3-methoxyphenol	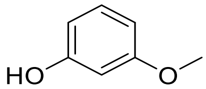	Op	ASA	20.50	$[M+H]^+$	102.9706,97.0287,<br>84.9602	[33]
113	piceid gallate A	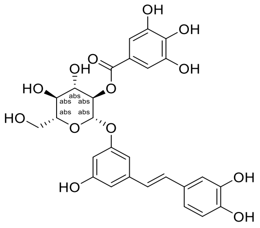	Fg	PCR	20.63	$[M-H]^-$	/	[40]
114	polydatin	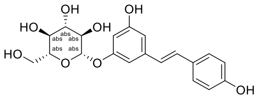	Op	PCR	20.92	$[M-H]^-$	227.0707,185.0598,<br>159.0808,143.0491	[27]
115	lonicerin	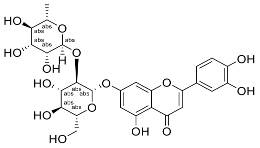	Fg	LJT	21.02	$[M+H]^+$<br>$[M-H]^-$	433.1108,313.0717,<br>271.0596,215.0697<br>430.4479,329.5679,<br>285.0396,227.0705	[26]
116	isoliquiritin	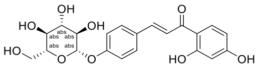	Fg	GR	21.24	$[M-H]^-$	255.0660,153.0182,<br>135.0075,119.0489	[35]
117	quercetin 3-glucoside	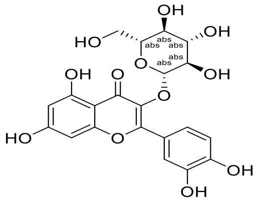	Fg	PLR	21.69	$[M-H]^-$	300.0272,271.0247,<br>255.0296,151.0027	[26]
118	kaempferol 7-*O*-glucopyranoside	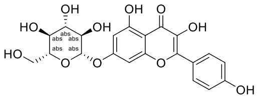	Fg	PLR	21.69	$[M+H]^+$<br>$[M-H]^-$	330.0535,287.0545,<br>203.4280,153.0181<br>410.9457,325.0732,<br>285.0397,256.0383	[28]
119	neoisoliquiritin	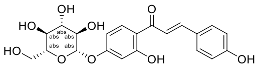	Fg	GR	22.95	$[M-H]^-$	374.0878,255.0660,<br>153.0183,135.0076	[35]
120	coumarin	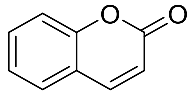	Oth	PCR	23.54	$[M+H]^+$	131.9743,119.0493,<br>113.9640	[27]
121	daidzein	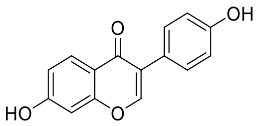	Fla	GR	23.56	$[M-H]^-$	224.0468,209.0598,<br>197.0602,135.0076	[39]
122	rhoifolin	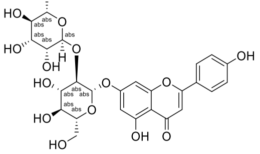	Fg	LJT	23.76	$[M+H]^+$	515.2410,429.1205,<br>327.0858,297.0754	[26]
123	isochlorogenic acid A	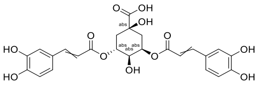	Pa	LJT	23.89	$[M-H]^-$	353.0883,335.0772,<br>173.0445,135.0440	[36]

124	reynoutrin	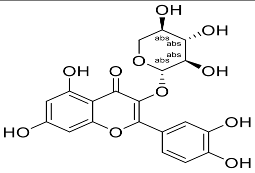	Fg	PCR	24.65	[M-H]$^-$	/	[27]
125	avicularin	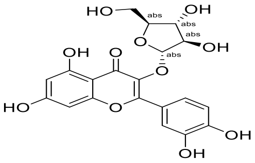	Fg	PCR	25.43	[M-H]$^-$	/	[44]
126	resveratroloside	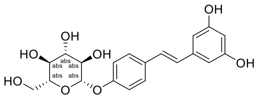	Op	PCR	25.46	[M+H]$^+$	229.0856,211.0759, 135.0440,107.0495	[27]
127	liquiritigenin 7,4'-diglucoside	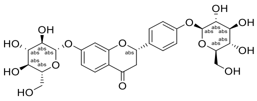	Fg	GR	25.61	[M+H]$^+$	538.0963,431.0979, 311.0434,287.0430	[43]
128	centauroside	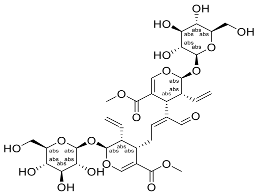	Ter	LJT	25.64	[M-H]$^-$	679.1150,525.1623, 458.1185,254.0573	[26]
129	3,4-dicaffeoylquinic acid	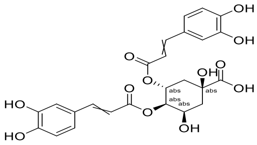	Pa	LJT	25.75	[M-H]$^-$	437.3583,353.0874, 191.0552,135.0440	[36]
130	herniarin	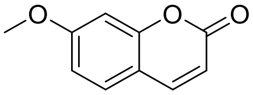	Oth	PLR	26.18	[M+H]$^+$	149.0597,145.0283, 117.0336,89.0390	[28]
131	catechin-5-*O*-*β*-*D*-glucopyranoside	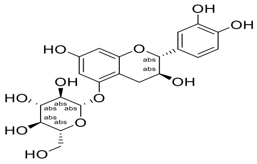	Fg	PCR	26.54	[M-H]$^-$	313.0739,289.0719, 191.0340,167.0340	[27]
132	vanillin	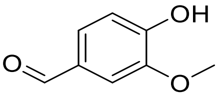	Op	PLR	26.58	[M+H]$^+$	131.9743,125.0597, 111.0443,93.0338	[28]
133	4,7-dihydroxyflavone 7-*D*-glucoside	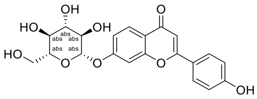	Fg	GR	26.84	[M+H]$^+$	338.5892,255.0647, 227.0695,199.0747	[35]
134	methyl chlorogenate	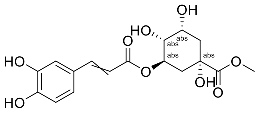	Pa	LJT	27.00	[M+H]$^+$	313.0666,285.0745, 207.0644,161.0596	[26]
135	ketologanin	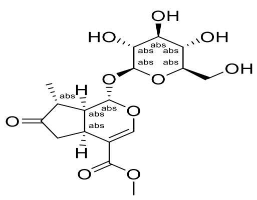	Ter	LJT	27.07	[M+H]$^+$	371.1681,324.1584, 225.0426,151.0388	[26]
136	naringin	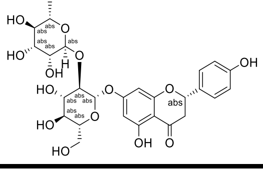	Fg	GR	27.96	[M+H]$^+$	449.1047,431.0979, 329.0610,311.0434	[35]

137	*(E)*-aldosecologanin	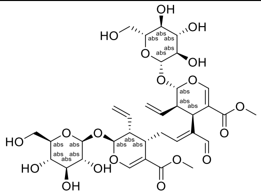	Ter	LJT	28.10	[M-H]$^{-}$	679.1150,595.2075,<br>525.1623,458.1185	[26]
138	dihydrocaffeic acid	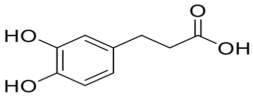	Pa	LJT	28.25	[M+H]$^{+}$	165.0545,151.0389,<br>123.0441,113.9639	[36]
139	*p*-coumaric acid	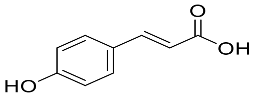	Pa	PLR	28.25	[M+H]$^{+}$	137.0597,133.0283,<br>109.0650,79.0547	[28]
140	secoxyloganin	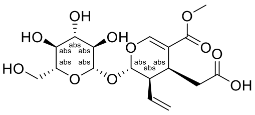	Ter	LJT	28.25	[M+H]$^{+}$	373.2119,309.2449,<br>165.0545,151.0389	[26]
141	benzoic acid	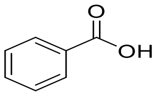	Oa	LJT	28.26	[M+H]$^{+}$	105.0450,95.0495,<br>67.0549	[41]
142	1,5-dicaffeoylquinic acid	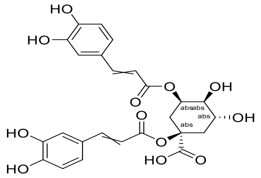	Pa	LJT	28.63	[M-H]$^{-}$	454.9042,353.0873,<br>191.0552,173.0446	[36]
143	vogeloside	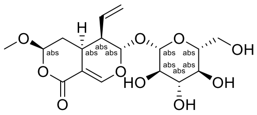	Ter	LJT	28.65	[M+H]$^{+}$	233.2362,195.0655,<br>151.0389,107.0495	[26]
144	3-*O*-caffeoylquinic acid methyl ester	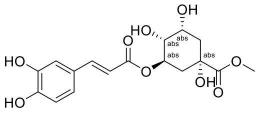	Pa	LJT	28.94	[M+H]$^{+}$	207.0649,177.0546,<br>148.0514,107.0857	[36]
145	quercitrin	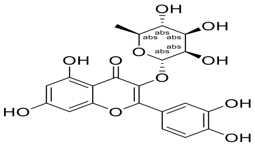	Fg	PCR	30.05	[M+H]$^{+}$<br>[M-H]$^{-}$	330.0535,287.0545,<br>269.0448,153.0181<br>403.1030,241.0501,<br>197.0599,174.9555	[27]
146	4-feruloylquinic acid	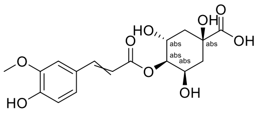	Pa	LJT	30.26	[M+H]$^{+}$	239.4636,207.0649,<br>177.0539,148.0516	[36]
147	naringenin	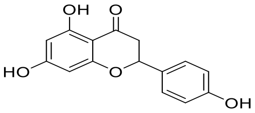	Fla	HE	31.32	[M-H]$^{-}$	230.0589,177.0189,<br>151.0026,119.0490	[45]
148	kuzubutenolide A	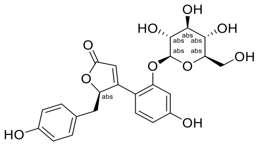	Fg	PLR	31.41	[M+H]$^{+}$	299.0909,253.0853,<br>193.0497,107.0494	[28]
149	pueroside A	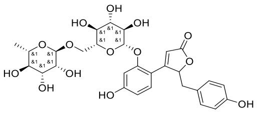	Fg	PLR	31.42	[M+H]$^{+}$	461.1439,376.1363,<br>299.0908,107.0494	[28]
150	epicatechin gallate	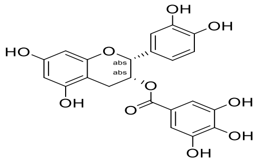	Fla	PCR	31.75	[M+H]$^{+}$	390.0869,291.0855,<br>273.0755,123.0441	[37]

151	garbanzol	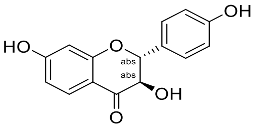	Fla	PLR	31.76	[M+H]+	242.4491,189.0543,<br>153.0180,123.0441	[28]
152	chrysoeriol 7-*O*-glucopyranoside	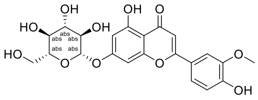	Fg	LJT	31.80	[M+H]+	445.1107,427.1008,<br>343.0803,313.0704	[26]
153	sophoraside A	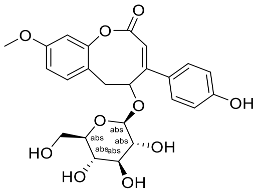	Fg	PLR	31.92	[M+H]+<br>[M-H]-	313.1061,267.1010,<br>253.0853,107.0494<br>377.9086,311.0924,<br>267.1024,252.0786	[28]
154	vitexin	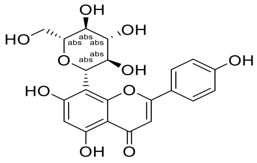	Fg	HE	32.25	[M+H]+<br>[M-H]-	415.1018,397.0909,<br>313.0698,283.0597<br>269.0453,240.0423,<br>225.0551,193.4129	[25]
155	5-*O*-coumaroylcaffeoylquinic acid	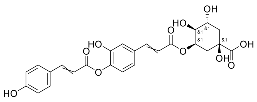	Pa	LJT	32.38	[M+H]+<br>[M-H]-	483.1254,320.0835,<br>255.0652,163.0388<br>431.0978,291.0275,<br>269.0454,240.0423	[38]
156	resveratrol	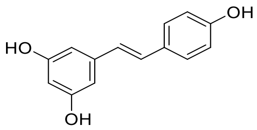	Op	PCR	32.64	[M+H]+	211.0747,183.0808,<br>135.0441,107.0494	[27]
157	ferulic acid	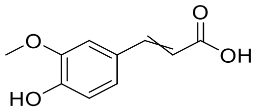	Pa	LJT	32.90	[M+H]+<br>[M-H]-	177.0544,163.0389,<br>138.0661,107.0494<br>165.0005,134.0361,<br>126.9024,102.9472	[36]
158	isoferulic acid	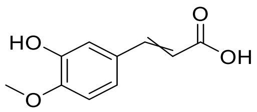	Pa	LJT	32.91	[M+H]+<br>[M-H]-	177.0544,163.0388,<br>149.0596,109.0287<br>161.0233,149.0236,<br>134.0363,121.0281	[36]
159	kudzusaponin A1	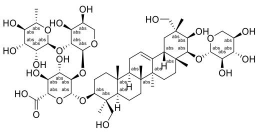	Ter	PLR	34.62	[M-H]-	1029.5265,763.3842<br>,603.3890,485.3619	[28]
160	hyperoside	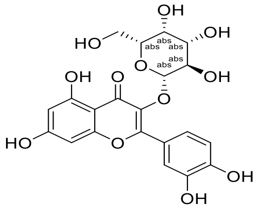	Fg	LJT	35.15	[M+H]+	447.1085,303.0492,<br>286.0449,257.0425	[26]
161	polygalin A	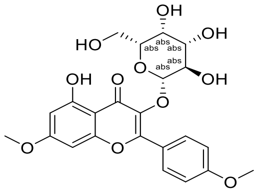	Fg	PCR	35.16	[M+H]+<br>[M-H]-	355.1165,315.0853,<br>271.0960,229.0856<br>267.0660,252.0424,<br>201.9968,132.0607	[27]
162	7-hydroxy-4-methoxy-5-methylcoumarin	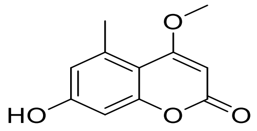	Op	PCR	35.24	[M+H]+	189.0544,161.0599,<br>150.0261,123.0807	[27]

163	glycitin-6''-*O*-xylosyl	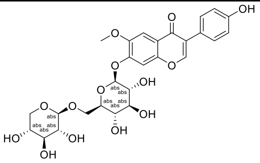	Fg	PLR	36.12	$[M+H]^+$	433.1124,337.0699,<br>313.0699,283.0596	[28]
164	cuspidatumin A	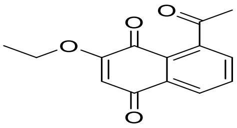	Oth	PCR	36.14	$[M+H]^+$<br>$[M-H]^-$	229.0854,161.0122,<br>121.0286,98.9757<br>225.1119,207.1026,<br>174.9554,146.9600	[27]
165	3,5-dicaffeoylquinic acid methyl ester	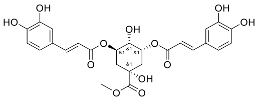	Pa	LJT	36.23	$[M+H]^+$	513.1385,369.1514,<br>283.0595,163.0388	[36]
166	rutin	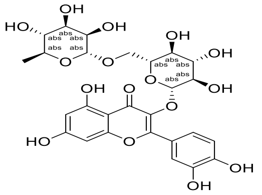	Fg	LJT	36.27	$[M+H]^+$	465.1010,303.0493,<br>257.0441,229.0495	[26]
167	taxifolin	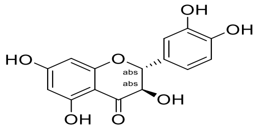	Fla	HE	36.27	$[M+H]^+$	287.1236,269.1125,<br>227.1023,191.0814	[45]
168	pueroside B	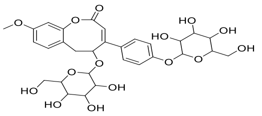	Fg	PLR	36.31	$[M+H]^+$	475.1591,313.1064,<br>267.1011,107.0494	[28]
169	pueroside C	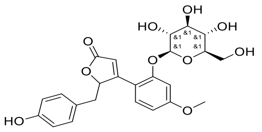	Fg	PLR	36.31	$[M+H]^+$	457.3117,313.1034,<br>249.1549,107.0486	[28]
170	macranthoidin B	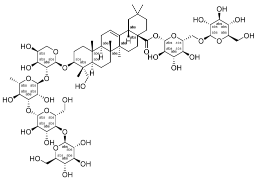	Ter	LJT	36.35	$[M+H]^+$<br>$[M-H]^-$	1075.5695,943.5251<br>,795.2725,633.2202<br>1073.5525,911.5010<br>,749.4481,603.3898	[26]
171	kudzusaponin SA2	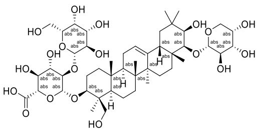	Ter	PLR	36.39	$[M+H]^+$	848.4162,763.4678,<br>679.2439,421.3453	[28]
172	macranthoidin A	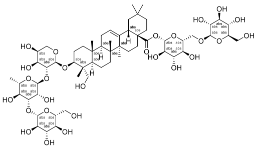	Ter	LJT	36.40	$[M+H]^+$<br>$[M-H]^-$	1076.5618,943.5206<br>,751.4630,603.2128<br>1189.5997,1073.553<br>4,911.5006,749.448<br>2	[26]
173	kudzusaponin SA4	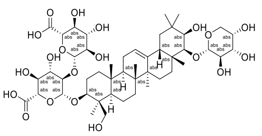	Ter	PLR	36.40	$[M+H]^+$	892.2470,764.6243,<br>615.3878,421.3457	[28]
174	saponin 1	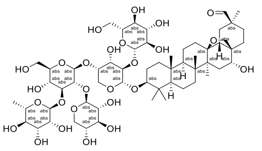	Ter	LJT	36.43	$[M+H]^+$<br>$[M-H]^-$	1075.5693,913.5162<br>,751.4610,603.2120<br>881.4901,749.4479,<br>603.3898,471.3479	[26]

175	24-hydroxy-licorice-saponin A3	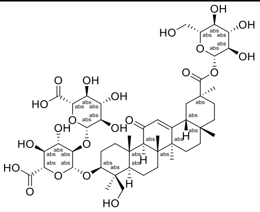	Ter	GR	36.45	[M+H]$^+$	825.4282,763.0059, 631.3789,469.3288	[35]
176	3,4-*O*-dicaffeoyl quinic acid methyl ester	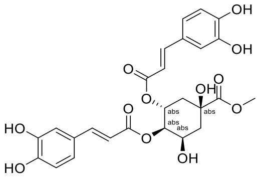	Pa	LJT	36.46	[M+H]$^+$<br>[M-H]$^-$	319.0808,271.0598, 177.0545,163.0388<br>443.6241,367.1035, 191.0554,135.1440	[36]
177	isoquercetin	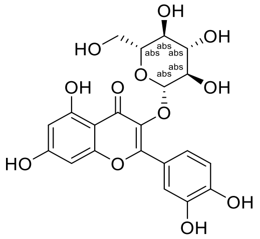	Fg	GR	36.47	[M+H]$^+$	303.0494,257.0439, 229.0495,153.0182	[35]
178	soyasaponin A3	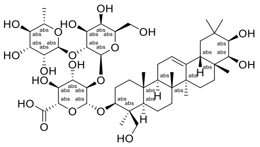	Ter	PLR	36.48	[M+H]$^+$<br>[M-H]$^-$	813.4605,439.3565, 141.0181,85.0289<br>911.5010,749.4482, 587.3950,471.3475	[28]
179	dipsacoside B	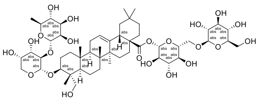	Ter	LJT	36.49	[M-H]$^-$	912.0020,749.4480, 585.3804,471.3478	[26]
180	kudzusaponin B1	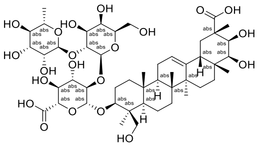	Ter	PLR	36.52	[M+H]$^+$<br>[M-H]$^-$	843.4330,681.3870, 469.3314,141.0181<br>926.4868,763.7924, 661.3583,503.3387	[28]
181	saponin 4	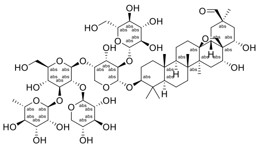	Ter	LJT	36.55	[M-2H]$^{-2}$	/	[26]
182	licoricesaponin A3	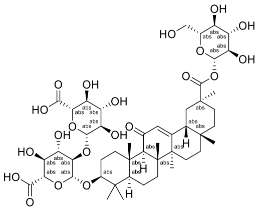	Ter	GR	36.57	[M+H]$^+$<br>[M-H]$^-$	809.4323,615.3887, 453.3356,189.1634<br>943.1790,821.3969, 645.3637,351.0566	[46]
183	neoliquiritin	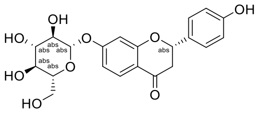	Fg	GR	36.58	[M+H]$^+$	315.0854,257.0803, 217.0483,124.0392	[47]
184	6''-*O*-malonyldaidzin	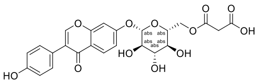	Fg	PLR	36.58	[M+H]$^+$	480.9303,392.3837, 255.0647,199.0751	[28]
185	*(2E)*-1-(2,3-dihydroxy-4-methoxyphenyl)-3-(4-hydroxyphenyl)-2-propen-1-one	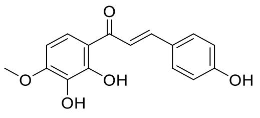	Fla	GR	36.58	[M+H]$^+$<br>[M-H]$^-$	245.0804,207.0649, 193.0492,121.0285<br>270.0532,177.0185, 150.0311,108.0206	[35]
186	loniceroside D	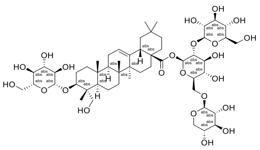	Ter	LJT	36.59	[M+H]$^+$<br>[M-H]$^-$	1033.7538,945.5055 ,783.4556,421.3455<br>1071.5394,943.4768 ,882.4898,763.4315	[26]

187	akebia saponin D	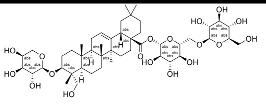	Ter	LJT	36.60	[M+H]$^+$	767.4589,635.4064,<br>437.3408,189.1637	[38]
188	kudzusaponin A2	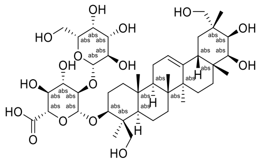	Ter	PLR	36.61	[M+H]$^+$<br>[M-H]$^-$	764.7684,649.3955,<br>455.3521,269.0806<br>763.3452,677.4987,<br>516.0891,333.8636	[28]
189	isorhamnetin 3-*O*-glucopyranoside	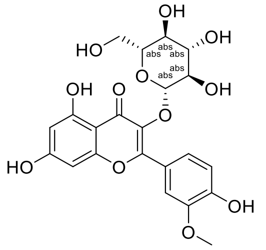	Fg	LJT	36.62	[M+H]$^+$	397.5868,317.0649,<br>274.0458,120.0809	[26]
190	astragalin	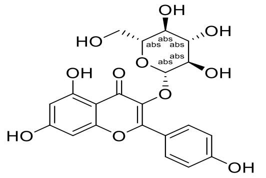	Fg	LJT	36.62	[M+H]$^+$<br>[M-H]$^-$	409.0180,346.9581,<br>287.0544,252.9790<br>316.5085,284.0322,<br>255.0294,227.0343	[26]
191	7,4'-dihydroxyflavone	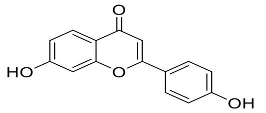	Fla	GR	36.63	[M+H]$^+$<br>[M-H]$^-$	227.0696,199.0752,<br>137.0234,91.0546<br>224.0470,208.0522,<br>135.0076,91.0174	[43]
192	isorhamentin 3-*O*-rutinoside	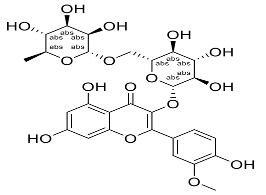	Fg	LJT	36.66	[M+H]$^+$<br>[M-H]$^-$	479.1161,317.0652,<br>302.0414,85.0289<br>527.7530,415.1031,<br>252.0425,223.0404	[26]
193	4'-methoxypuerarin	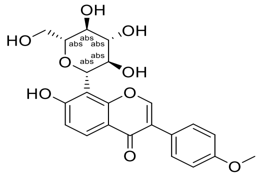	Fg	PLR	36.67	[M+H]$^+$	395.1120,365.1009,<br>311.0910,271.0595	[28]
194	4,5-*O*-dicaffeoyl quinic acid methyl ester	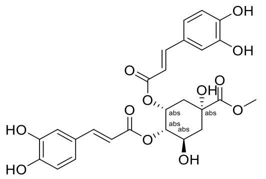	Fg	LJT	36.67	[M-H]$^-$	483.1268,463.2749,<br>367.1032,253.0501	[36]
195	quercetin 3-*O*-arabinoside	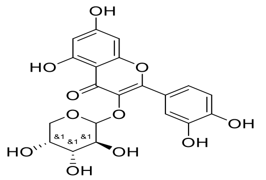	Fg	PCR	36.67	[M+H]$^+$	303.0501,271.0596,<br>153.0180,121.0280	[27]
196	loniceroside A	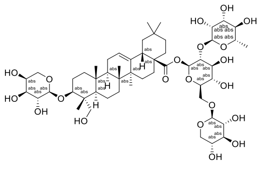	Ter	LJT	36.69	[M-H]$^-$	1025.5223,763.3167<br>,709.8038,532.3125	[26]
197	rhein	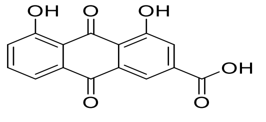	Ant	PCR	36.71	[M+H]$^+$<br>[M-H]$^-$	269.0440,257.0428,<br>151.0385,121.0283<br>268.0373,217.0500,<br>175.0391,133.0284	[27]
198	3,4,5-tricaffeoylquinic		Pa	LJT	36.72	[M+H]$^+$	499.1226,322.2479,	[38]

	acid	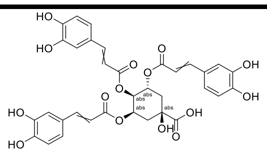				[M-H]-	163.0387,135.0440<br>515.1179,353.0875,<br>173.0446,135.0440	
199	choerospondin	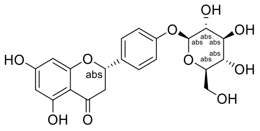	Fg	GR	36.74	[M+H]+	303.0501,271.0596,<br>231.0647,153.0180	[39]
200	4,5-dicaffeoylquinic acid	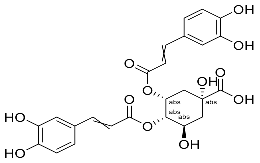	Pa	LJT	36.75	[M+H]+	499.1223,453.8935,<br>269.0803,163.0387	[41]
201	pollenitin B	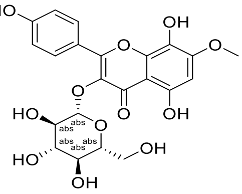	Fg	HE	36.76	[M+H]+	412.8673,317.0651,<br>302.0415,274.0472	[32]
202	medicarpin 3-*O*-glucoside	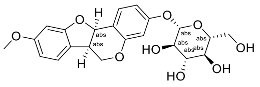	Fg	GR	36.77	[M+H]+	312.0939,271.0596,<br>214.2812,153.0182	[46]
203	lonfuranacid A	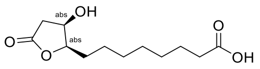	Oa	LJT	36.77	[M+H]+	229.0853,189.1119,<br>125.0962,97.1015	[41]
204	questin	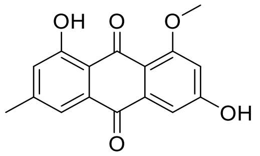	Ant	PCR	36.78	[M+H]+	270.0518,253.0490,<br>242.0574,153.0179	[27]
205	tricin 7-*O*-glucoside	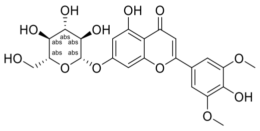	Fg	LJT	36.78	[M+H]+	331.0807,315.0493,<br>287.0537,270.0518	[26]
206	tectoridin	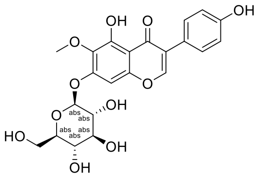	Fg	PLR	36.79	[M+H]+	301.0700,286.0467,<br>258.0517,153.0181	[28]
207	liquiritigenin	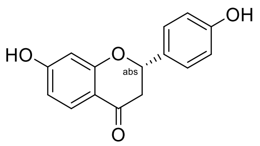	Fg	GR	36.80	[M+H]+<br>[M-H]-	239.0705,211.0756,<br>147.0439,137.0232<br>209.0605,153.0183,<br>135.0077,119.0490	[35]
208	subproside V	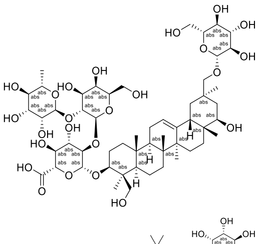	Ter	PLR	36.80	[M-H]-	1073.5519,911.5007<br>,749.4478,603.3897	[28]
209	loniceroside E	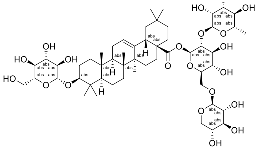	Ter	LJT	36.81	[M-H]-	1039.5623,849.4960<br>,763.3219,413.0908	[26]

210	kudzusaponin A5	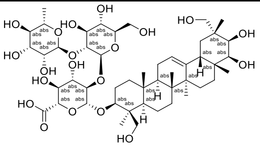	Ter	PLR	36.82	$[M+H]^+$	829.4545,764.4423,<br>667.4020,455.3510	[28]
211	torachrysone	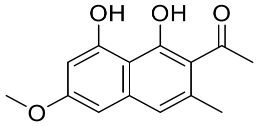	Op	PCR	36.82	$[M+H]^+$<br>$[M-H]^-$	229.0856,214.0621,<br>201.0907,198.0673<br>230.0579,215.0343,<br>202.0625,159.0440	[27]
212	macranthoside B	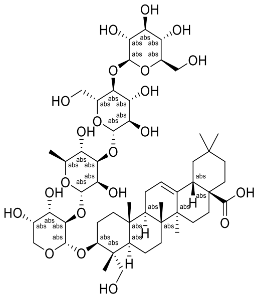	Ter	LJT	36.83	$[M+H]^+$	943.5235,781.4703,<br>619.4197,437.3409	[26]
213	glycyroside	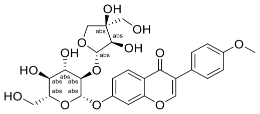	Fg	GR	36.83	$[M+H]^+$<br>$[M-H]^-$	431.1331,413.1223,<br>311.0907,281.0803<br>523.2799,339.0867,<br>309.0767,266.0582	[35]
214	physcion	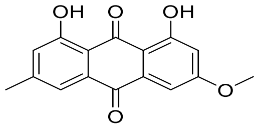	Ant	PCR	36.84	$[M+H]^+$<br>$[M-H]^-$	270.0518,242.0567,<br>189.4096,113.0597<br>268.0375,240.0419,<br>211.0391,184.0518	[27]
215	afzelin	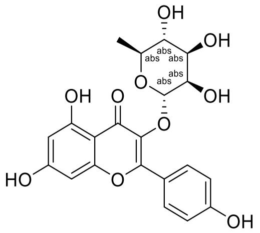	Fg	GR	36.88	$[M+H]^+$<br>$[M-H]^-$	418.8996,271.0596,<br>243.0644,215.0699<br>269.0454,240.0424,<br>225.0552,152.9942	[47]
216	kudzusaponin SA3	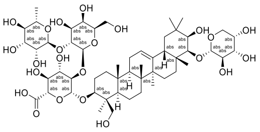	Ter	PLR	36.89	$[M+H]^+$<br>$[M-H]^-$	929.5117,767.4597,<br>635.4111,437.3414<br>1043.5427,881.4904<br>,749.4480,603.3900	[28]
217	*22β*-acetoxyglycyrrhizin	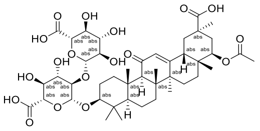	Ter	GR	36.91	$[M+H]^+$	705.3826,511.3415,<br>451.3196,107.0859	[35]
218	kudzusaponin C1	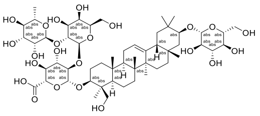	Ter	PLR	36.93	$[M+H]^+$	959.5132,797.498,<br>603.4246,423.3591	[28]
219	questinol	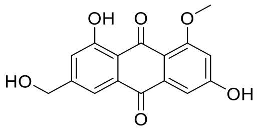	Ant	PCR	36.94	$[M+H]^+$	286.0460,269.0439,<br>167.0338,134.0362	[27]
220	3'-methoxydaidzin	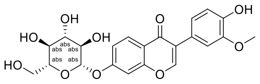	Fg	PLR	36.96	$[M+H]^+$	384.1155,327.0859,<br>285.0752,229.0857	[28]
221	loniceroside B	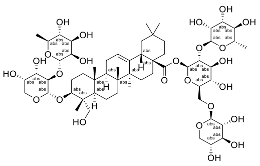	Ter	LJT	36.96	$[M+H]^+$	817.3367,763.4253,<br>619.4137,437.3397	[26]

222	citreorosein	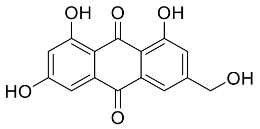	Ant	PCR	36.97	[M+H]+<br>[M-H]-	271.0596,269.0443,<br>259.0960,217.0491<br>268.0367,257.0461,<br>196.0532,133.0284	[27]
223	herbacetin	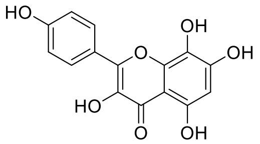	Fla	HE	37.01	[M+H]+<br>[M-H]-	286.0430,257.0442,<br>229.0497,153.0181<br>284.0315,273.0407,<br>178.9976,151.0026	[31]
224	betulonic acid	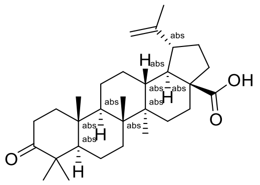	Ter	GR	37.02	[M+H]+	409.3467,388.4104,<br>203.1793,189.1635	[39]
225	kudzusaponin A3	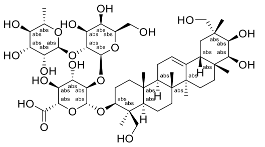	Ter	PLR	37.05	[M+H]+	829.4545,667.4020,<br>455.3510,141.0181	[28]
226	ononin	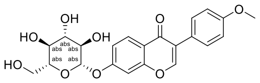	Fg	PLR	37.08	[M+H]+	269.0805,254.0569,<br>213.0910,107.0494	[28]
227	kaempferol 3-*O*-rutinoside	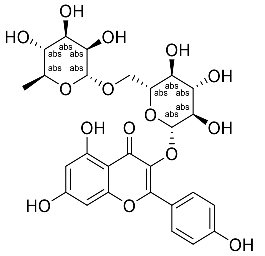	Fg	LJT	37.11	[M+H]+<br>[M+H]+	525.0455,433.1108,<br>287.0544,271.0596<br>525.0455,433.1108,<br>287.0544,271.0596	[26]
228	isobavachalcone	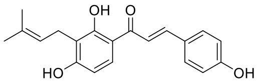	Fla	GR	37.11	[M+H]+	309.0781,285.0754,<br>189.0906,95.0163	[48]
229	liqcoumarin	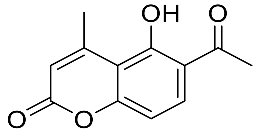	Op	GR	37.18	[M+H]+	201.0910,174.0674,<br>133.1012,105.0702	[42]
230	isokaempferide	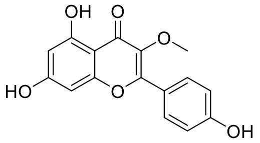	Fla	GR	37.19	[M+H]+	283.0596,255.0636,<br>227.0698,123.1169	[46]
231	uralsaponin F	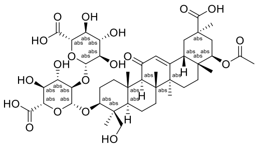	Ter	GR	37.21	[M+H]+	763.6343,679.2714,<br>527.3329,334.7203	[39]
232	daidzein 4',7-diglucoside	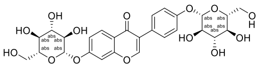	Fg	PLR	37.25	[M+H]+	503.0054,447.1283,<br>285.0753,229.0858	[28]
233	liquoric acid	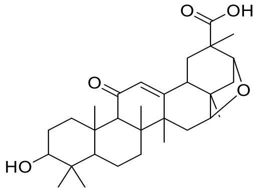	Ter	GR	37.26	[M+H]+	323.1276,255.0648,<br>199.0751,163.0385	[49]
234	isoorientin	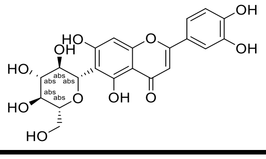	Fg	PLR	37.27	[M+H]+	330.0535,287.0545,<br>153.0181,135.0439	[28]

235	isorhodoptilometrin	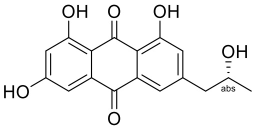	Ant	PCR	37.31	$[M+H]^+$<br>$[M-H]^-$	300.0623,272.0670, 153.0185,95.0858<br>298.0479,270.0529, 227.0343,183.0454	[27]
236	chrysophanol	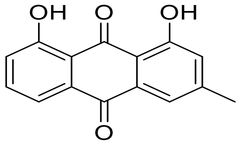	Ant	PCR	37.34	$[M+H]^+$	237.0549,227.0692, 199.0751,187.0725	[27]
237	licoricesaponin G2	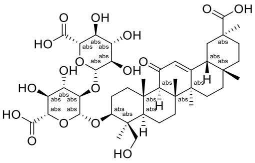	Ter	GR	37.34	$[M+H]^+$<br>$[M-H]^-$	663.3734,487.3410, 469.3306,141.0181<br>763.7259,724.4032, 351.0571,193.0345	[46]
238	kudzusaponin SA1	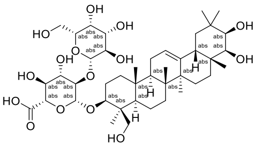	Ter	PLR	37.35	$[M+H]^+$<br>$[M-H]^-$	764.7317,439.3548, 141.0181,95.0860<br>765.4406,603.3923, 432.7037,283.0584	[28]
239	1,4-dicaffeoylquinic acid	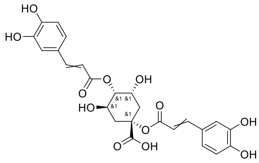	Pa	LJT	37.38	$[M+H]^+$	460.9336,414.0990, 269.0804,213.0906	[36]
240	ethyl caffeate	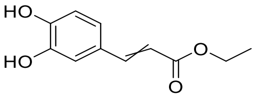	Pa	LJT	37.38	$[M+H]^+$<br>$[M-H]^-$	163.0388,145.1011, 135.0441,117.0337<br>179.0341,161.0233, 135.0441,121.0284	[41]
241	apigenin 7-glucoside	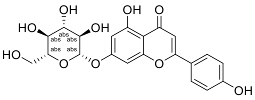	Fg	HE	37.41	$[M+H]^+$<br>$[M-H]^-$	379.0797,337.0701, 313.0699,271.0596<br>311.0562,269.0453, 225.0554,152.9949	[25]
242	daidzin	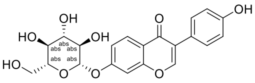	Fg	PLR	37.42	$[M+H]^+$	387.2100,297.0747, 255.0648,199.0753	[28]
243	2,5-dimethyl-7-hydroxychromenone	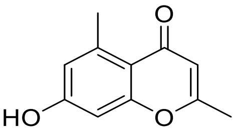	Op	PCR	37.46	$[M+H]^+$	151.0387,131.0856, 107.0861,95.0860	[27]
244	prunetin	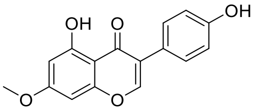	Fla	LJT	37.46	$[M+H]^+$<br>$[M-H]^-$	253.0489,242.0572, 211.0750,151.0390<br>268.0380,240.0423, 197.0600,168.0650	[38]
245	glabrolide	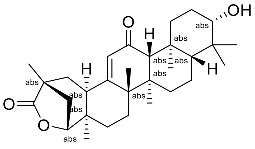	Ter	GR	37.47	$[M+H]^+$	233.1540,175.1479, 135.1167,107.0858	[46]
246	pinocembrin	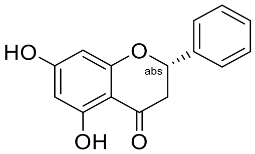	Fla	GR	37.53	$[M+H]^+$	239.0701,229.0850, 211.0752,147.0440	[50]
247	polygonin B	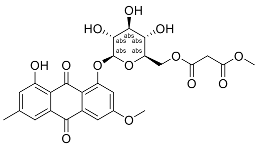	Ant	PCR	37.54	$[M+H]^+$	299.0909,284.0674, 239.0702,163.0385	[40]

248	glycyrrhizic acid	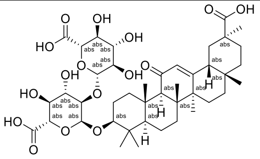	Ter	GR	37.56	[M+H]+<br>[M-H]-	647.3788,471.3457, 453.3356<br>763.8063,469.3315, 351.0569,193.0347	[39]
249	echinatin	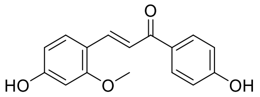	Fla	GR	37.56	[M+H]+<br>[M-H]-	254.2115,147.0438, 137.0596,123.0441<br>251.0708,225.0552, 151.0030,119.0490	[35]
250	hesperetin	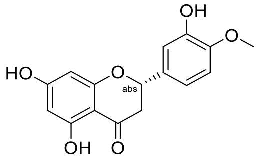	Fla	PCR	37.62	[M+H]+<br>[M-H]-	258.0517,153.0183, 106.0866,88.0762<br>273.0774,255.0296, 230.0580,183.0447	[27]
251	*(S)*-naringenin	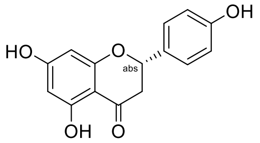	Fla	GR	37.62	[M+H]+	189.0543,153.0180, 123.0441	[39]
252	puerol B	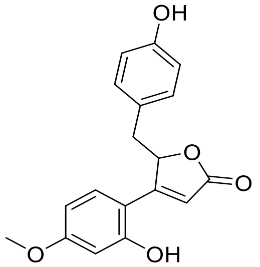	Fla	PLR	37.64	[M+H]+<br>[M-H]-	267.1011,253.0854, 107.0495<br>296.0689,267.1026, 252.0789,161.0233	[28]
253	coumestrol	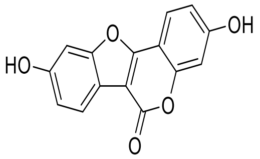	Op	PLR	37.72	[M+H]+<br>[M-H]-	254.0572,241.0492, 213.0543,185.0595<br>251.0709,225.0550, 181.0649,151.0026	[28]
254	polygonin A	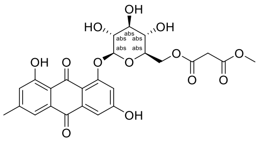	Ant	PCR	37.73	[M+H]+<br>[M-H]-	488.1885,360.1438, 285.0753,270.0517<br>341.9318,253.0502, 229.0135,191.0555	[40]
255	tricin	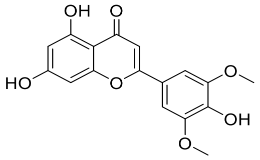	Fla	HE	37.73	[M+H]+<br>[M-H]-	315.0492,302.0406, 270.0519,73.0291<br>271.0247,211.1332, 171.1017	[31]
256	neobavaisoflavone	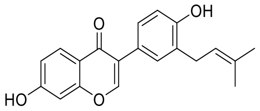	Fla	PLR	37.76	[M+H]+	308.0663,267.0647, 255.0648,239.0698	[28]
257	biochanin	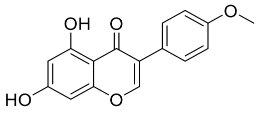	Fla	GR	37.76	[M+H]+<br>[M-H]-	270.0516,253.0491, 225.0540,137.0233<br>268.0377,240.0423, 224.0474,135.0075	[35]
258	gancaonin V	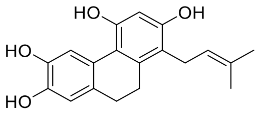	Op	GR	37.76	[M+H]+<br>[M-H]-	281.1162,244.0359, 153.0181<br>296.0687,267.1025, 252.0789,161.0232	[42]
259	6,7-dimethoxycoumarin	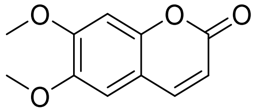	Oth	PLR	37.76	[M+H]+	189.1636,175.0388, 148.0517,91.0547	[28]

260	puerol A	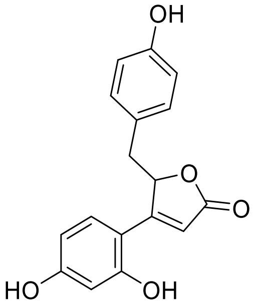	Fla	PLR	37.78	$[M+H]^+$<br>$[M-H]^-$	284.0674,256.0726, 239.0698,95.0163<br>281.0457,256.0376, 239.0346,151.0025	[28]
261	biapigenin	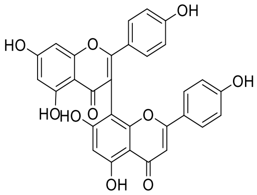	Fla	PCR	37.78	$[M+H]^+$<br>$[M-H]^-$	522.9716,387.0856, 286.0465,184.0731<br>521.0623,417.0622, 375.0506,331.0608	[51]
262	2-methoxy-6-acetyl-7-methyljuglone	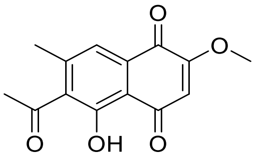	Op	PCR	37.79	$[M+H]^+$<br>$[M-H]^-$	243.0648,215.0699, 200.0466,187.0754<br>243.1414,231.0657, 216.0422,188.0471	[27]
263	kudzusaponin SB1	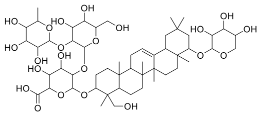	Ter	PLR	37.79	$[M+H]^+$<br>$[M-H]^-$	943.5235,751.4603, 437.3405,189.1636<br>911.5006,749.4489, 603.3903,471.3480	[28]
264	diosmetin	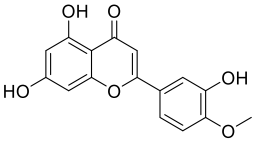	Fla	HE	37.81	$[M+H]^+$<br>$[M-H]^-$	286.0468,258.0523, 241.0491,88.0762<br>284.0325,256.0372, 227.0344,151.0030	[45]
265	*(-)-epi*afzelechin	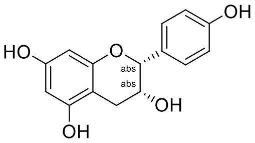	Fla	HE	37.82	$[M+H]^+$<br>$[M-H]^-$	257.0795,217.0492, 189.0544,107.0495<br>258.0532,230.0579, 215.0343,135.0076	[25]
266	licoricesaponin E2	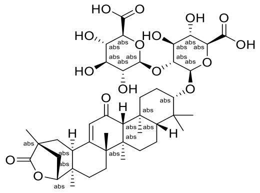	Ter	GR	37.85	$[M+H]^+$	764.7926,451.3198, 173.1327,121.1012	[46]
267	methyl glycyrrhizate	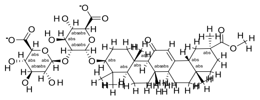	Ter	GR	37.86	$[M+H]^+$	764.7705,663.3716, 469.3308,141.0181	[39]
268	licoisoflavanone	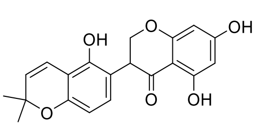	Fla	GR	37.88	$[M+H]^+$<br>$[M-H]^-$	337.1062,299.0546, 179.0337,123.0441<br>335.0921,312.0276, 217.0863,189.0913	[35]
269	3-methoxyherbacetin	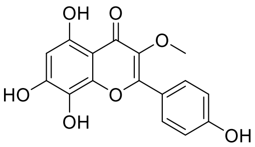	Fla	HE	37.89	$[M+H]^+$<br>$[M-H]^-$	302.0411,237.0383, 153.0181,127.0391<br>300.0272,272.0323, 188.0482,112.9845	[25]
270	erybacin B	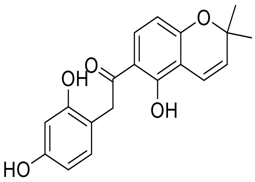	Op	GR	37.89	$[M+H]^+$<br>$[M-H]^-$	271.0597,117.0367, 95.0163,77.0059<br>309.2072,297.0051, 197.1174,171.1016	[35]

271	soyasaponin I	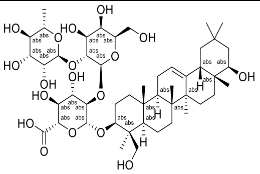	Ter	PLR	37.91	[M+H]+<br>[M-H]-	797.4680,764.6400, 423.3611,85.0289<br>912.5775,763.8070, 615.3933,438.3518	[28]
272	licoricesaponin B2	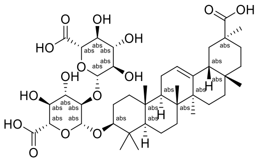	Ter	GR	37.93	[M+H]+<br>[M-H]-	633.3988,439.3564, 285.2223,107.0859<br>763.8304,520.9705, 351.0565,193.0345	[46]
273	ephedrannin B	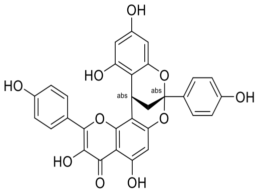	Fla	HE	37.94	[M+H]+<br>[M-H]-	415.0806,389.1013, 171.0287,153.0181<br>521.2609,507.2097, 396.8802,266.9637	[25]
274	medicarpin	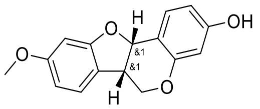	Fla	GR	37.97	[M+H]+	253.0497,229.0855, 197.0594,121.0285	[52]
275	kaempferol	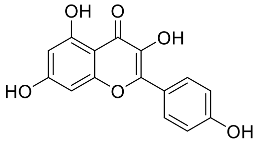	Fla	LJT	37.99	[M+H]+<br>[M-H]-	271.0556,254.0524, 226.0577,153.0181<br>268.0364,257.0451, 241.0497,211.0396	[41]
276	glyasperin D	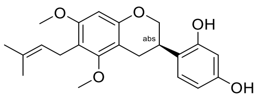	Fla	GR	38.04	[M+H]+<br>[M-H]-	315.1218,303.1219, 167.0701,123.0441<br>/	[35]
277	isoliquiritigenin	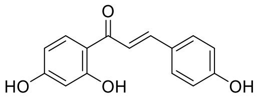	Fla	GR	38.09	[M+H]+<br>[M-H]-	239.0698,211.0755, 147.0440,137.0232<br>153.0182,135.0077, 119.0489,91.0175	[35]
278	3'-hydroxydaidzein	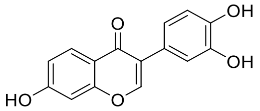	Fla	PLR	38.10	[M+H]+	253.0492,243.0647, 215.0702,153.0180	[28]
279	kaikasaponin III	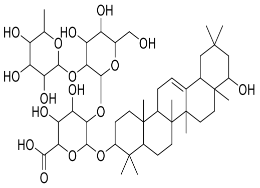	Ter	PLR	38.15	[M+H]+	767.4596,635.4124, 437.3406,203.1794	[28]
280	3,4,3',4'-tetrahydroxychalcone	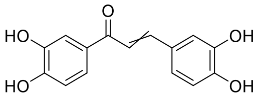	Fla	GR	38.16	[M+H]+	245.0811,171.0285, 153.0181,123.0442	[35]
281	araboglycyrrhizin	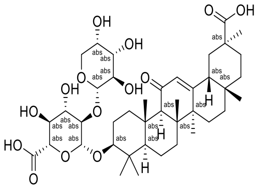	Ter	GR	38.21	[M+H]+	/	[35]
282	macranthoside A	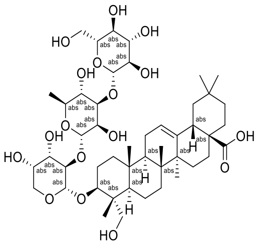	Ter	LJT	38.22	[M+H]+<br>[M-H]-	781.4694,617.4044, 423.3610,141.0180<br>749.4489,603.3895, 471.3479,423.3271	[26]

283	hydnocarpin	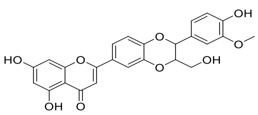	Fla	LJT	38.23	[M+H]+<br>[M-H]-	447.1065,286.0468, 257.0440,147.0438<br>447.2420,285.0402, 255.0293,208.9755	[26]
284	puerariafuran	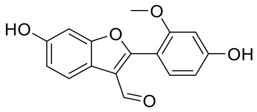	Fla	PLR	38.24	[M+H]+	270.0512,253.0493, 242.0569,211.0754	[28]
285	vestitol	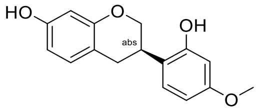	Fla	GR	38.29	[M+H]+	255.1017,227.1794, 137.0233,121.0285	[53]
286	homobutein	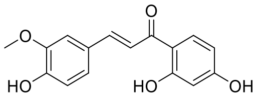	Fla	GR	38.31	[M+H]+	269.0440,241.0491, 185.0592,151.0389	[54]
287	glycycoumarin	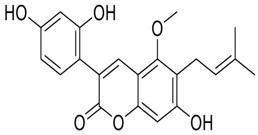	Fla	GR	38.34	[M+H]+	351.1228,297.0746, 193.0494,165.0545	[55]
288	licoricesaponin J2	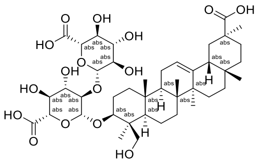	Ter	GR	38.34	[M+H]+<br>[M-H]-	764.8267,455.3507, 189.1634,141.0181<br>763.2627,473.1696, 351.0565,193.0342	[35]
289	licoricesaponin C2	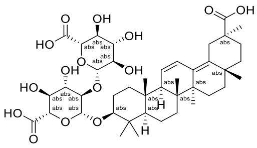	Ter	GR	38.35	[M+H]+<br>[M-H]-	764.8302,678.4443, 631.3784,437.3406<br>763.3167,453.3408, 351.0559,193.0349	[46]
290	3'-hydroxy-4'-*O*-methylglabridin	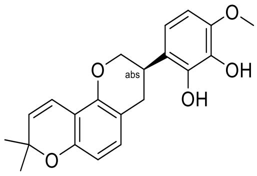	Fla	GR	38.39	[M-H]-	/	[35]
291	blumenol A	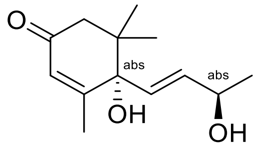	Ter	PLR	38.39	[M+H]+	210.1245,167.9932, 114.0913,95.0860	[28]
292	dihydrodaidzein	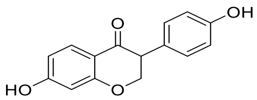	Fla	PLR	38.40	[M+H]+	239.0690,229.0851, 211.0744,147.0439	[28]
293	formononetin	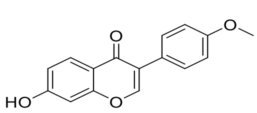	Fla	GR	38.44	[M+H]+<br>[M-H]-	254.0567,213.0907, 118.0414,95.0859<br>252.0423,225.0553, 195.0443,132.0204	[35]
294	lupiwighteone	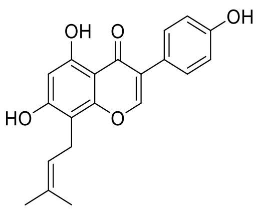	Fla	GR	38.44	[M+H]+	322.2484,283.0594, 271.0597,209.1646	[35]
295	quercetin	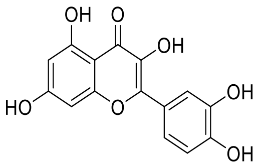	Fla	LJT	38.48	[M+H]+<br>[M-H]-	285.0393,257.0439, 229.0493,153.0181<br>283.0246,255.0298, 227.0342,138.0312	[26]

296	glycyuralin E	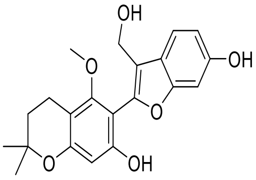	Fla	GR	38.52	[M+H]$^+$<br>[M-H]$^-$	353.1372,339.1213,<br>285.0749,167.0695<br>311.0558,229.0865,<br>206.0213,139.0390	[35]
297	estradiol	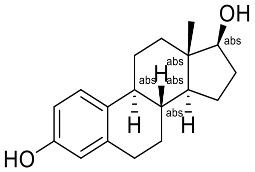	Ter	ASA	38.53	[M+H]$^+$	255.1007,248.4772,<br>153.0180,119.0856	[33]
298	licoflavone A	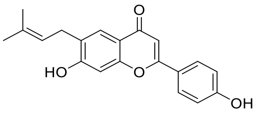	Fla	GR	38.57	[M+H]$^+$	280.0719,267.0648,<br>254.0570,239.0700	[35]
299	irisolidone	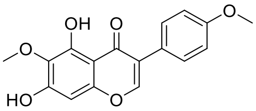	Fla	PLR	38.67	[M+H]$^+$<br>[M-H]$^-$	297.0751,226.0619,<br>199.0751,153.0182<br>295.0610,270.0479,<br>224.0468,167.2795	[28]
300	1-methoxyphaseollidin	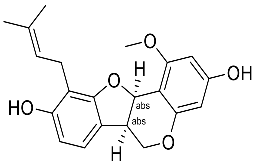	Fla	GR	38.68	[M+H]$^+$<br>[M-H]$^-$	299.0548,221.1169,<br>165.0546,123.0441<br>338.1162,292.0359,<br>253.0505,150.0311	[52]
301	cupressuflavone	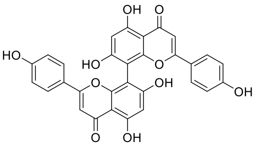	Fla	LJT	38.69	[M+H]$^+$<br>[M-H]$^-$	497.0887,403.0439,<br>377.0645,335.0543<br>521.2611,505.2242,<br>375.0520,266.9636	[38]
302	licoarylcoumarin	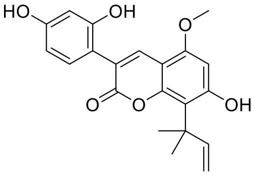	Fla	GR	38.69	[M+H]$^+$	313.0699,271.0596,<br>243.0647,147.0439	[54]
303	isoformononetin	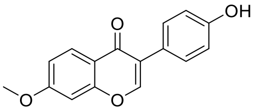	Fla	PLR	38.71	[M+H]$^+$<br>[M-H]$^-$	251.0697,241.0828,<br>237.0537,107.0855<br>252.0424,241.0503,<br>197.0604,96.9588	[28]
304	kakkasaponin I	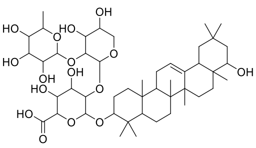	Ter	PLR	38.72	[M-H]$^-$	877.5569,763.6689,<br>678.9240,509.4025	[28]
305	*β*-amyrone	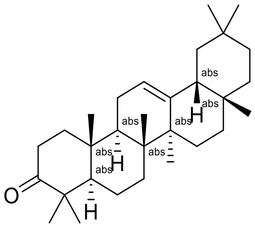	Ter	PLR	38.76	[M+H]$^+$	/	[28]
306	tuberosin	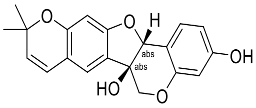	Fla	PLR	38.82	[M-H]$^-$	309.0397,281.0454,<br>254.0585,203.1068	[28]
307	glicophenone	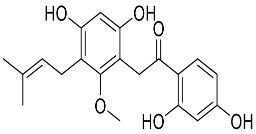	Op	GR	38.83	[M+H]$^+$<br>[M-H]$^-$	301.0710,283.0596,<br>175.0389,153.0545<br>247.0974,232.0737,<br>189.0186,109.0282	[35]

308	7,4'-dihydroxy-3'-methoxyisoflavan	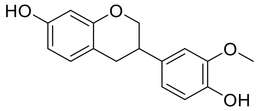	Fla	GR	38.83	[M+H]$^{+}$<br>[M-H]$^{-}$	245.1898,163.0750,137.0596,123.0442<br>241.0499,225.0550,197.0596,181.0652	[35]
309	2',3'-dihydro-7,7'-dihydroxy-5'-methoxy-2',2'-dimethyl[3,6'-bi-4H-benzopyran]-4-one	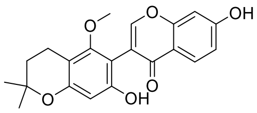	Fla	GR	38.89	[M+H]$^{+}$<br>[M-H]$^{-}$	313.0699,285.0752,270.0518,243.0648<br>337.0717,309.0403,256.0376,203.0708	[35]
310	3,4-didehydroglabridin	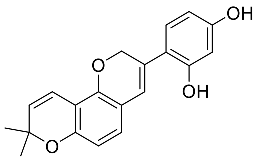	Fla	GR	38.98	[M+H]$^{+}$<br>[M-H]$^{-}$	267.0648,255.0647,239.0698,95.0163<br>277.0503,265.0505,252.0424,149.0598	[35]
311	glyasperin C	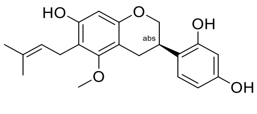	Fla	GR	38.98	[M+H]$^{+}$<br>[M-H]$^{-}$	301.1063,221.1165,165.0546,123.0441<br>298.0483,229.0865,174.0313,125.0232	[52]
312	neouralenol	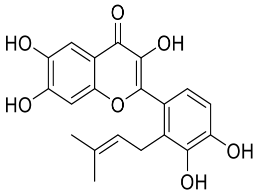	Fla	GR	39.01	[M+H]$^{+}$<br>[M-H]$^{-}$	315.0856,268.2631,183.0287,165.0181<br>351.0870,310.0444,283.0975,193.0135	[56]
313	phaseol	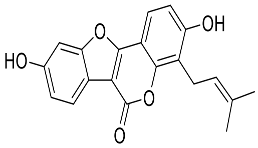	Fla	ASA	39.05	[M+H]$^{+}$	319.0956,283.0596,255.0646,163.0388	[57]
314	eurycarpin A	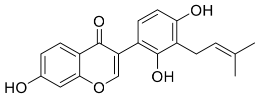	Fla	GR	39.08	[M+H]$^{+}$<br>[M-H]$^{-}$	322.2490,293.0592,163.0388,114.0915<br>293.1182,268.0376,224.0470,135.0077	[35]
315	glyurallin A	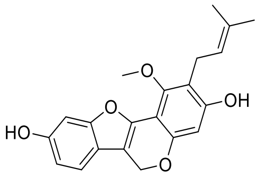	Fla	GR	39.10	[M-H]$^{-}$	335.0564,323.0929,308.0317,191.0711	[58]
316	sophoraisoflavone A	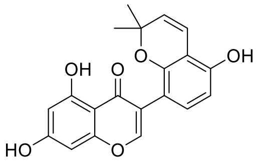	Fla	GR	39.10	[M+H]$^{+}$	335.0906,325.1064,283.02599,191.0343	[35]
317	licocoumarone	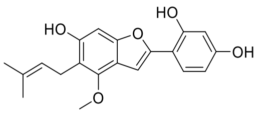	Op	GR	39.11	[M+H]$^{+}$<br>[M-H]$^{-}$	323.1265,267.0648,209.1646,114.0915<br>296.0677,268.0377,219.0656,119.0490	[35]
318	dehydrovomifoliol	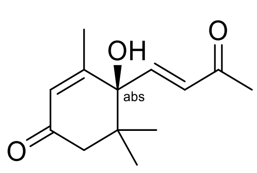	Oth	PLR	39.18	[M+H]$^{+}$<br>[M-H]$^{-}$	135.1167,107.0858,81.0704<br>205.1224,164.0829,148.0516,118.5610	[28]
319	fallacinol	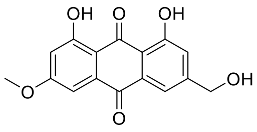	Ant	PCR	39.20	[M+H]$^{+}$<br>[M-H]$^{-}$	283.0598,269.0440,227.0701,199.0752<br>284.0317,255.0649,240.0422,212.0468	[27]

320	genkwanin	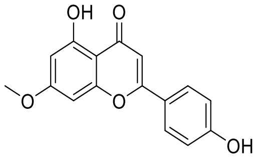	Fla	HE	39.23	$[M+H]^+$<br>$[M-H]^-$	270.0519,253.0494,<br>225.0542,137.0233<br>268.0378,240.0423,<br>186.6367,118.3947	[45]
321	kanzonol U	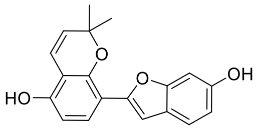	Op	GR	39.25	$[M+H]^+$	/	[48]
322	2,3-dehydrokievitone	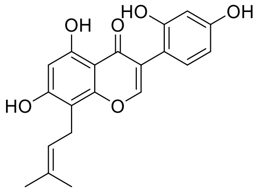	Fla	GR	39.26	$[M+H]^+$<br>$[M-H]^-$	337.1066,229.0854,<br>179.0338,123.0442<br>284.0319,243.1021,<br>216.0419,201.0915	[48]
323	2,3,4-trimethyl-5-phenyloxazolidine	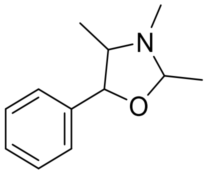	Alk	HE	39.28	$[M+H]^+$	133.1011,119.0493,<br>91.0547	[34]
324	pratensein	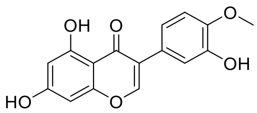	Fla	GR	39.29	$[M+H]^+$	283.0598,269.0440,<br>227.0701,199.0752	[54]
325	lupenone	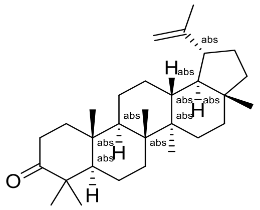	Ter	PLR	39.32	$[M+H]^+$	/	[28]
326	corylifol B	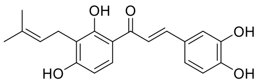	Fla	GR	39.35	$[M+H]^+$<br>$[M-H]^-$	267.0648,209.1646,<br>114.0916<br>269.0453,233.0818,<br>187.1117,167.0340	[35]
327	4-*O*-methylglabridin	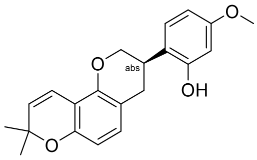	Fla	GR	39.36	$[M+H]^+$	322.2483,209.1644,<br>114.0916,95.0163	[59]
328	luteone	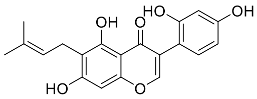	Fla	GR	39.43	$[M+H]^+$<br>$[M-H]^-$	338.3415,299.0540,<br>267.0284,239.0334<br>257.0063,227.0702,<br>165.0179,125.0232	[54]
329	glyinflanin H	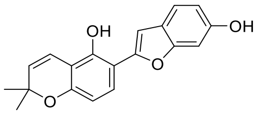	Fla	GR	39.43	$[M+H]^+$	291.1940,223.0596,<br>113.0600,95.0163	[48]
330	butyl octyl phthalate	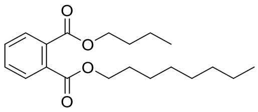	Oth	HE	39.44	$[M-H]^-$	293.0450,281.0451,<br>252.0419,201.0916	[45]
331	glycyrrhetic acid 3-*O*-glucuronide	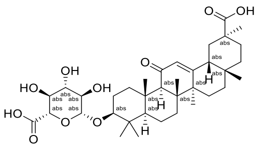	Ter	GR	39.45	$[M+H]^+$<br>$[M-H]^-$	453.3359,357.2422,<br>285.2203,121.1012<br>580.9614,521.2628,<br>469.3308,322.6431	[35]

332	glyasperin A	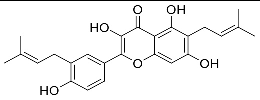	Fla	GR	39.46	[M-H]$^-$	403.9289,353.1024,<br>312.0273,280.0371	[39]
333	1-methoxyphaseollin	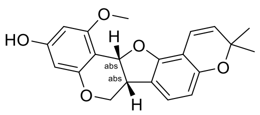	Fla	GR	39.47	[M-H]$^-$	294.4448,243.1023,<br>227.0710,125.0232	[54]
334	licochalcone D	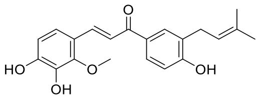	Fla	GR	39.53	[M+H]$^+$	338.3410,311.0542,<br>193.0494,135.0440	[39]
335	wighteone	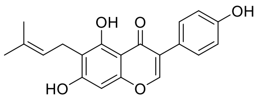	Fla	GR	39.54	[M+H]$^+$<br>[M-H]$^-$	321.2453,311.0548,<br>209.1647,114.0916<br>321.0765,309.1127,<br>253.0500,209.0596	[54]
336	2'-*O*-demethylbidwillol B	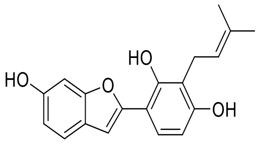	Op	GR	39.54	[M+H]$^+$	293.1166,278.0932,<br>263.0694,95.0163	[35]
337	glycyrol	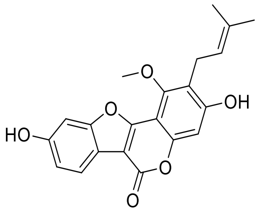	Fla	GR	39.63	[M+H]$^+$<br>[M-H]$^-$	337.0697,227.0702,<br>167.0337,91.0547<br>335.0560,307.0247,<br>295.0245,254.0220	[57]
338	3-hydroxyglabrol	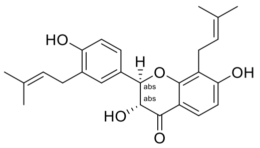	Fla	GR	39.65	[M-H]$^-$	387.2756,371.2437,<br>150.9878,93.0001	[60]
339	kumatakenin	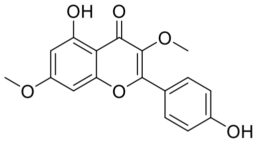	Fla	GR	39.72	[M+H]$^+$<br>[M-H]$^-$	255.0647,227.0699,<br>153.0180,60.0452<br>295.0606,283.0609,<br>267.0663,239.0712	[35]
340	eriodictyol	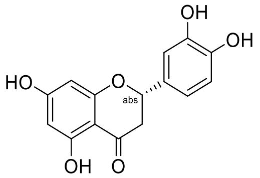	Fla	HE	39.73	[M+H]$^+$<br>[M-H]$^-$	271.0959,229.0855,<br>163.0388,153.0181<br>272.0326,258.0119,<br>216.0419,155.1432	[45]
341	licoflavone B	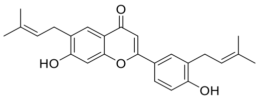	Fla	GR	39.74	[M+H]$^+$	358.2020,323.1262,<br>267.0647,195.0430	[35]
342	gancaonin U	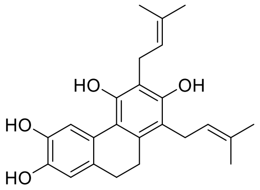	Op	GR	39.76	[M-H]$^-$	/	[42]
343	dehydroglyceollin I	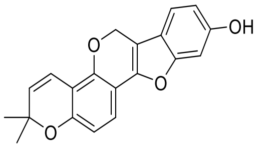	Fla	GR	39.78	[M+H]$^+$<br>[M-H]$^-$	306.0873,187.0752,<br>159.0803,147.0439<br>303.0658,289.0504,<br>243.0657,161.0233	[35]
344	tectorigenin	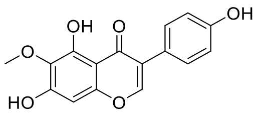	Fla	PLR	39.80	[M+H]$^+$<br>[M-H]$^-$	283.0598,255.0646,<br>227.0698,199.0748<br>284.0317,267.0301,<br>240.0422,212.0468	[28]

345	derrone	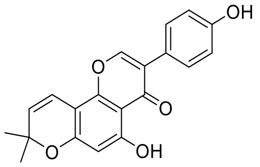	Fla	GR	39.87	[M+H]+<br>[M-H]-	309.1118,267.0650, 225.0545,91.0549<br>319.0606,305.0436, 278.3866,158.8393	[48]
346	abyssinone II	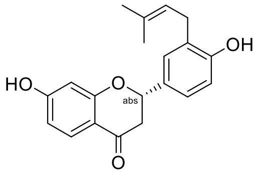	Fla	GR	39.91	[M+H]+<br>[M-H]-	269.0804,241.0850, 135.0440,95.0163<br>308.1031,201.0914, 187.0761,135.0441	[48]
347	corylin	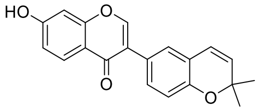	Fla	PLR	39.96	[M+H]+	306.0870,279.0649, 265.0488,137.0232	[28]
348	ephedradine A	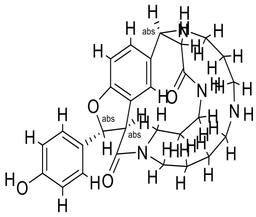	Alk	HE	39.98	[M+H]+	465.2870,394.2122, 219.1489,120.0809	[25]
349	kudzusapogenol A	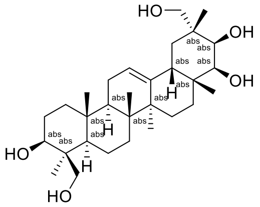	Ter	PLR	40.02	[M-H]-	/	[28]
350	kanzonol Y	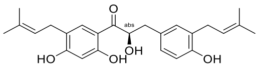	Fla	GR	40.04	[M-H]-	391.2520,373.2436, 235.0971,177.0912	[48]
351	kanzonol W	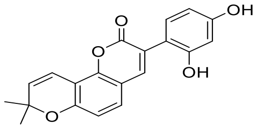	Fla	GR	40.11	[M+H]+<br>[M-H]-	321.1119,281.0443, 253.0488,163.0388<br>320.0677,291.1024, 199.0758,135.0078	[48]
352	isoglycyrol	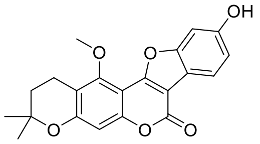	Fla	GR	40.13	[M+H]+<br>[M-H]-	349.1074,325.0702, 291.0630,167.0338<br>349.0708,309.0393, 216.0423,192.0055	[35]
353	licoisoflavone B	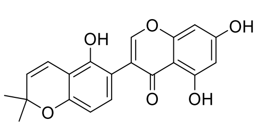	Fla	GR	40.17	[M+H]+<br>[M-H]-	311.0558,299.0544, 153.0180,95.0163<br>337.0660,283.0974, 241.0864,199.0756	[35]
354	6,8-diprenylgenistein	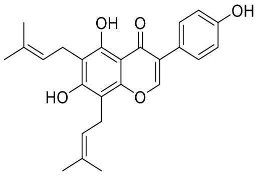	Fla	GR	40.26	[M+H]+<br>[M-H]-	339.1198,283.0596, 237.0534,91.0547<br>387.2755,371.2439, 281.0460,150.9878	[35]
355	parvisoflavone A	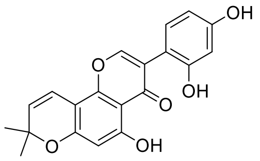	Fla	GR	40.29	[M+H]+	335.0906,325.1068, 191.0328,153.0180	[48]
356	licoricidin	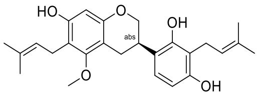	Fla	GR	40.30	[M+H]+	369.1328,313.0703, 175.0388,139.0389	[35]
357	kanzonol C	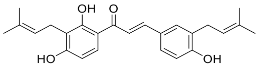	Fla	GR	40.31	[M+H]+	/	[48]

358	emodin	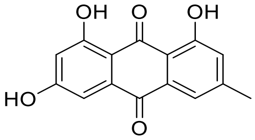	Ant	PCR	40.43	[M+H]+<br>[M-H]-	243.0650,229.0493,<br>197.0596,173.0591<br>241.0503,225.0551,<br>197.0597,181.0647	[27]
359	apigenin	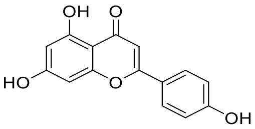	Fla	LJT	40.43	[M+H]+<br>[M-H]-	229.0495,201.0543,<br>173.0597,91.0548<br>241.0501,225.0551,<br>210.0314,181.0644	[26]
360	genistein	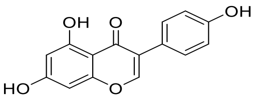	Fla	PLR	40.44	[M+H]+<br>[M-H]-	243.0642,229.0495,<br>201.0543,371.0596<br>241.0504,225.0550,<br>197.0597,181.0647	[28]
361	lupalbigenin	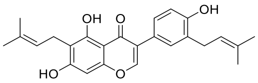	Fla	GR	40.45	[M+H]+	373.1034,283.0597,<br>213.0541,149.0232	[35]
362	angustone A	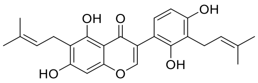	Fla	GR	40.51	[M-H]-	404.9251,352.0951,<br>269.0453,201.0913	[35]
363	dehydroglyasperin D	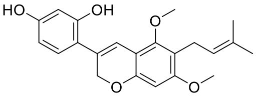	Fla	GR	40.59	[M+H]+<br>[M-H]-	313.0700,295.0597,<br>197.0441,179.0337<br>351.9669,322.9651,<br>269.0455,240.0420	[39]
364	corymbosin	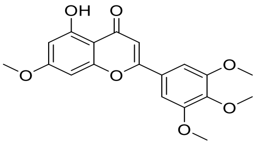	Fla	LJT	40.63	[M+H]+	329.0648,313.0695,<br>269.0804,95.0163	[26]
365	euchrenone a5	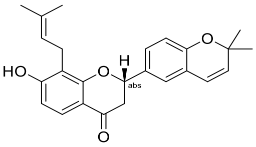	Fla	GR	40.64	[M+H]+<br>[M-H]-	358.2020,267.0647,<br>239.0701,149.0236<br>319.0978,298.0473,<br>266.0580,195.1691	[35]
366	paratocarpin L	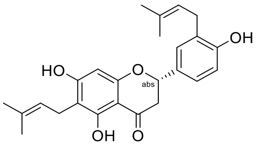	Fla	GR	40.75	[M-H]-	/	[35]
367	diisobutyl phthalate	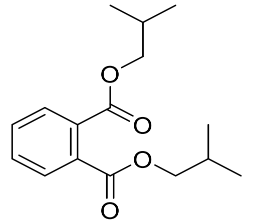	Oth	PLR	40.79	[M+H]+<br>[M-H]-	167.0340,149.0232,<br>121.0284,57.0706<br>245.3889,193.7951,<br>134.0361,121.0283	[28]
368	glyurallin B	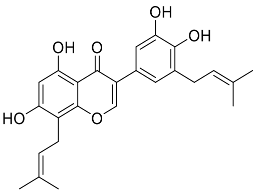	Fla	GR	40.79	[M+H]+	/	[35]
369	angustone B	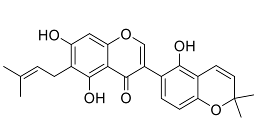	Fla	GR	40.82	[M+H]+<br>[M-H]-	365.1014,309.0388,<br>281.0439,140.0342<br>402.9280,375.0866,<br>363.0872,308.0323	[35]
370	licoagrocarpin	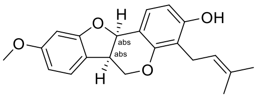	Fla	GR	40.93	[M+H]+	/	[48]
371	palmitic acid	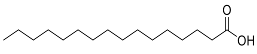	Oa	PCR	41.04	[M-H]-	170.3186,162.0524,	[27]

							116.9273,74.0233	
372	2'-hydroxyisolupalbigenin	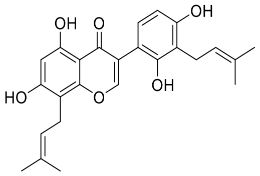	Fla	GR	41.11	[M-H]-	404.9261,363.0872,<br>227.0711,193.0862	[54]
373	butesuperin A	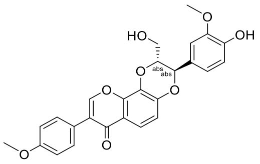	Fla	PLR	41.15	[M+H]+	445.1275,283.0597,<br>255.0647,161.0594	[28]
374	luteolin	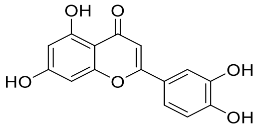	Fla	HE	41.16	[M-H]-	268.9432,257.0451,<br>242.0536,196.0504	[45]
375	sophoracoumestan A	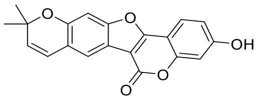	Fla	PLR	41.19	[M+H]+	320.0672,307.0952,<br>292.0722,137.0237	[28]
376	glycyrrhetic acid	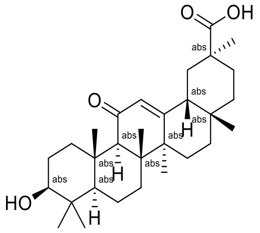	Ter	GR	41.41	[M+H]+	317.2107,269.0803,<br>189.1636,121.1013	[39]
377	8-prenylphaseollinisoflavan	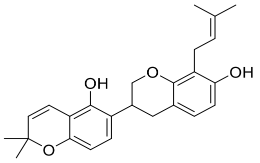	Fla	GR	41.74	[M+H]+<br>[M-H]-	339.0701,269.0807,<br>167.0337,149.0232<br>289.1443,271.1335,<br>187.0393,119.0490	[35]
378	stearic acid	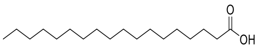	Oa	PCR	41.75	[M-H]-	/	[27]
379	puerarol	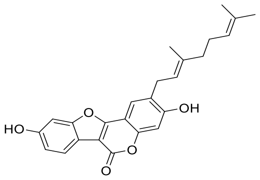	Fla	PLR	41.75	[M+H]+<br>[M-H]-	319.0950,281.0439,<br>209.0591,171.0138<br>387.2754,371.2439,<br>333.0764,150.9877	[28]
380	hederagenin	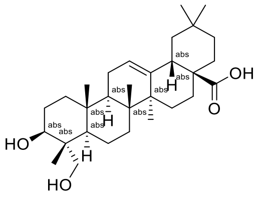	Ter	LJT	41.79	[M+H]+<br>[M-H]-	310.8371,189.1640,<br>133.1015,59.0162<br>429.2146,403.1549,<br>319.0597,280.0376	[26]

(The Type column in the table of Fla: flavonoids, Fg: flavonoid glycoside, Ter: terpenes, Alk: alkaloids, Pa: phenolic acids, Oa: organic acids, Op: other phenols, Ant: anthraquinones, Oth: other compounds. The Source column in the table of LJT: *Lonicerae Japonicae Flos*, PLR: *Puerariae Lobatae Radix*, PCR: *Polygoni Cuspidati Rhizoma Et Radix*, HE: *Ephedrae Herba*, GR: *Glycyrrhizae Radix Et Rhizoma*, ASA: *Armeniacae Semen Amarum*)

Table S2. Recovery of Six Representative Components

Main components	Recoveries				
	Initial (mg)	Added (mg)	Detected (mg)	Recovery (%)	RSD (%)
Chlorogenic acid	0.3316	0.2653	0.5916	96.79%	1.71%
	0.3316	0.3316	0.6663	99.96%	1.59%
	0.3316	0.3979	0.7208	96.99%	0.92%
Puerarin	0.2730	0.2184	0.5558	103.13%	1.17%
	0.2730	0.2730	0.5959	97.21%	0.46%
	0.2730	0.3276	0.6477	96.83%	1.50%
3′-Methoxypuerain	0.0973	0.0778	0.1904	97.44%	1.13%
	0.0973	0.0973	0.2091	97.25%	0.39%
	0.0973	0.1167	0.2318	100.44%	1.79%
Polydatin	0.1029	0.0823	0.1847	102.33%	0.33%
	0.1029	0.1029	0.2062	102.77%	0.99%
	0.1029	0.1235	0.2260	101.68%	0.97%
Glycyrrhizic acid	0.0318	0.0254	0.0803	97.20%	1.05%
	0.0318	0.0318	0.0866	97.62%	1.52%
	0.0318	0.0382	0.0943	101.42%	3.12%
Emodin	0.0415	0.0332	0.0770	101.19%	0.75%
	0.0415	0.0415	0.0859	102.52%	0.99%
	0.0415	0.0497	0.0941	101.94%	1.05%

Table S3. The content of representative components of YPG under different extraction conditions

Representative components	1# Yield (mg/g)[a]	2# Yield (mg/g)[a]	3# Yield (mg/g)[a]	4# Yield (mg/g)[a]
chlorogenic acid	18.87	2.25	13.93	33.16
puerarin	36.03	25.51	30.31	27.30
3′-methoxypuerarin	5.60	7.73	8.42	9.73
polydatin	6.14	19.11	7.25	10.29
glycyrrhizic acid	1.76	0.44	1.22	3.18
emodin	1.58	1.89	0.47	4.15

[a] **Extraction condition 1#:** 90% methanol as solvent, ultrasonic bath (300 W, 40 kHz, 50 °C, 30 min)

Extraction condition 2#: 50% methanol as solvent, ultrasonic bath (300 W, 40 kHz, 50 °C, 60 min)

Extraction condition 3#: 50% methanol as solvent, ultrasonic bath (100 W, 40 kHz, 50 °C, 30 min)

Extraction condition 4# (the optimized method): 50% methanol as solvent, ultrasonic bath (300 W, 40 kHz, 50 °C, 30 min)

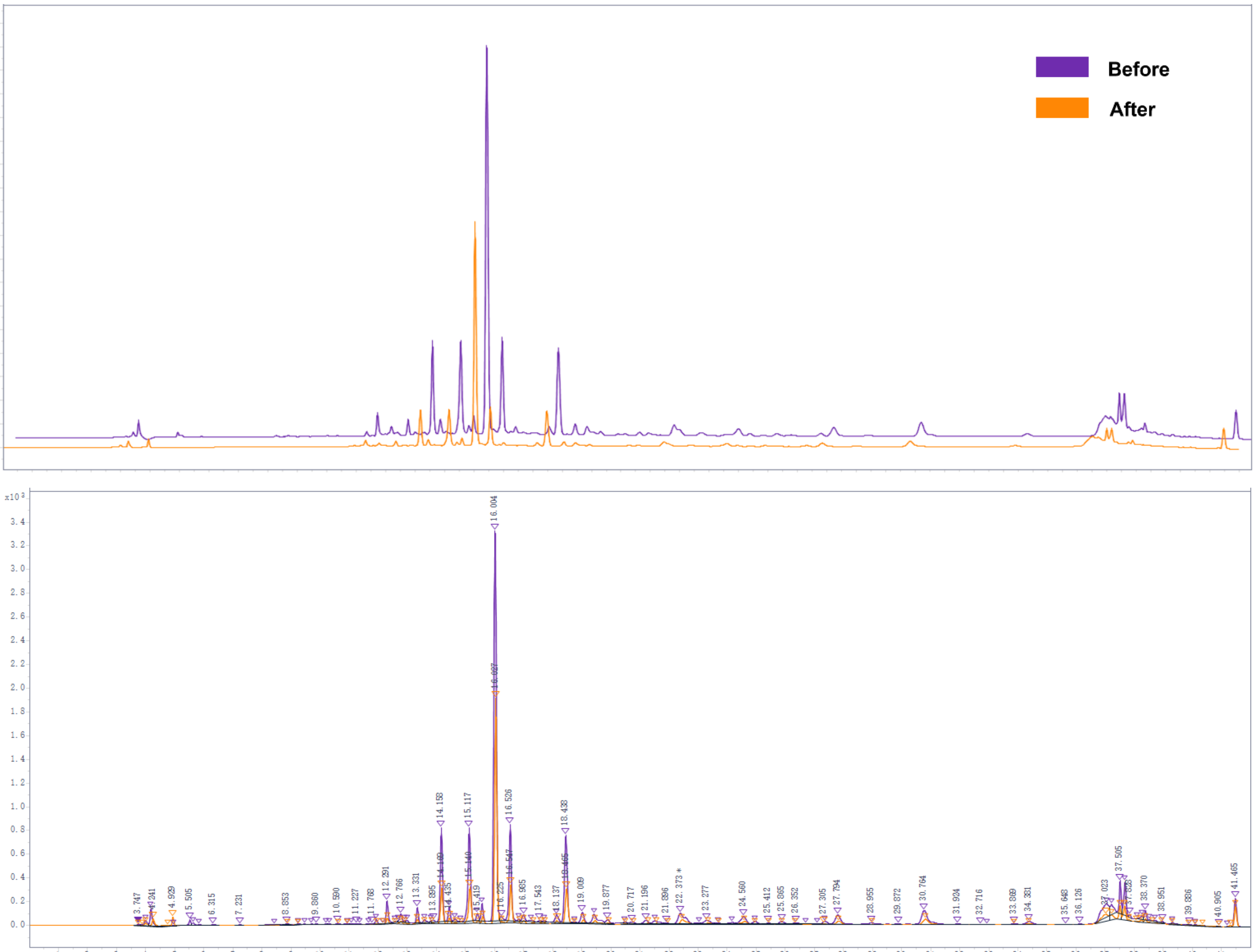

Figure S1. The HPLC chromatogram at 254 nm of YPG before and after reaction with DPPH

References

[25] Li, J.; Fang, L.; Zhang, Y.; Wang, X.; Zhang, Q. Research progress on chemical constituents and pharmacological activities of Herba Ephedrae. *Modern Chinese Medicine.* **2012**,*14(07):*21-27.(in Chinese)

[26] Shang, X.; Pan, H.; Li, M.; Miao, X.; Ding, H. *Lonicera japonica Thunb.*: ethnopharmacology, phytochemistry and pharmacology of an important traditional Chinese medicine. *J Ethnopharmacol.* **2011**,*138(1)*:1-21.

[27] Liang, C.; Wang, S.; Chen, S.; Wang, Y.; Li, J.; Chang, Y. Research development on chemical composition and pharmacology of *Polygoni Cuspidati Rhizoma et Radix*. *Chinese Traditional and Herbal Drugs*. **2022**,*53(04)*:1264-1276. (in Chinese)

[28] Zhu ,W.; Li, J.; Meng, X.; Zhang, P.; Wu, W.; Liu, R. Research advances in chemical constituents and pharmacological activities of Pueraria genus. *China Journal of Chinese Materia Medica*. 2021,*46(06)*:1311-1331. (in Chinese)

[29] Gong, X.; Liu, W.; Cao, L.; Yu, J.; Si, D.; Li, J.; Tu, P.; Li, J.; Song, Y.Rapid chemome profiling of chemical components of Lonicerae Japonicae Flos using DI-MS /MSALL. *Chin J Chin Mater Med*,**2021**,*46(09)*:2220-2228. (in Chinese)

[30] Miao, S.; Zhang, Q.; Bi, X.; Cui, .; Wang, M. A review of the phytochemistry and pharmacological activities of Ephedra herb. *Chin J Nat Med*. **2020**,*18(5)*:321-344.

[31] Li, Z.Test of thrombolysis effect and study on chemical composition of non-ephedrine alkaloids part in *Ephedra sinica* Stapf. Beijing University of Chemical Technology.**2004**. (in Chinese)

[32] Zang, X.; Shang, M.; Xu, F.; Liang, J.; Wang, X.; Mikage, M.; Cai, S. A-type proanthocyanidins from the stems of Ephedra sinica (Ephedraceae) and their antimicrobial activities. *Molecules*. **2013**,*18(5)*:5172-5189.

[33] Li, R. Chinese Formula Mahuangtang Chemical Composition Analysis and Spectrum-Effect Relation Research. Chengdu University of Traditional Chinese Medicine.**2015**. (in Chinese)

[34] Lin, Kai. Studies on fingerprint for *Herba Ephedrae* and determination for alkaloids contents in *Herba Ephedrae* with HPLC. Chongqing Medical University. **2006**. (in Chinese)

[35] Song, W.; Qiao, X.; Chen, K.; Wang, Y.; Ji, S.; Feng, J.; Li, K.; Lin, Y.; Ye, M. Biosynthesis-Based Quantitative Analysis of 151 Secondary Metabolites of Licorice To Differentiate Medicinal Glycyrrhiza Species and Their Hybrids. *Anal Chem*. **2017**,*89(5)*:3146-3153.

[36] Xiong, L.; Jin, Y.; Wang, Y.; Zhao, H.; Li, J.; Pu, G.; Zhang, L.; Yang, H.; Zhang, Y.; Zhang, L. Research progress on chemical constituents, pharmacological activities and in vivo metabolism of phenolic acids in Lonicera japonica Thunb. *Chinese Traditional Patent Medicine*. **2022**,*44(03)*:864-871. (in Chinese)

[37] Zhang, Y.; Huang, X.; Chen, Y.; Li, J.; Yu, K. Chemical constituents and their biosynthesis mechanisms of Polygonum cuspidatum. *Chin J Chin Mater Med*,**2020**,*45(18)*:4364-4372. (in Chinese)

[38] Cai, Z.; Liao, H.; Wang, C.; Chen, J.; Tan, M.; Mei, Y.; Wei, L.; Chen, H.; Yang, R.; Liu, X. A comprehensive study of the aerial parts of *Lonicera japonica* Thunb. based on metabolite profiling coupled with PLS-DA. *Phytochem Anal*. **2020**,*31(6)*:786-800.

[39] Li, N.; Zhang, C.; Zhong, G.; Xiu, L.; Liu, H.; Chen, S.; Chen, F.; Li, M.; Liao, W.; Ren, Y. Research progress on chemical constituents and pharmacological effects of different varieties of *Glycyrrhizae Radix* et *Rhizoma* and predictive analysis of quality markers. *Chin Tradit Herbal Drugs*,**2021**,*52(24)*:7680-7692. (in Chinese)

[40] Ma, P. Pharmacognostic studies of *Polygonum cuspidatum* Sieb. Et Zucc.(Polydonaceae). Peking Union Medical College.**2013**. (in Chinese)

[41] Wu, J.; Wang, C.; Yu, H. Chemical Constituents and Pharmacological Effect of Lonicerae Japonicae Flos. *Chin J Exp Tradit Med Form*,**2019**,*25(04)*:225-234. (in Chinese)

[42] Xiang, C.; Qiao, X.; Ye, M.; Guo, D. Classification and distribution analysis of components in *Glycyrrhiza* using licorice compounds database. *Acta Pharm Sin*,**2012**,*47(08)*:1023-1030. (in Chinese)

[43] Tan, G.; Zhu, Z.; Zhang, H.; Zhao, L.; Liu, Y.; Dong, X.; Lou, Z.; Zhang, G.; Chai, Y. Analysis of phenolic and triterpenoid compounds in licorice and rat plasma by high-performance liquid chromatography diode-array detection, time-of-flight mass spectrometry and quadrupole ion trap mass spectrometry. *Rapid Commun Mass Spectrom*. **2010**,*24(2)*:209-218.

[44] Wang, X.; Qin, Y.; Sun, J.; Hua, l.; Luo, W. Research progress on chemical constituents, pharmacological actives, clinical applications and quality control of Polygoni cus pidati folium. *Asia-Pacific Traditional Medicine*,**2019**,*15(10)*:196-200. (in Chinese)

[45] Zhang, Y.; Yang, J.; Song, J.; Gao, S.; Zhang, Q.; Wang, B.; Zhao, Y. Discussion on mechanism of *Ephedra Herba* in treatment of heart failure based on network pharmacology. *Drug Evaluation Research*,**2021**,*44(10)*:2189-2202. (in Chinese)

[46] Liu, G.; Wu, Y.; Liu, Y.; Li, X.; Yang, W. Chemical Constituents in Glycyrrhiza uralensis: A Review. *Modern Chinese Medicine*,**2021**,*23(11)*:2006-2016. (in Chinese)

[47] Cheng, M.; Zhang, J.; Yang, L.; Shen, S.; Li, P.; Yao, S.; Qu, H.; Li, J.; Yao, C.; Wei, W.; Guo, D. Recent advances in chemical analysis of licorice (Gan-Cao). *Fitoterapia*. **2021**,*149*:104803.

[48] Li, K.; Ji, S.; Song, W.; Kuang, Y.; Lin, Y.; Tang, S.; Cui, Z.; Qiao, X.; Yu, S.; Ye, M. Glycybridins A-K, Bioactive Phenolic Compounds from Glycyrrhiza glabra. *J Nat Prod*. **2017**,*80(2)*:334-346.

[49] Montoro, P.; Maldini, M.; Russo, M.; Postorino, S.; Piacente, S.; Pizza, C. Metabolic profiling of roots of liquorice (Glycyrrhiza glabra) from different geographical areas by ESI/MS/MS and determination of major metabolites by LC-ESI/MS and LC-ESI/MS/MS. *J Pharm Biomed Anal*. **2011**,*54(3)*:535-544.

[50] Frattaruolo, L.; Carullo, G.; Brindisi, M.; Mazzotta, S.; Bellissimo, L.; Rago, V.; Curcio, R.; Dolce, V.; Aiello, F.; Cappello, A.R. Antioxidant and Anti-Inflammatory Activities of Flavanones from *Glycyrrhiza glabra* L. (licorice) Leaf Phytocomplexes: Identification of Licoflavanone as a Modulator of NF-kB/MAPK Pathway. *Antioxidants (Basel).* **2019**,*8(6)*:186.

[51] Cao, Y. Research on chemical components and α-glucosidase inhibiting activity of Huzhang. Jilin Agricultural University.**2015**. (in Chinese)

[52] Qiu, Z.; Liu, Z.; Pang, J.; Wu, H.; Liu, X.; Yang, Z.; Li, X.; Chen, J. A network pharmacology study with molecular docking to investigate the possibility of licorice against posttraumatic stress disorder. *Metab Brain Dis*. **2021**,*36(7)*:1763-1777.

[53] Lu, Y.; Ding, H.; Shi, Z.; Lin, H.; Zhang, G. Study on the mechanism of action of *Ephedra Herba* Decoction against influenza A virus based on network pharmacology. *TMR Modern Herb Med*. **2022**,*5(2)*: 0502001.

[54] Qiao, X.; Song, W.; Ji, S.; Wang, Q.; Guo, D.; Ye, M. Separation and characterization of phenolic compounds and triterpenoid saponins in licorice (*Glycyrrhiza uralensis*) using mobile phase-dependent reversed-phase×reversed-phase comprehensive two-dimensional liquid chromatography coupled with mass spectrometry. *J Chromatogr A*. **2015**,*1402*:36-45.

[55] Qiao, X.; Liu, C.; Ji, S.; Lin, X.; Guo, D.; Ye, M. Simultaneous determination of five minor coumarins and flavonoids in *Glycyrrhiza uralensis* by solid-phase extraction and high-performance liquid chromatography/electrospray ionization tandem mass spectrometry. *Planta Med*. **2014**,*80(2-3)*:237-242.

[56] Li, X.; Liu, H.; Shao, Y.; Ma, G.; Song, D.; Xu, G.; Wang, Z. Wide Identification of the Compounds in Licorice and Exploration of the Mechanism for Prostatitis Treatment by Combining UHPLC-LTQ-Orbitrap MS with Network Pharmacology. *ChemistrySelect*, **2019**,*4(11)*, 3011–3017.

[57] Wang, K.; Meng, Y.; Lu, X.; Pang, X.; Wang, Y.; Ji, S.; Deng, Z.; Ma, P. Study on the Mechanism of Mahuang(*Ephedrae Herba*)-Xingren (*Armeniacae Semen Amarum*)in the Treatment of Bronchial Asthma. *Journal of Liaoning University of Traditional Chinese Medicine*,**2021**,*23(06)*:205-212. (in Chinese)

[58] Fan, J.; Kuang, Y.; Dong, Z.; Yi, Y.; Zhou, Y.; Li, B.; Qiao, X.; Ye, M. Prenylated Phenolic Compounds from the Aerial Parts of Glycyrrhiza uralensis as PTP1B and α-Glucosidase Inhibitors. *J Nat Prod*. **2020**,*83(4)*:814-824.

[59] Chin, Y.W.; Jung, H.A.; Liu, Y.; Su, B.N.; Castoro, J.A.; Keller, W.J.; Pereira, M.A.; Kinghorn, A.D. Anti-oxidant constituents of the roots and stolons of licorice (*Glycyrrhiza glabra*). *J Agric Food Chem*. **2007**,*55(12)*:4691-4697.

[60] Mitscher, L.A.; Park, Y.H.; Clark, D.; Beal, J.L. Antimicrobial agents from higher plants. Antimicrobial isoflavanoids and related substances from Glycyrrhiza glabra L. var. typica. *J Nat Prod*. **1980**,*43(2)*:259-269.
